# Ultrahigh-energy gamma-ray emission associated with black hole–jet systems

**DOI:** 10.1093/nsr/nwaf496

**Published:** 2025-11-16

**Authors:** Zhen Cao, Felix Aharonian, Yun-Xiang Bai, Yi-Wei Bao, Denis Bastieri, Xiao-Jun Bi, Yu-Jiang Bi, Wen-Yi Bian, Anatoly V Bukevich, Chengmiao Cai, Wen-Yu Cao, Zhe Cao, Jin Chang, Jin-Fan Chang, Aming Chen, En-Sheng Chen, Guohai Chen, Hua-Xi Chen, Liang Chen, Long Chen, Ming-Jun Chen, Ma-Li Chen, Qi-Hui Chen, Shi Chen, Su-Hong Chen, Song-Zhan Chen, Tian-Lu Chen, Xiao-Bin Chen, Xuejian Chen, Yang Chen, Ning Cheng, Yao-Dong Cheng, Ming Chung Chu, Ming-Yang Cui, Shu-Wang Cui, Xiao-Hong Cui, Yi-Dong Cui, Ben-Zhong Dai, Hong-Liang Dai, Zigao Dai, Danzeng Luobu, Yang-Xuan Diao, Xu-Qiang Dong, Kai-Kai Duan, Jun-Hui Fan, Yi-Zhong Fan, Jun Fang, Jian-Hua Fang, Kun Fang, Cun-feng Feng, Hua Feng, Li Feng, Shaohui Feng, Xiao-ting Feng, Yi Feng, You-liang Feng, Stefano Gabici, Bo Gao, Chuan-dong Gao, Qi Gao, Wei Gao, Wei-kang Gao, Maomao Ge, Ting-Ting Ge, Lisi Geng, Gwenael Giacinti, Guanghua Gong, Quanbu Gou, Min-Hao Gu, Fu-Lai Guo, Jing Guo, Xiao-Lei Guo, Yi-Qing Guo, Ying-Ying Guo, Yi-Ang Han, Otto A Hannuksela, Mariam Hasan, Hui-Hai He, Hao-Ning He, Jia-Yin He, Xinyu He, Yu He, Sergio Hernández-Cadena, Bo-Wen Hou, Chao Hou, Xian Hou, Hong-Bo Hu, Shi-Cong Hu, Chen Huang, Dai-Hui Huang, Jiajun Huang, Tian-Qi Huang, Wen-Jun Huang, Xing-Tao Huang, Xiao-Yuan Huang, Yong Huang, Yi-Yun Huang, Xiao-Lu Ji, Huan-Yu Jia, Kang Jia, Hou-Bing Jiang, Kun Jiang, Xiao-Wei Jiang, Ze-Jun Jiang, Min Jin, Samy Kaci, Ming-Ming Kang, Ivan Karpikov, Dmitry Khangulyan, Denis Kuleshov, Kirill Kurinov, Bing-Bing Li, Cheng Li, Cong Li, Dan Li, Fei Li, Haibo Li, Huicai Li, Jian Li, Jie Li, Kai Li, Long Li, Rong-Lan Li, Si-Da Li, Tian-Yang Li, Wen-Lian Li, Xiu-Rong Li, Xin Li, Yuan Li, Yizhuo Li, Zhe Li, Zhuo Li, En-Wei Liang, Yun-Feng Liang, Su-Jie Lin, Bing Liu, Cheng Liu, Dong Liu, Dang-Bo Liu, Hu Liu, Hai-Dong Liu, Jia Liu, Jia-Li Liu, Ji-Ren Liu, Mao-Yuan Liu, Ruo-Yu Liu, Si-Ming Liu, Wei Liu, X Liu, Yi Liu, Yu Liu, Yi-Nong Liu, Yu-Qing Lou, Qing Luo, Yu Luo, Hong-Kui Lv, Bo-Qiang Ma, Ling-Ling Ma, Xin-Hua Ma, Ji-Rong Mao, Zhen Min, Warit Mitthumsiri, Guo-Bin Mou, Hui-Jun Mu, Andrii Neronov, Kenny Chun Yu NG, Ming-Yang Ni, Lin Nie, Le-Jian Ou, Petchara Pattarakijwanich, Zhi-Yuan Pei, Jin-Can Qi, Meng-Yao Qi, Jia-Jun Qin, Ali Raza, Chong-Yang Ren, David Ruffolo, Alejandro Sáiz, Dmitri Semikoz, Lang Shao, Oleg Shchegolev, Yun-Zhi Shen, Xiang-Dong Sheng, Zhaodong Shi, Fu-Wen Shu, Hui-Chao Song, Yuri V Stenkin, Vladimir Stepanov, Yang Su, Dongxu Sun, Hao Sun, Qinning Sun, Xiaona Sun, Zhibin Sun, Nabeel Hussain Tabasam, Jumpei Takata, Pak Hin Thomas Tam, Hong-Bin Tan, Qingwen Tang, Ruiyi Tang, Zebo Tang, Wenwu Tian, Chaonan Tong, Li-Hong Wan, Chao Wang, Guangwei Wang, Hongguang Wang, Jiancheng Wang, Ke Wang, Kai Wang, Liping Wang, Lingyu Wang, Lu-Yao Wang, Ran Wang, Wei Wang, Xianggao Wang, Xin-Jian Wang, Xiang-Yu Wang, Yang Wang, Yu-Dong Wang, Zhong-Hai Wang, Zhong-Xiang Wang, Zheng Wang, Da-Ming Wei, Jun-Jie Wei, Yong-Jian Wei, Tao Wen, Shan-Shan Weng, Chao-Yong Wu, Han-Rong Wu, Qing-Wen Wu, Sha Wu, Xue-Feng Wu, Yu-Sheng Wu, Shao-qiang Xi, Jie Xia, Jun-Ji Xia, Guang-man Xiang, Di-xuan Xiao, Gang Xiao, Yu-liang Xin, Yi Xing, Ding-rong Xiong, Zeng Xiong, Dong-lian Xu, Reng-Feng Xu, Ren-Xin Xu, Wei-Li Xu, Liang Xue, Da-Hai Yan, Jing-Zhi Yan, Tian Yan, Chao-Wen Yang, Chu-Yuan Yang, Feng-Fan Yang, Li-Li Yang, Ming-Jie Yang, Rui-Zhi Yang, Wen-Xin Yang, Zihang Yang, Zhi-Guo Yao, Xuan-Ang Ye, Li-Qiao Yin, Na Yin, Xiao-Hao You, Zhi-Yong You, Yan-Hong Yu, Qiang Yuan, Hua Yue, Hou-Dun Zeng, Ting-Xuan Zeng, Wei Zeng, Xiangtao Zeng, Min Zha, Bin-Bin Zhang, Bing Theodore Zhang, Chao Zhang, Feng Zhang, Hong-Fei Zhang, Hai-Ming Zhang, Heng-Ying Zhang, Jian-Li Zhang, Li Zhang, Peng-Fei Zhang, Pei-Pei Zhang, Rui Zhang, Shao-Ru Zhang, Shou-Shan Zhang, Weiyan Zhang, Xiao Zhang, Xiao-Peng Zhang, Yi Zhang, Yong Zhang, Zhi-Peng Zhang, Jing Zhao, Lei Zhao, Li-Zhi Zhao, Shi-Ping Zhao, Xiao-Hong Zhao, Zihao Zhao, Fu Zheng, Wen-Juan Zhong, Bin Zhou, Hao Zhou, Jia-Neng Zhou, Meng Zhou, Ping Zhou, Rong Zhou, Xiao-Xi Zhou, Xun-Xiu Zhou, Ben-Yang Zhu, Cheng-Guang Zhu, Feng-Rong Zhu, Hui Zhu, Ke-Jun Zhu, Yuan-Chuan Zou, Xiong Zuo

**Affiliations:** State Key Laboratory of Particle Astrophysics & Experimental Physics Division & Computing Center, Institute of High Energy Physics, Chinese Academy of Sciences, Beijing 100049, China; School of Physical Sciences, University of Chinese Academy of Sciences, Beijing 100049, China; TIANFU Cosmic Ray Research Center, Chengdu 610213, China; TIANFU Cosmic Ray Research Center, Chengdu 610213, China; School of Physical Sciences, University of Science and Technology of China, Hefei 230026, China; Yerevan State University, Yerevan 0025, Armenia; Max-Planck-Institut for Nuclear Physics, Heidelberg 69029, Germany; State Key Laboratory of Particle Astrophysics & Experimental Physics Division & Computing Center, Institute of High Energy Physics, Chinese Academy of Sciences, Beijing 100049, China; TIANFU Cosmic Ray Research Center, Chengdu 610213, China; Tsung-Dao Lee Institute & School of Physics and Astronomy, Shanghai Jiao Tong University, Shanghai 200240, China; Center for Astrophysics, Guangzhou University, Guangzhou 510006, China; State Key Laboratory of Particle Astrophysics & Experimental Physics Division & Computing Center, Institute of High Energy Physics, Chinese Academy of Sciences, Beijing 100049, China; School of Physical Sciences, University of Chinese Academy of Sciences, Beijing 100049, China; TIANFU Cosmic Ray Research Center, Chengdu 610213, China; State Key Laboratory of Particle Astrophysics & Experimental Physics Division & Computing Center, Institute of High Energy Physics, Chinese Academy of Sciences, Beijing 100049, China; TIANFU Cosmic Ray Research Center, Chengdu 610213, China; Tsung-Dao Lee Institute & School of Physics and Astronomy, Shanghai Jiao Tong University, Shanghai 200240, China; Institute for Nuclear Research of Russian Academy of Sciences, Moscow 117312, Russia; School of Physical Science and Technology & School of Information Science and Technology, Southwest Jiaotong University, Chengdu 610031, China; School of Physical Sciences, University of Science and Technology of China, Hefei 230026, China; School of Physical Sciences, University of Science and Technology of China, Hefei 230026, China; State Key Laboratory of Particle Detection and Electronics, Beijing 100049, China; Key Laboratory of Dark Matter and Space Astronomy & Key Laboratory of Radio Astronomy, Purple Mountain Observatory, Chinese Academy of Sciences, Nanjing 210023, China; State Key Laboratory of Particle Astrophysics & Experimental Physics Division & Computing Center, Institute of High Energy Physics, Chinese Academy of Sciences, Beijing 100049, China; TIANFU Cosmic Ray Research Center, Chengdu 610213, China; State Key Laboratory of Particle Detection and Electronics, Beijing 100049, China; Tsung-Dao Lee Institute & School of Physics and Astronomy, Shanghai Jiao Tong University, Shanghai 200240, China; State Key Laboratory of Particle Astrophysics & Experimental Physics Division & Computing Center, Institute of High Energy Physics, Chinese Academy of Sciences, Beijing 100049, China; TIANFU Cosmic Ray Research Center, Chengdu 610213, China; Center for Astrophysics, Guangzhou University, Guangzhou 510006, China; Research Center for Astronomical Computing, Zhejiang Laboratory, Hangzhou 311121, China; Shanghai Astronomical Observatory, Chinese Academy of Sciences, Shanghai 200030, China; School of Physical Science and Technology & School of Information Science and Technology, Southwest Jiaotong University, Chengdu 610031, China; State Key Laboratory of Particle Astrophysics & Experimental Physics Division & Computing Center, Institute of High Energy Physics, Chinese Academy of Sciences, Beijing 100049, China; TIANFU Cosmic Ray Research Center, Chengdu 610213, China; State Key Laboratory of Particle Astrophysics & Experimental Physics Division & Computing Center, Institute of High Energy Physics, Chinese Academy of Sciences, Beijing 100049, China; TIANFU Cosmic Ray Research Center, Chengdu 610213, China; State Key Laboratory of Particle Detection and Electronics, Beijing 100049, China; School of Physical Science and Technology & School of Information Science and Technology, Southwest Jiaotong University, Chengdu 610031, China; School of Physics and Astronomy, Yunnan University, Kunming 650091, China; State Key Laboratory of Particle Astrophysics & Experimental Physics Division & Computing Center, Institute of High Energy Physics, Chinese Academy of Sciences, Beijing 100049, China; School of Physical Sciences, University of Chinese Academy of Sciences, Beijing 100049, China; TIANFU Cosmic Ray Research Center, Chengdu 610213, China; State Key Laboratory of Particle Astrophysics & Experimental Physics Division & Computing Center, Institute of High Energy Physics, Chinese Academy of Sciences, Beijing 100049, China; TIANFU Cosmic Ray Research Center, Chengdu 610213, China; Key Laboratory of Cosmic Rays (Xizang University), Ministry of Education, Lhasa 850000, China; School of Astronomy and Space Science, Nanjing University, Nanjing 210023, China; School of Physical Science and Technology & School of Information Science and Technology, Southwest Jiaotong University, Chengdu 610031, China; School of Astronomy and Space Science, Nanjing University, Nanjing 210023, China; State Key Laboratory of Particle Astrophysics & Experimental Physics Division & Computing Center, Institute of High Energy Physics, Chinese Academy of Sciences, Beijing 100049, China; TIANFU Cosmic Ray Research Center, Chengdu 610213, China; State Key Laboratory of Particle Astrophysics & Experimental Physics Division & Computing Center, Institute of High Energy Physics, Chinese Academy of Sciences, Beijing 100049, China; School of Physical Sciences, University of Chinese Academy of Sciences, Beijing 100049, China; TIANFU Cosmic Ray Research Center, Chengdu 610213, China; Department of Physics, The Chinese University of Hong Kong, Hong Kong 999077, China; Key Laboratory of Dark Matter and Space Astronomy & Key Laboratory of Radio Astronomy, Purple Mountain Observatory, Chinese Academy of Sciences, Nanjing 210023, China; School of Physics, Hebei Normal University, Shijiazhuang 050024, China; Key Laboratory of Radio Astronomy and Technology, National Astronomical Observatories, Chinese Academy of Sciences, Beijing 100101, China; School of Physics and Astronomy (Zhuhai) & School of Physics (Guangzhou) & Sino-French Institute of Nuclear Engineering and Technology (Zhuhai), Sun Yat-sen University, Zhuhai 519000 & Guangzhou 510275, China; School of Physics and Astronomy, Yunnan University, Kunming 650091, China; State Key Laboratory of Particle Astrophysics & Experimental Physics Division & Computing Center, Institute of High Energy Physics, Chinese Academy of Sciences, Beijing 100049, China; TIANFU Cosmic Ray Research Center, Chengdu 610213, China; State Key Laboratory of Particle Detection and Electronics, Beijing 100049, China; School of Physical Sciences, University of Science and Technology of China, Hefei 230026, China; Key Laboratory of Cosmic Rays (Xizang University), Ministry of Education, Lhasa 850000, China; School of Physical Science and Technology & School of Information Science and Technology, Southwest Jiaotong University, Chengdu 610031, China; State Key Laboratory of Particle Astrophysics & Experimental Physics Division & Computing Center, Institute of High Energy Physics, Chinese Academy of Sciences, Beijing 100049, China; School of Physical Sciences, University of Chinese Academy of Sciences, Beijing 100049, China; TIANFU Cosmic Ray Research Center, Chengdu 610213, China; Key Laboratory of Dark Matter and Space Astronomy & Key Laboratory of Radio Astronomy, Purple Mountain Observatory, Chinese Academy of Sciences, Nanjing 210023, China; Center for Astrophysics, Guangzhou University, Guangzhou 510006, China; Key Laboratory of Dark Matter and Space Astronomy & Key Laboratory of Radio Astronomy, Purple Mountain Observatory, Chinese Academy of Sciences, Nanjing 210023, China; School of Physics and Astronomy, Yunnan University, Kunming 650091, China; Research Center for Astronomical Computing, Zhejiang Laboratory, Hangzhou 311121, China; State Key Laboratory of Particle Astrophysics & Experimental Physics Division & Computing Center, Institute of High Energy Physics, Chinese Academy of Sciences, Beijing 100049, China; TIANFU Cosmic Ray Research Center, Chengdu 610213, China; Institute of Frontier and Interdisciplinary Science, Shandong University, Qingdao 266237, China; State Key Laboratory of Particle Astrophysics & Experimental Physics Division & Computing Center, Institute of High Energy Physics, Chinese Academy of Sciences, Beijing 100049, China; Key Laboratory of Dark Matter and Space Astronomy & Key Laboratory of Radio Astronomy, Purple Mountain Observatory, Chinese Academy of Sciences, Nanjing 210023, China; State Key Laboratory of Particle Astrophysics & Experimental Physics Division & Computing Center, Institute of High Energy Physics, Chinese Academy of Sciences, Beijing 100049, China; TIANFU Cosmic Ray Research Center, Chengdu 610213, China; Institute of Frontier and Interdisciplinary Science, Shandong University, Qingdao 266237, China; Research Center for Astronomical Computing, Zhejiang Laboratory, Hangzhou 311121, China; Key Laboratory of Cosmic Rays (Xizang University), Ministry of Education, Lhasa 850000, China; APC, Université Paris Cité, CNRS/IN2P3, CEA/IRFU, Observatoire de Paris, Paris 119 75205, France; State Key Laboratory of Particle Astrophysics & Experimental Physics Division & Computing Center, Institute of High Energy Physics, Chinese Academy of Sciences, Beijing 100049, China; TIANFU Cosmic Ray Research Center, Chengdu 610213, China; Institute of Frontier and Interdisciplinary Science, Shandong University, Qingdao 266237, China; Key Laboratory of Cosmic Rays (Xizang University), Ministry of Education, Lhasa 850000, China; State Key Laboratory of Particle Astrophysics & Experimental Physics Division & Computing Center, Institute of High Energy Physics, Chinese Academy of Sciences, Beijing 100049, China; TIANFU Cosmic Ray Research Center, Chengdu 610213, China; State Key Laboratory of Particle Astrophysics & Experimental Physics Division & Computing Center, Institute of High Energy Physics, Chinese Academy of Sciences, Beijing 100049, China; School of Physical Sciences, University of Chinese Academy of Sciences, Beijing 100049, China; TIANFU Cosmic Ray Research Center, Chengdu 610213, China; School of Physics and Astronomy, Yunnan University, Kunming 650091, China; School of Physics and Astronomy (Zhuhai) & School of Physics (Guangzhou) & Sino-French Institute of Nuclear Engineering and Technology (Zhuhai), Sun Yat-sen University, Zhuhai 519000 & Guangzhou 510275, China; State Key Laboratory of Particle Astrophysics & Experimental Physics Division & Computing Center, Institute of High Energy Physics, Chinese Academy of Sciences, Beijing 100049, China; TIANFU Cosmic Ray Research Center, Chengdu 610213, China; Tsung-Dao Lee Institute & School of Physics and Astronomy, Shanghai Jiao Tong University, Shanghai 200240, China; Department of Engineering Physics & Department of Physics & Department of Astronomy, Tsinghua University, Beijing 100084, China; State Key Laboratory of Particle Astrophysics & Experimental Physics Division & Computing Center, Institute of High Energy Physics, Chinese Academy of Sciences, Beijing 100049, China; TIANFU Cosmic Ray Research Center, Chengdu 610213, China; State Key Laboratory of Particle Astrophysics & Experimental Physics Division & Computing Center, Institute of High Energy Physics, Chinese Academy of Sciences, Beijing 100049, China; TIANFU Cosmic Ray Research Center, Chengdu 610213, China; State Key Laboratory of Particle Detection and Electronics, Beijing 100049, China; Shanghai Astronomical Observatory, Chinese Academy of Sciences, Shanghai 200030, China; Department of Engineering Physics & Department of Physics & Department of Astronomy, Tsinghua University, Beijing 100084, China; School of Physical Science and Technology & School of Information Science and Technology, Southwest Jiaotong University, Chengdu 610031, China; State Key Laboratory of Particle Astrophysics & Experimental Physics Division & Computing Center, Institute of High Energy Physics, Chinese Academy of Sciences, Beijing 100049, China; TIANFU Cosmic Ray Research Center, Chengdu 610213, China; Key Laboratory of Dark Matter and Space Astronomy & Key Laboratory of Radio Astronomy, Purple Mountain Observatory, Chinese Academy of Sciences, Nanjing 210023, China; School of Physics and Microelectronics, Zhengzhou University, Zhengzhou 450001, China; Department of Physics, The Chinese University of Hong Kong, Hong Kong 999077, China; State Key Laboratory of Particle Astrophysics & Experimental Physics Division & Computing Center, Institute of High Energy Physics, Chinese Academy of Sciences, Beijing 100049, China; School of Physical Sciences, University of Chinese Academy of Sciences, Beijing 100049, China; TIANFU Cosmic Ray Research Center, Chengdu 610213, China; State Key Laboratory of Particle Astrophysics & Experimental Physics Division & Computing Center, Institute of High Energy Physics, Chinese Academy of Sciences, Beijing 100049, China; School of Physical Sciences, University of Chinese Academy of Sciences, Beijing 100049, China; TIANFU Cosmic Ray Research Center, Chengdu 610213, China; Key Laboratory of Dark Matter and Space Astronomy & Key Laboratory of Radio Astronomy, Purple Mountain Observatory, Chinese Academy of Sciences, Nanjing 210023, China; Key Laboratory of Dark Matter and Space Astronomy & Key Laboratory of Radio Astronomy, Purple Mountain Observatory, Chinese Academy of Sciences, Nanjing 210023, China; Key Laboratory of Dark Matter and Space Astronomy & Key Laboratory of Radio Astronomy, Purple Mountain Observatory, Chinese Academy of Sciences, Nanjing 210023, China; School of Physical Science and Technology & School of Information Science and Technology, Southwest Jiaotong University, Chengdu 610031, China; Tsung-Dao Lee Institute & School of Physics and Astronomy, Shanghai Jiao Tong University, Shanghai 200240, China; State Key Laboratory of Particle Astrophysics & Experimental Physics Division & Computing Center, Institute of High Energy Physics, Chinese Academy of Sciences, Beijing 100049, China; School of Physical Sciences, University of Chinese Academy of Sciences, Beijing 100049, China; TIANFU Cosmic Ray Research Center, Chengdu 610213, China; State Key Laboratory of Particle Astrophysics & Experimental Physics Division & Computing Center, Institute of High Energy Physics, Chinese Academy of Sciences, Beijing 100049, China; TIANFU Cosmic Ray Research Center, Chengdu 610213, China; Yunnan Observatories, Chinese Academy of Sciences, Kunming 650216, China; State Key Laboratory of Particle Astrophysics & Experimental Physics Division & Computing Center, Institute of High Energy Physics, Chinese Academy of Sciences, Beijing 100049, China; School of Physical Sciences, University of Chinese Academy of Sciences, Beijing 100049, China; TIANFU Cosmic Ray Research Center, Chengdu 610213, China; State Key Laboratory of Particle Astrophysics & Experimental Physics Division & Computing Center, Institute of High Energy Physics, Chinese Academy of Sciences, Beijing 100049, China; TIANFU Cosmic Ray Research Center, Chengdu 610213, China; China Center of Advanced Science and Technology, Beijing 100190, China; School of Astronomy and Space Science, Nanjing University, Nanjing 210023, China; School of Physical Science and Technology & School of Information Science and Technology, Southwest Jiaotong University, Chengdu 610031, China; State Key Laboratory of Particle Astrophysics & Experimental Physics Division & Computing Center, Institute of High Energy Physics, Chinese Academy of Sciences, Beijing 100049, China; School of Physical Sciences, University of Chinese Academy of Sciences, Beijing 100049, China; TIANFU Cosmic Ray Research Center, Chengdu 610213, China; State Key Laboratory of Particle Astrophysics & Experimental Physics Division & Computing Center, Institute of High Energy Physics, Chinese Academy of Sciences, Beijing 100049, China; TIANFU Cosmic Ray Research Center, Chengdu 610213, China; School of Physics and Astronomy (Zhuhai) & School of Physics (Guangzhou) & Sino-French Institute of Nuclear Engineering and Technology (Zhuhai), Sun Yat-sen University, Zhuhai 519000 & Guangzhou 510275, China; Institute of Frontier and Interdisciplinary Science, Shandong University, Qingdao 266237, China; Key Laboratory of Dark Matter and Space Astronomy & Key Laboratory of Radio Astronomy, Purple Mountain Observatory, Chinese Academy of Sciences, Nanjing 210023, China; State Key Laboratory of Particle Astrophysics & Experimental Physics Division & Computing Center, Institute of High Energy Physics, Chinese Academy of Sciences, Beijing 100049, China; TIANFU Cosmic Ray Research Center, Chengdu 610213, China; China Center of Advanced Science and Technology, Beijing 100190, China; School of Astronomy and Space Science, Nanjing University, Nanjing 210023, China; State Key Laboratory of Particle Astrophysics & Experimental Physics Division & Computing Center, Institute of High Energy Physics, Chinese Academy of Sciences, Beijing 100049, China; TIANFU Cosmic Ray Research Center, Chengdu 610213, China; State Key Laboratory of Particle Detection and Electronics, Beijing 100049, China; School of Physical Science and Technology & School of Information Science and Technology, Southwest Jiaotong University, Chengdu 610031, China; Institute of Frontier and Interdisciplinary Science, Shandong University, Qingdao 266237, China; State Key Laboratory of Particle Astrophysics & Experimental Physics Division & Computing Center, Institute of High Energy Physics, Chinese Academy of Sciences, Beijing 100049, China; TIANFU Cosmic Ray Research Center, Chengdu 610213, China; School of Physical Sciences, University of Science and Technology of China, Hefei 230026, China; State Key Laboratory of Particle Detection and Electronics, Beijing 100049, China; State Key Laboratory of Particle Astrophysics & Experimental Physics Division & Computing Center, Institute of High Energy Physics, Chinese Academy of Sciences, Beijing 100049, China; TIANFU Cosmic Ray Research Center, Chengdu 610213, China; School of Physics and Astronomy, Yunnan University, Kunming 650091, China; School of Physical Science and Technology & School of Information Science and Technology, Southwest Jiaotong University, Chengdu 610031, China; Tsung-Dao Lee Institute & School of Physics and Astronomy, Shanghai Jiao Tong University, Shanghai 200240, China; College of Physics, Sichuan University, Chengdu 610065, China; Institute for Nuclear Research of Russian Academy of Sciences, Moscow 117312, Russia; State Key Laboratory of Particle Astrophysics & Experimental Physics Division & Computing Center, Institute of High Energy Physics, Chinese Academy of Sciences, Beijing 100049, China; TIANFU Cosmic Ray Research Center, Chengdu 610213, China; Institute for Nuclear Research of Russian Academy of Sciences, Moscow 117312, Russia; Institute for Nuclear Research of Russian Academy of Sciences, Moscow 117312, Russia; School of Physics, Hebei Normal University, Shijiazhuang 050024, China; School of Physical Sciences, University of Science and Technology of China, Hefei 230026, China; State Key Laboratory of Particle Detection and Electronics, Beijing 100049, China; State Key Laboratory of Particle Astrophysics & Experimental Physics Division & Computing Center, Institute of High Energy Physics, Chinese Academy of Sciences, Beijing 100049, China; TIANFU Cosmic Ray Research Center, Chengdu 610213, China; State Key Laboratory of Particle Astrophysics & Experimental Physics Division & Computing Center, Institute of High Energy Physics, Chinese Academy of Sciences, Beijing 100049, China; School of Physical Sciences, University of Chinese Academy of Sciences, Beijing 100049, China; TIANFU Cosmic Ray Research Center, Chengdu 610213, China; State Key Laboratory of Particle Astrophysics & Experimental Physics Division & Computing Center, Institute of High Energy Physics, Chinese Academy of Sciences, Beijing 100049, China; TIANFU Cosmic Ray Research Center, Chengdu 610213, China; State Key Laboratory of Particle Detection and Electronics, Beijing 100049, China; State Key Laboratory of Particle Astrophysics & Experimental Physics Division & Computing Center, Institute of High Energy Physics, Chinese Academy of Sciences, Beijing 100049, China; School of Physical Sciences, University of Chinese Academy of Sciences, Beijing 100049, China; TIANFU Cosmic Ray Research Center, Chengdu 610213, China; State Key Laboratory of Particle Astrophysics & Experimental Physics Division & Computing Center, Institute of High Energy Physics, Chinese Academy of Sciences, Beijing 100049, China; TIANFU Cosmic Ray Research Center, Chengdu 610213, China; School of Physical Sciences, University of Science and Technology of China, Hefei 230026, China; State Key Laboratory of Particle Astrophysics & Experimental Physics Division & Computing Center, Institute of High Energy Physics, Chinese Academy of Sciences, Beijing 100049, China; TIANFU Cosmic Ray Research Center, Chengdu 610213, China; State Key Laboratory of Particle Detection and Electronics, Beijing 100049, China; State Key Laboratory of Particle Astrophysics & Experimental Physics Division & Computing Center, Institute of High Energy Physics, Chinese Academy of Sciences, Beijing 100049, China; TIANFU Cosmic Ray Research Center, Chengdu 610213, China; Center for Relativistic Astrophysics and High Energy Physics, School of Physics and Materials Science & Institute of Space Science and Technology, Nanchang University, Nanchang 330031, China; Key Laboratory of Dark Matter and Space Astronomy & Key Laboratory of Radio Astronomy, Purple Mountain Observatory, Chinese Academy of Sciences, Nanjing 210023, China; School of Physical Sciences, University of Chinese Academy of Sciences, Beijing 100049, China; Shanghai Astronomical Observatory, Chinese Academy of Sciences, Shanghai 200030, China; Tsung-Dao Lee Institute & School of Physics and Astronomy, Shanghai Jiao Tong University, Shanghai 200240, China; Tsung-Dao Lee Institute & School of Physics and Astronomy, Shanghai Jiao Tong University, Shanghai 200240, China; State Key Laboratory of Particle Astrophysics & Experimental Physics Division & Computing Center, Institute of High Energy Physics, Chinese Academy of Sciences, Beijing 100049, China; TIANFU Cosmic Ray Research Center, Chengdu 610213, China; School of Physical Sciences, University of Science and Technology of China, Hefei 230026, China; State Key Laboratory of Particle Detection and Electronics, Beijing 100049, China; Tsung-Dao Lee Institute & School of Physics and Astronomy, Shanghai Jiao Tong University, Shanghai 200240, China; State Key Laboratory of Particle Astrophysics & Experimental Physics Division & Computing Center, Institute of High Energy Physics, Chinese Academy of Sciences, Beijing 100049, China; School of Physical Sciences, University of Chinese Academy of Sciences, Beijing 100049, China; TIANFU Cosmic Ray Research Center, Chengdu 610213, China; State Key Laboratory of Particle Astrophysics & Experimental Physics Division & Computing Center, Institute of High Energy Physics, Chinese Academy of Sciences, Beijing 100049, China; TIANFU Cosmic Ray Research Center, Chengdu 610213, China; School of Physics & Kavli Institute for Astronomy and Astrophysics, Peking University, Beijing 100871, China; Guangxi Key Laboratory for Relativistic Astrophysics, School of Physical Science and Technology, Guangxi University, Nanning 530004, China; Guangxi Key Laboratory for Relativistic Astrophysics, School of Physical Science and Technology, Guangxi University, Nanning 530004, China; School of Physics and Astronomy (Zhuhai) & School of Physics (Guangzhou) & Sino-French Institute of Nuclear Engineering and Technology (Zhuhai), Sun Yat-sen University, Zhuhai 519000 & Guangzhou 510275, China; Key Laboratory of Dark Matter and Space Astronomy & Key Laboratory of Radio Astronomy, Purple Mountain Observatory, Chinese Academy of Sciences, Nanjing 210023, China; State Key Laboratory of Particle Astrophysics & Experimental Physics Division & Computing Center, Institute of High Energy Physics, Chinese Academy of Sciences, Beijing 100049, China; TIANFU Cosmic Ray Research Center, Chengdu 610213, China; Institute of Frontier and Interdisciplinary Science, Shandong University, Qingdao 266237, China; Tsung-Dao Lee Institute & School of Physics and Astronomy, Shanghai Jiao Tong University, Shanghai 200240, China; School of Physical Science and Technology & School of Information Science and Technology, Southwest Jiaotong University, Chengdu 610031, China; School of Physics and Microelectronics, Zhengzhou University, Zhengzhou 450001, China; State Key Laboratory of Particle Astrophysics & Experimental Physics Division & Computing Center, Institute of High Energy Physics, Chinese Academy of Sciences, Beijing 100049, China; TIANFU Cosmic Ray Research Center, Chengdu 610213, China; State Key Laboratory of Particle Astrophysics & Experimental Physics Division & Computing Center, Institute of High Energy Physics, Chinese Academy of Sciences, Beijing 100049, China; TIANFU Cosmic Ray Research Center, Chengdu 610213, China; School of Physical Science and Technology & School of Information Science and Technology, Southwest Jiaotong University, Chengdu 610031, China; Key Laboratory of Cosmic Rays (Xizang University), Ministry of Education, Lhasa 850000, China; School of Astronomy and Space Science, Nanjing University, Nanjing 210023, China; School of Physical Science and Technology & School of Information Science and Technology, Southwest Jiaotong University, Chengdu 610031, China; State Key Laboratory of Particle Astrophysics & Experimental Physics Division & Computing Center, Institute of High Energy Physics, Chinese Academy of Sciences, Beijing 100049, China; TIANFU Cosmic Ray Research Center, Chengdu 610213, China; School of Physical Science and Technology & School of Information Science and Technology, Southwest Jiaotong University, Chengdu 610031, China; Center for Astrophysics, Guangzhou University, Guangzhou 510006, China; School of Physical Science and Technology & School of Information Science and Technology, Southwest Jiaotong University, Chengdu 610031, China; Department of Engineering Physics & Department of Physics & Department of Astronomy, Tsinghua University, Beijing 100084, China; Department of Engineering Physics & Department of Physics & Department of Astronomy, Tsinghua University, Beijing 100084, China; School of Physics and Astronomy (Zhuhai) & School of Physics (Guangzhou) & Sino-French Institute of Nuclear Engineering and Technology (Zhuhai), Sun Yat-sen University, Zhuhai 519000 & Guangzhou 510275, China; Tsung-Dao Lee Institute & School of Physics and Astronomy, Shanghai Jiao Tong University, Shanghai 200240, China; State Key Laboratory of Particle Astrophysics & Experimental Physics Division & Computing Center, Institute of High Energy Physics, Chinese Academy of Sciences, Beijing 100049, China; TIANFU Cosmic Ray Research Center, Chengdu 610213, China; School of Physics and Microelectronics, Zhengzhou University, Zhengzhou 450001, China; School of Physics & Kavli Institute for Astronomy and Astrophysics, Peking University, Beijing 100871, China; State Key Laboratory of Particle Astrophysics & Experimental Physics Division & Computing Center, Institute of High Energy Physics, Chinese Academy of Sciences, Beijing 100049, China; TIANFU Cosmic Ray Research Center, Chengdu 610213, China; State Key Laboratory of Particle Astrophysics & Experimental Physics Division & Computing Center, Institute of High Energy Physics, Chinese Academy of Sciences, Beijing 100049, China; TIANFU Cosmic Ray Research Center, Chengdu 610213, China; Yunnan Observatories, Chinese Academy of Sciences, Kunming 650216, China; State Key Laboratory of Particle Astrophysics & Experimental Physics Division & Computing Center, Institute of High Energy Physics, Chinese Academy of Sciences, Beijing 100049, China; TIANFU Cosmic Ray Research Center, Chengdu 610213, China; Department of Physics, Faculty of Science, Mahidol University, Bangkok 10400, Thailand; School of Physics and Technology, Nanjing Normal University, Nanjing 210023, China; School of Physics and Microelectronics, Zhengzhou University, Zhengzhou 450001, China; APC, Université Paris Cité, CNRS/IN2P3, CEA/IRFU, Observatoire de Paris, Paris 119 75205, France; Department of Physics, The Chinese University of Hong Kong, Hong Kong 999077, China; Key Laboratory of Dark Matter and Space Astronomy & Key Laboratory of Radio Astronomy, Purple Mountain Observatory, Chinese Academy of Sciences, Nanjing 210023, China; School of Physical Science and Technology & School of Information Science and Technology, Southwest Jiaotong University, Chengdu 610031, China; Center for Astrophysics, Guangzhou University, Guangzhou 510006, China; Department of Physics, Faculty of Science, Mahidol University, Bangkok 10400, Thailand; Center for Astrophysics, Guangzhou University, Guangzhou 510006, China; State Key Laboratory of Particle Astrophysics & Experimental Physics Division & Computing Center, Institute of High Energy Physics, Chinese Academy of Sciences, Beijing 100049, China; School of Physical Sciences, University of Chinese Academy of Sciences, Beijing 100049, China; TIANFU Cosmic Ray Research Center, Chengdu 610213, China; State Key Laboratory of Particle Astrophysics & Experimental Physics Division & Computing Center, Institute of High Energy Physics, Chinese Academy of Sciences, Beijing 100049, China; TIANFU Cosmic Ray Research Center, Chengdu 610213, China; School of Physical Sciences, University of Science and Technology of China, Hefei 230026, China; State Key Laboratory of Particle Astrophysics & Experimental Physics Division & Computing Center, Institute of High Energy Physics, Chinese Academy of Sciences, Beijing 100049, China; School of Physical Sciences, University of Chinese Academy of Sciences, Beijing 100049, China; TIANFU Cosmic Ray Research Center, Chengdu 610213, China; Key Laboratory of Dark Matter and Space Astronomy & Key Laboratory of Radio Astronomy, Purple Mountain Observatory, Chinese Academy of Sciences, Nanjing 210023, China; Department of Physics, Faculty of Science, Mahidol University, Bangkok 10400, Thailand; Department of Physics, Faculty of Science, Mahidol University, Bangkok 10400, Thailand; APC, Université Paris Cité, CNRS/IN2P3, CEA/IRFU, Observatoire de Paris, Paris 119 75205, France; School of Physics, Hebei Normal University, Shijiazhuang 050024, China; Institute for Nuclear Research of Russian Academy of Sciences, Moscow 117312, Russia; Moscow Institute of Physics and Technology, Moscow 141700, Russia; School of Astronomy and Space Science, Nanjing University, Nanjing 210023, China; State Key Laboratory of Particle Astrophysics & Experimental Physics Division & Computing Center, Institute of High Energy Physics, Chinese Academy of Sciences, Beijing 100049, China; TIANFU Cosmic Ray Research Center, Chengdu 610213, China; School of Physical Sciences, University of Science and Technology of China, Hefei 230026, China; Center for Relativistic Astrophysics and High Energy Physics, School of Physics and Materials Science & Institute of Space Science and Technology, Nanchang University, Nanchang 330031, China; School of Physics & Kavli Institute for Astronomy and Astrophysics, Peking University, Beijing 100871, China; Institute for Nuclear Research of Russian Academy of Sciences, Moscow 117312, Russia; Moscow Institute of Physics and Technology, Moscow 141700, Russia; Institute for Nuclear Research of Russian Academy of Sciences, Moscow 117312, Russia; Key Laboratory of Dark Matter and Space Astronomy & Key Laboratory of Radio Astronomy, Purple Mountain Observatory, Chinese Academy of Sciences, Nanjing 210023, China; School of Physical Sciences, University of Science and Technology of China, Hefei 230026, China; Key Laboratory of Dark Matter and Space Astronomy & Key Laboratory of Radio Astronomy, Purple Mountain Observatory, Chinese Academy of Sciences, Nanjing 210023, China; Institute of Frontier and Interdisciplinary Science, Shandong University, Qingdao 266237, China; State Key Laboratory of Particle Astrophysics & Experimental Physics Division & Computing Center, Institute of High Energy Physics, Chinese Academy of Sciences, Beijing 100049, China; TIANFU Cosmic Ray Research Center, Chengdu 610213, China; Guangxi Key Laboratory for Relativistic Astrophysics, School of Physical Science and Technology, Guangxi University, Nanning 530004, China; National Space Science Center, Chinese Academy of Sciences, Beijing 100190, China; Institute of Frontier and Interdisciplinary Science, Shandong University, Qingdao 266237, China; School of Physics, Huazhong University of Science and Technology, Wuhan 430074, China; School of Physics and Astronomy (Zhuhai) & School of Physics (Guangzhou) & Sino-French Institute of Nuclear Engineering and Technology (Zhuhai), Sun Yat-sen University, Zhuhai 519000 & Guangzhou 510275, China; School of Astronomy and Space Science, Nanjing University, Nanjing 210023, China; Center for Relativistic Astrophysics and High Energy Physics, School of Physics and Materials Science & Institute of Space Science and Technology, Nanchang University, Nanchang 330031, China; Tsung-Dao Lee Institute & School of Physics and Astronomy, Shanghai Jiao Tong University, Shanghai 200240, China; State Key Laboratory of Particle Detection and Electronics, Beijing 100049, China; School of Physical Sciences, University of Science and Technology of China, Hefei 230026, China; School of Physical Sciences, University of Chinese Academy of Sciences, Beijing 100049, China; Key Laboratory of Radio Astronomy and Technology, National Astronomical Observatories, Chinese Academy of Sciences, Beijing 100101, China; School of Astronomy and Space Science, Nanjing University, Nanjing 210023, China; School of Physics and Astronomy (Zhuhai) & School of Physics (Guangzhou) & Sino-French Institute of Nuclear Engineering and Technology (Zhuhai), Sun Yat-sen University, Zhuhai 519000 & Guangzhou 510275, China; National Space Science Center, Chinese Academy of Sciences, Beijing 100190, China; School of Physical Sciences, University of Science and Technology of China, Hefei 230026, China; Center for Astrophysics, Guangzhou University, Guangzhou 510006, China; Yunnan Observatories, Chinese Academy of Sciences, Kunming 650216, China; School of Physics & Kavli Institute for Astronomy and Astrophysics, Peking University, Beijing 100871, China; School of Astronomy and Space Science, Nanjing University, Nanjing 210023, China; School of Physics, Huazhong University of Science and Technology, Wuhan 430074, China; State Key Laboratory of Particle Astrophysics & Experimental Physics Division & Computing Center, Institute of High Energy Physics, Chinese Academy of Sciences, Beijing 100049, China; School of Physical Sciences, University of Chinese Academy of Sciences, Beijing 100049, China; TIANFU Cosmic Ray Research Center, Chengdu 610213, China; State Key Laboratory of Particle Astrophysics & Experimental Physics Division & Computing Center, Institute of High Energy Physics, Chinese Academy of Sciences, Beijing 100049, China; TIANFU Cosmic Ray Research Center, Chengdu 610213, China; School of Physics, Hebei Normal University, Shijiazhuang 050024, China; Institute of Frontier and Interdisciplinary Science, Shandong University, Qingdao 266237, China; School of Physics and Astronomy (Zhuhai) & School of Physics (Guangzhou) & Sino-French Institute of Nuclear Engineering and Technology (Zhuhai), Sun Yat-sen University, Zhuhai 519000 & Guangzhou 510275, China; Guangxi Key Laboratory for Relativistic Astrophysics, School of Physical Science and Technology, Guangxi University, Nanning 530004, China; School of Physical Science and Technology & School of Information Science and Technology, Southwest Jiaotong University, Chengdu 610031, China; School of Astronomy and Space Science, Nanjing University, Nanjing 210023, China; School of Physical Science and Technology & School of Information Science and Technology, Southwest Jiaotong University, Chengdu 610031, China; State Key Laboratory of Particle Astrophysics & Experimental Physics Division & Computing Center, Institute of High Energy Physics, Chinese Academy of Sciences, Beijing 100049, China; TIANFU Cosmic Ray Research Center, Chengdu 610213, China; College of Physics, Sichuan University, Chengdu 610065, China; School of Physics and Astronomy, Yunnan University, Kunming 650091, China; State Key Laboratory of Particle Astrophysics & Experimental Physics Division & Computing Center, Institute of High Energy Physics, Chinese Academy of Sciences, Beijing 100049, China; TIANFU Cosmic Ray Research Center, Chengdu 610213, China; State Key Laboratory of Particle Detection and Electronics, Beijing 100049, China; Key Laboratory of Dark Matter and Space Astronomy & Key Laboratory of Radio Astronomy, Purple Mountain Observatory, Chinese Academy of Sciences, Nanjing 210023, China; Key Laboratory of Dark Matter and Space Astronomy & Key Laboratory of Radio Astronomy, Purple Mountain Observatory, Chinese Academy of Sciences, Nanjing 210023, China; State Key Laboratory of Particle Astrophysics & Experimental Physics Division & Computing Center, Institute of High Energy Physics, Chinese Academy of Sciences, Beijing 100049, China; School of Physical Sciences, University of Chinese Academy of Sciences, Beijing 100049, China; TIANFU Cosmic Ray Research Center, Chengdu 610213, China; State Key Laboratory of Particle Astrophysics & Experimental Physics Division & Computing Center, Institute of High Energy Physics, Chinese Academy of Sciences, Beijing 100049, China; TIANFU Cosmic Ray Research Center, Chengdu 610213, China; School of Physics and Technology, Nanjing Normal University, Nanjing 210023, China; State Key Laboratory of Particle Astrophysics & Experimental Physics Division & Computing Center, Institute of High Energy Physics, Chinese Academy of Sciences, Beijing 100049, China; TIANFU Cosmic Ray Research Center, Chengdu 610213, China; State Key Laboratory of Particle Astrophysics & Experimental Physics Division & Computing Center, Institute of High Energy Physics, Chinese Academy of Sciences, Beijing 100049, China; TIANFU Cosmic Ray Research Center, Chengdu 610213, China; School of Physics, Huazhong University of Science and Technology, Wuhan 430074, China; State Key Laboratory of Particle Astrophysics & Experimental Physics Division & Computing Center, Institute of High Energy Physics, Chinese Academy of Sciences, Beijing 100049, China; TIANFU Cosmic Ray Research Center, Chengdu 610213, China; Key Laboratory of Dark Matter and Space Astronomy & Key Laboratory of Radio Astronomy, Purple Mountain Observatory, Chinese Academy of Sciences, Nanjing 210023, China; School of Physical Sciences, University of Science and Technology of China, Hefei 230026, China; State Key Laboratory of Particle Astrophysics & Experimental Physics Division & Computing Center, Institute of High Energy Physics, Chinese Academy of Sciences, Beijing 100049, China; TIANFU Cosmic Ray Research Center, Chengdu 610213, China; School of Physical Sciences, University of Science and Technology of China, Hefei 230026, China; Key Laboratory of Dark Matter and Space Astronomy & Key Laboratory of Radio Astronomy, Purple Mountain Observatory, Chinese Academy of Sciences, Nanjing 210023, China; School of Physical Science and Technology & School of Information Science and Technology, Southwest Jiaotong University, Chengdu 610031, China; School of Physical Sciences, University of Chinese Academy of Sciences, Beijing 100049, China; Shanghai Astronomical Observatory, Chinese Academy of Sciences, Shanghai 200030, China; School of Physics, Hebei Normal University, Shijiazhuang 050024, China; State Key Laboratory of Particle Astrophysics & Experimental Physics Division & Computing Center, Institute of High Energy Physics, Chinese Academy of Sciences, Beijing 100049, China; TIANFU Cosmic Ray Research Center, Chengdu 610213, China; School of Physical Science and Technology & School of Information Science and Technology, Southwest Jiaotong University, Chengdu 610031, China; Shanghai Astronomical Observatory, Chinese Academy of Sciences, Shanghai 200030, China; Yunnan Observatories, Chinese Academy of Sciences, Kunming 650216, China; State Key Laboratory of Particle Astrophysics & Experimental Physics Division & Computing Center, Institute of High Energy Physics, Chinese Academy of Sciences, Beijing 100049, China; School of Physical Sciences, University of Chinese Academy of Sciences, Beijing 100049, China; TIANFU Cosmic Ray Research Center, Chengdu 610213, China; Tsung-Dao Lee Institute & School of Physics and Astronomy, Shanghai Jiao Tong University, Shanghai 200240, China; State Key Laboratory of Particle Astrophysics & Experimental Physics Division & Computing Center, Institute of High Energy Physics, Chinese Academy of Sciences, Beijing 100049, China; School of Physical Sciences, University of Chinese Academy of Sciences, Beijing 100049, China; TIANFU Cosmic Ray Research Center, Chengdu 610213, China; School of Physics & Kavli Institute for Astronomy and Astrophysics, Peking University, Beijing 100871, China; College of Physics, Sichuan University, Chengdu 610065, China; Institute of Frontier and Interdisciplinary Science, Shandong University, Qingdao 266237, China; School of Physics and Astronomy, Yunnan University, Kunming 650091, China; Key Laboratory of Dark Matter and Space Astronomy & Key Laboratory of Radio Astronomy, Purple Mountain Observatory, Chinese Academy of Sciences, Nanjing 210023, China; State Key Laboratory of Particle Astrophysics & Experimental Physics Division & Computing Center, Institute of High Energy Physics, Chinese Academy of Sciences, Beijing 100049, China; TIANFU Cosmic Ray Research Center, Chengdu 610213, China; College of Physics, Sichuan University, Chengdu 610065, China; Yunnan Observatories, Chinese Academy of Sciences, Kunming 650216, China; State Key Laboratory of Particle Astrophysics & Experimental Physics Division & Computing Center, Institute of High Energy Physics, Chinese Academy of Sciences, Beijing 100049, China; TIANFU Cosmic Ray Research Center, Chengdu 610213, China; State Key Laboratory of Particle Detection and Electronics, Beijing 100049, China; School of Physics and Astronomy (Zhuhai) & School of Physics (Guangzhou) & Sino-French Institute of Nuclear Engineering and Technology (Zhuhai), Sun Yat-sen University, Zhuhai 519000 & Guangzhou 510275, China; State Key Laboratory of Particle Astrophysics & Experimental Physics Division & Computing Center, Institute of High Energy Physics, Chinese Academy of Sciences, Beijing 100049, China; TIANFU Cosmic Ray Research Center, Chengdu 610213, China; School of Physical Sciences, University of Science and Technology of China, Hefei 230026, China; Center for Astrophysics, Guangzhou University, Guangzhou 510006, China; Tsung-Dao Lee Institute & School of Physics and Astronomy, Shanghai Jiao Tong University, Shanghai 200240, China; State Key Laboratory of Particle Astrophysics & Experimental Physics Division & Computing Center, Institute of High Energy Physics, Chinese Academy of Sciences, Beijing 100049, China; TIANFU Cosmic Ray Research Center, Chengdu 610213, China; Key Laboratory of Dark Matter and Space Astronomy & Key Laboratory of Radio Astronomy, Purple Mountain Observatory, Chinese Academy of Sciences, Nanjing 210023, China; State Key Laboratory of Particle Astrophysics & Experimental Physics Division & Computing Center, Institute of High Energy Physics, Chinese Academy of Sciences, Beijing 100049, China; TIANFU Cosmic Ray Research Center, Chengdu 610213, China; Institute of Frontier and Interdisciplinary Science, Shandong University, Qingdao 266237, China; State Key Laboratory of Particle Astrophysics & Experimental Physics Division & Computing Center, Institute of High Energy Physics, Chinese Academy of Sciences, Beijing 100049, China; TIANFU Cosmic Ray Research Center, Chengdu 610213, China; State Key Laboratory of Particle Astrophysics & Experimental Physics Division & Computing Center, Institute of High Energy Physics, Chinese Academy of Sciences, Beijing 100049, China; TIANFU Cosmic Ray Research Center, Chengdu 610213, China; School of Physical Sciences, University of Science and Technology of China, Hefei 230026, China; Key Laboratory of Dark Matter and Space Astronomy & Key Laboratory of Radio Astronomy, Purple Mountain Observatory, Chinese Academy of Sciences, Nanjing 210023, China; State Key Laboratory of Particle Astrophysics & Experimental Physics Division & Computing Center, Institute of High Energy Physics, Chinese Academy of Sciences, Beijing 100049, China; School of Physical Sciences, University of Chinese Academy of Sciences, Beijing 100049, China; TIANFU Cosmic Ray Research Center, Chengdu 610213, China; Key Laboratory of Dark Matter and Space Astronomy & Key Laboratory of Radio Astronomy, Purple Mountain Observatory, Chinese Academy of Sciences, Nanjing 210023, China; State Key Laboratory of Particle Astrophysics & Experimental Physics Division & Computing Center, Institute of High Energy Physics, Chinese Academy of Sciences, Beijing 100049, China; TIANFU Cosmic Ray Research Center, Chengdu 610213, China; State Key Laboratory of Particle Detection and Electronics, Beijing 100049, China; School of Physics and Astronomy, Yunnan University, Kunming 650091, China; School of Physics and Astronomy (Zhuhai) & School of Physics (Guangzhou) & Sino-French Institute of Nuclear Engineering and Technology (Zhuhai), Sun Yat-sen University, Zhuhai 519000 & Guangzhou 510275, China; State Key Laboratory of Particle Astrophysics & Experimental Physics Division & Computing Center, Institute of High Energy Physics, Chinese Academy of Sciences, Beijing 100049, China; TIANFU Cosmic Ray Research Center, Chengdu 610213, China; School of Astronomy and Space Science, Nanjing University, Nanjing 210023, China; State Key Laboratory of Particle Astrophysics & Experimental Physics Division & Computing Center, Institute of High Energy Physics, Chinese Academy of Sciences, Beijing 100049, China; TIANFU Cosmic Ray Research Center, Chengdu 610213, China; School of Astronomy and Space Science, Nanjing University, Nanjing 210023, China; School of Physical Science and Technology & School of Information Science and Technology, Southwest Jiaotong University, Chengdu 610031, China; Tsung-Dao Lee Institute & School of Physics and Astronomy, Shanghai Jiao Tong University, Shanghai 200240, China; Guangxi Key Laboratory for Relativistic Astrophysics, School of Physical Science and Technology, Guangxi University, Nanning 530004, China; School of Physics and Astronomy, Yunnan University, Kunming 650091, China; Key Laboratory of Radio Astronomy and Technology, National Astronomical Observatories, Chinese Academy of Sciences, Beijing 100101, China; School of Physics and Astronomy, Yunnan University, Kunming 650091, China; School of Physics and Astronomy, Yunnan University, Kunming 650091, China; School of Physical Sciences, University of Science and Technology of China, Hefei 230026, China; Key Laboratory of Dark Matter and Space Astronomy & Key Laboratory of Radio Astronomy, Purple Mountain Observatory, Chinese Academy of Sciences, Nanjing 210023, China; Key Laboratory of Dark Matter and Space Astronomy & Key Laboratory of Radio Astronomy, Purple Mountain Observatory, Chinese Academy of Sciences, Nanjing 210023, China; School of Physics, Hebei Normal University, Shijiazhuang 050024, China; State Key Laboratory of Particle Astrophysics & Experimental Physics Division & Computing Center, Institute of High Energy Physics, Chinese Academy of Sciences, Beijing 100049, China; TIANFU Cosmic Ray Research Center, Chengdu 610213, China; School of Physics, Hebei Normal University, Shijiazhuang 050024, China; School of Physics and Technology, Nanjing Normal University, Nanjing 210023, China; State Key Laboratory of Particle Astrophysics & Experimental Physics Division & Computing Center, Institute of High Energy Physics, Chinese Academy of Sciences, Beijing 100049, China; TIANFU Cosmic Ray Research Center, Chengdu 610213, China; State Key Laboratory of Particle Astrophysics & Experimental Physics Division & Computing Center, Institute of High Energy Physics, Chinese Academy of Sciences, Beijing 100049, China; Key Laboratory of Dark Matter and Space Astronomy & Key Laboratory of Radio Astronomy, Purple Mountain Observatory, Chinese Academy of Sciences, Nanjing 210023, China; State Key Laboratory of Particle Astrophysics & Experimental Physics Division & Computing Center, Institute of High Energy Physics, Chinese Academy of Sciences, Beijing 100049, China; TIANFU Cosmic Ray Research Center, Chengdu 610213, China; School of Physical Sciences, University of Science and Technology of China, Hefei 230026, China; State Key Laboratory of Particle Astrophysics & Experimental Physics Division & Computing Center, Institute of High Energy Physics, Chinese Academy of Sciences, Beijing 100049, China; TIANFU Cosmic Ray Research Center, Chengdu 610213, China; School of Physical Sciences, University of Science and Technology of China, Hefei 230026, China; State Key Laboratory of Particle Detection and Electronics, Beijing 100049, China; School of Physics, Hebei Normal University, Shijiazhuang 050024, China; Key Laboratory of Dark Matter and Space Astronomy & Key Laboratory of Radio Astronomy, Purple Mountain Observatory, Chinese Academy of Sciences, Nanjing 210023, China; Yunnan Observatories, Chinese Academy of Sciences, Kunming 650216, China; School of Physical Sciences, University of Science and Technology of China, Hefei 230026, China; National Space Science Center, Chinese Academy of Sciences, Beijing 100190, China; School of Astronomy and Space Science, Nanjing University, Nanjing 210023, China; State Key Laboratory of Particle Astrophysics & Experimental Physics Division & Computing Center, Institute of High Energy Physics, Chinese Academy of Sciences, Beijing 100049, China; TIANFU Cosmic Ray Research Center, Chengdu 610213, China; Tsung-Dao Lee Institute & School of Physics and Astronomy, Shanghai Jiao Tong University, Shanghai 200240, China; Shanghai Astronomical Observatory, Chinese Academy of Sciences, Shanghai 200030, China; Center for Relativistic Astrophysics and High Energy Physics, School of Physics and Materials Science & Institute of Space Science and Technology, Nanchang University, Nanchang 330031, China; School of Astronomy and Space Science, Nanjing University, Nanjing 210023, China; College of Physics, Sichuan University, Chengdu 610065, China; State Key Laboratory of Particle Astrophysics & Experimental Physics Division & Computing Center, Institute of High Energy Physics, Chinese Academy of Sciences, Beijing 100049, China; School of Physical Sciences, University of Chinese Academy of Sciences, Beijing 100049, China; TIANFU Cosmic Ray Research Center, Chengdu 610213, China; School of Physical Science and Technology & School of Information Science and Technology, Southwest Jiaotong University, Chengdu 610031, China; School of Physical Sciences, University of Science and Technology of China, Hefei 230026, China; Key Laboratory of Dark Matter and Space Astronomy & Key Laboratory of Radio Astronomy, Purple Mountain Observatory, Chinese Academy of Sciences, Nanjing 210023, China; Institute of Frontier and Interdisciplinary Science, Shandong University, Qingdao 266237, China; School of Physical Science and Technology & School of Information Science and Technology, Southwest Jiaotong University, Chengdu 610031, China; Key Laboratory of Radio Astronomy and Technology, National Astronomical Observatories, Chinese Academy of Sciences, Beijing 100101, China; State Key Laboratory of Particle Astrophysics & Experimental Physics Division & Computing Center, Institute of High Energy Physics, Chinese Academy of Sciences, Beijing 100049, China; School of Physical Sciences, University of Chinese Academy of Sciences, Beijing 100049, China; TIANFU Cosmic Ray Research Center, Chengdu 610213, China; State Key Laboratory of Particle Detection and Electronics, Beijing 100049, China; School of Physics, Huazhong University of Science and Technology, Wuhan 430074, China; State Key Laboratory of Particle Astrophysics & Experimental Physics Division & Computing Center, Institute of High Energy Physics, Chinese Academy of Sciences, Beijing 100049, China; TIANFU Cosmic Ray Research Center, Chengdu 610213, China

**Keywords:** gamma ray, nonthermal radiation, cosmic ray, microquasar

## Abstract

Black holes (BHs), one of the most intriguing objects in the universe, can manifest themselves through electromagnetic radiation initiated by the accretion flow. Some stellar-mass BHs drive relativistic jets when accreting matter from their companion stars, forming microquasars. Non-thermal emission from the radio to teraelectronvolt gamma-ray band has been observed from microquasars, indicating the acceleration of relativistic particles. Here we report detection of four microquasars (SS 433, V4641 Sgr, GRS 1915+105, MAXI J1820+070) of spectra extending to the ultrahigh-energy (UHE; photon energy $E>100$ TeV) band, and one microquasar (Cygnus X-1) with a spectrum approaching 100 TeV, using the Large High Altitude Air Shower Observatory. Notably, the total emission associated with SS 433 cannot be interpreted with a single leptonic component. In the UHE band, its emission is in spatial coincidence with a giant atomic cloud, which is consistent with a hadronic origin. An elongated source is discovered from V4641 Sgr with the spectrum continuing up to 800 TeV. The detection of UHE gamma rays demonstrates that accreting BHs and their environments can operate as extremely efficient accelerators of particles up to 1 PeV, suggesting that microquasars are important contributors to Galactic cosmic rays, especially around the ‘knee’ region.

## Teaser

LHAASO’s detection of microquasars emitting gamma rays at unprecedented energies reveals that these black-hole jet systems are powerful particle accelerators and a likely source of our Galaxy’s PeV cosmic rays.

## INTRODUCTION

The accretion process of black holes (BHs) may lead to various astrophysical phenomena over a wide range of radiation bands by converting the gravitational energy of falling matter or rotational energy of BHs into energetic particles. The presence of powerful jets is closely correlated with the accretion state and is often an indication of a high accretion rate and efficient extraction of the rotational energy of BHs [1,2]. Accreting BHs have long been suggested as efficient accelerators of particles exceeding 100 TeV [3–5], but it is not clear how much they contribute to the measured cosmic ray (CR) flux. Gamma-ray emission is widely considered an effective probe of energetic particle acceleration processes [6]. While ultrahigh-energy (UHE) photons from the activity of accreting supermassive BHs in the distant universe (i.e. active galactic nuclei) cannot be detected due to severe absorption by the extragalactic background light and the cosmic microwave background (CMB) permeating the universe, Galactic microquasars [7,8] provide an opportunity to examine the acceleration of high-energy particles in BH-jet systems.

Among dozens of BH-jet systems identified in the Milky Way thus far, only a few of them have been detected in high-energy (HE; energy above 0.1 GeV) and very-high-energy (VHE; energy above 0.1 TeV) gamma-ray bands: previous observations detected sporadic HE gamma-ray emission [9–11] and hints of hour-scale teraelectronvolt flares [12] from Cygnus X-1; individual HE flares were reported from V404 Cygni [13–15], although the validity of these results was challenged by a later independent analysis [16]; a hint of a VHE flare was reported from GRS 1915+105 [17] and a possible gigaelectronvolt gamma-ray counterpart of the microquasar was discovered recently [18]. So far, two microquasars, SS 433 [19–22] and V4641 Sgr [23], have been unambiguously detected up to several tens of teraelectronvolts and 200 TeV, respectively, implying acceleration of ${\sim }100$-TeV and ${\sim }1$-PeV particles.

## LARGE HIGH ALTITUDE AIR SHOWER OBSERVATORY OBSERVATIONS

The Large High Altitude Air Shower Observatory (LHAASO) is a square-kilometre-scale instrument for the gamma-ray detection over a wide energy range, from 1 TeV to a few petaelectronvolts. As of now, LHAASO has reached an exceptional sensitivity of $10^{-14}\, \rm erg\, cm^{-2}s^{-1}$ at approximately 100-TeV photon energy for point-like sources, making it an ideal detector for UHE gamma-ray sources that probe accelerators of CRs around and beyond petaelectronvolt energies. In the field of view of LHAASO, there are 10 transient microquasars with dynamic evidence for BHs as central engines [24] (see Table 1). For persistent microquasars, Cyg X-1 is confirmed to host a BH as its compact object [25], and SS 433 is very likely to host a BH [26] and is therefore included in this paper. Cyg X-3 is another good BH candidate [27], but will be reported in a dedicated LHAASO study. Since these objects are located at a distance of no more than 10 kpc from Earth, where the absorption of UHE photons is not very strong, LHAASO is able to carry out detailed studies.

**Table 1.** LHAASO’s measurement of galactic BH-jet systems in the field of view.

	Distance	LHAASO	Significance		Energy range		Flux^b^
Microquasar	(kpc)	Source	($\sigma$)	Photon index	(TeV)	Extension^a^	(Crab unit)
SS 433 E.	$4.9\pm 0.4$ [31]	J1913+0455	9.9^c^	$2.82\pm 0.16$	25-100	$0.73^\circ \pm 0.07^\circ$	0.10
SS 433 W.		J1910+0509	6.3^c^	$2.94\pm 0.38$	25–100		0.082
SS 433 central		J1911+0510	8.0	$3.96\pm 0.25$	100–630	$0.32^\circ \pm 0.04^\circ$	0.32
V4641 Sgr	$6.2\pm 0.7$ [32]	J1819-2541	10.5	$2.84\pm 0.17$	40–1000	$0.33^\circ \pm 0.08^\circ$	2.6
GRS 1915+105	$9.4\pm 0.6$ [33]	J1915+1053	15.1	$2.68\pm 0.13$	25–1000	$0.28^\circ \pm 0.05^\circ$	0.11
MAXI J1820+070	$2.96\pm 0.33$ [34]	J1821+0723	6.0	$3.25\pm 0.26$	25–400	$<0.28^\circ$	0.02
Cygnus X-1	$2.2\pm 0.2$ [25]	J1958+3522	4.4	$3.98\pm 0.40$	25–100	$<0.22^\circ$	< 0.01
XTE J1859+226	$4.2\pm 0.5$ [35]	–	2.7	–	–	–	< 0.02
GS 2000+251	$2.7\pm 0.7$ [36]	–	2.3	–	–	–	< 0.04
Swift J1727.8-1613	$2.7\pm 0.3$ [37]	–	0.7	–	–	–	< 0.04
GRO J0422+32	$2.49\pm 0.3$ [38]	–	0.7	–	–	–	< 0.01
V404 Cygni	$2.39\pm 0.14$ [39]	–	1.5	–	–	–	< 0.03
XTE J1118+480	$1.7\pm 0.1$ [40]	–	0.4	–	–	–	< 0.02
V616 Mon	$1.06\pm 0.1$ [41]	–	0.4	–	–	–	< 0.01

^a^Separation between two point-like sources of SS 433 below 100 TeV; $39\%$ containment radius for SS 433 central, V4641 Sgr and GRS 1915+105; one-tailed $95\%$ confidence upper limit for the source size for MAXI J1820+070 and Cygnus X-1. ^b^At 100 TeV, $1\, {\rm Crab\sim unit}\simeq 10^{-12}\, \rm erg\, cm^{-2}s^{-1}$. ^c^The combined detection significance for the two point-like sources is $12.3\sigma $.

**Figure 1.**
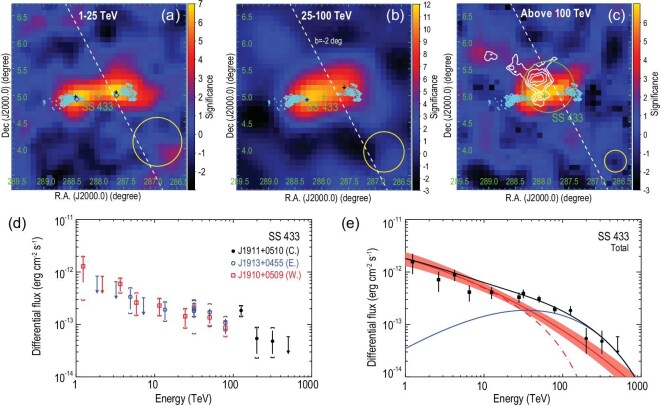
Significance maps and spectral energy distribution of SS 433 measured by LHAASO, with surrounding sources being subtracted. (a–c) SS 433 at energies of 1–25 TeV, 25–100 TeV and above 100 TeV. In the top three panels, the green cross marks the position of the BH of SS 433. In (a), the blue diamonds show the position of H.E.S.S.-detected gamma-ray emission above 10 TeV [22]. In (a) and (b), black crosses indicate the position of two resolved point-like sources at 1–25 TeV and 25–100 TeV. In (c), the white contour indicates the H i atomic clouds at a distance consistent with SS 433 (see the online supplementary material). The cyan contour shows the X-ray emission of the two lobes. The green circle in (c) exhibits 68% containment radii of the LHAASO source. The dashed white line indicates the direction of the galactic plane with $b=-2\, $deg. The yellow circles show the corresponding 68% containment radii of LHAASO PSF at the corresponding energy range. Panel (d) shows spectra of two point-like sources associated with the east and west lobes of SS 433, indicated by blue circles and red squares, respectively. The spectrum of the central extended source is shown with black dots. Panel (e) compares the total measured spectrum (with the fluxes associated with the two lobes summed) to the predictions of the models. The red solid curve indicates the best-fit spectrum based on multiwavelength data with a single leptonic component (see the online supplementary material for details), where the red band represents the $1\sigma$ uncertainty. The best-fit value of the high-energy spectral cutoff energy is about $E_{\rm e, max}=10$ PeV. The red dashed curve shows the predicted spectrum with a conservative $E_{\rm e,max}=200$ TeV, with the other parameters unchanged. The target photon fields of IC radiation include the cosmic microwave background and interstellar radiation [28]. The solid blue curve shows an additional hadronic component and the solid black curve is the sum of the hadronic component and leptonic component with $E_{\rm e, max}=200$ TeV. In (d) and (e), error bars represent the $1\sigma$ uncertainties of fluxes and bars with downward-pointing triangles are one-tailed 95% upper limits of the flux. In (d), the vertical brackets indicate the $1\sigma$ uncertainties of the flux, including the systematic errors.

**Figure 2.**
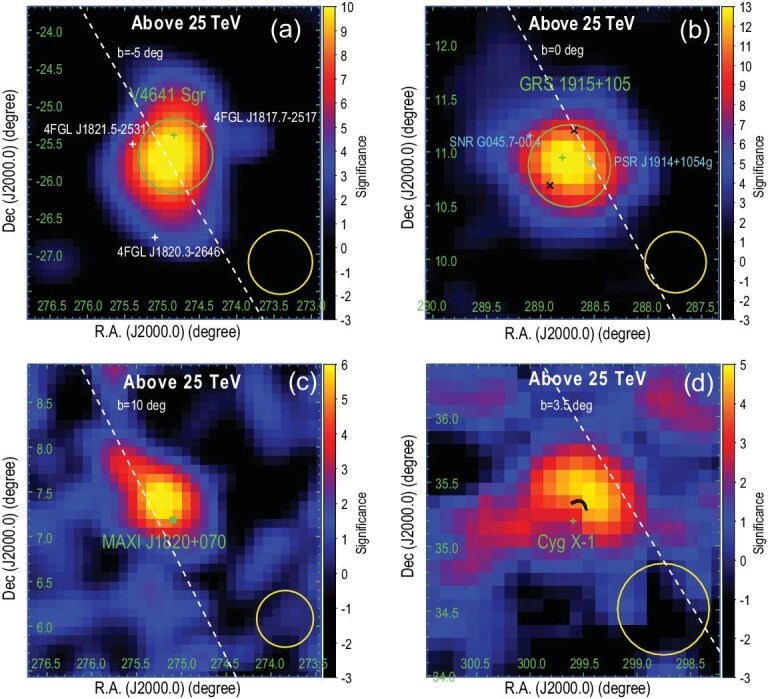
Significance maps of four other LHAASO-measured microquasars besides SS 433: (a) V4641 Sgr, (b) GRS 1915+195, (c) MAXI J1820+070 and (d) Cygnus X-1 at energies above 25 TeV, with surrounding sources being subtracted. In each panel, the green cross marks the position of the BH of each microquasar. The green circles in (a) and (b) exhibit 68% containment radii of the LHAASO sources, whereas no green circles are shown in (c) and (d) because of the point-like nature of the associated LHAASO sources. In (a), Fermi-LAT 4FGL-DR4 gigaelectronvolt gamma-ray sources within the 3$\sigma$ significance region of V4641 Sgr are shown with white crosses. In (b), other possible counterparts to the observed teraelectronvolt emission are shown with cyan crosses. The black cross represents the hot spots observed by ALMA. The black arc in (d) represents the bow-like radio structure inflated by the jet of Cygnus X-1 [30]. The yellow circle in each panel shows the corresponding 68% containment radii of LHAASO PSF. The dashed white lines indicate the direction of the galactic plane.

**Figure 3.**
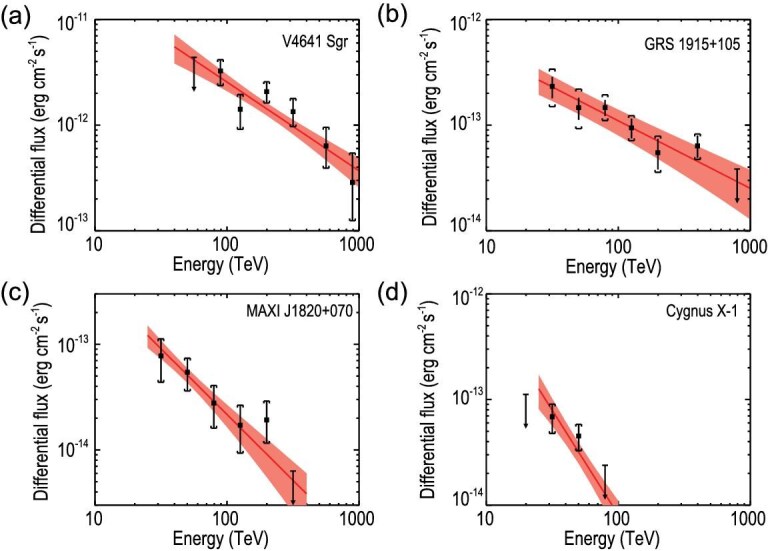
Spectra of the LHAASO sources associated with four microquasars: (a) V4641 Sgr, (b) GRS 1915+105, (c) MAXI J1820+070 and (d) Cygnus X-1. In each panel, error bars represent the $1\sigma$ statistical uncertainties of measured fluxes, and bars with downward-pointing triangles (if present) are one-tailed 95% upper limits of the flux. Vertical brackets indicate the $1\sigma$ uncertainties of fluxes, including the systematic errors. Red solid lines represent the best-fit spectrum with a power-law function, and shaded regions indicate the uncertainties.

Using the latest LHAASO dataset of photons above 25 TeV, we detect five sources at significance levels of $13.5\sigma$, $10.5\sigma$, $15.1\sigma$, $6.0\sigma$ and $4.4\sigma$, respectively, associated with SS 433, V4641 Sgr, GRS 1915+105, MAXI J1820+070 and Cygnus X-1. The data analysis follows the LHAASO standard pipelines, using events with a zenith angle of less than $50^{\circ }$, as presented in [29], while events with a zenith angle between 50$^{\circ }$ and 60$^{\circ }$ were specifically used for V4641 Sgr (see the online supplementary material for further details). Except for Cygnus X-1, the maximum photon energies of all these sources well exceed 100 TeV. This implies that BH-jet systems can efficiently accelerate particles. No gamma-ray source significant over $3\sigma$ was identified from the other seven microquasars. Our observational results, with all uncertainties reported in the main text being statistical errors and the systematic uncertainties being subdominant (see the online supplementary material for discussion) are summarized in Table 1. The significance map and the spectrum of SS 433 are shown in Fig. 1, while those of the other four detected sources are shown in Figs 2 and 3.

In a BH-jet system, there are several potential sites where particles can be accelerated. Termination shocks, which arise from interactions between jets and the surrounding medium, are promising particle accelerators, as shown by the nonthermal radiation observed from the lobes of SS 433 [20,22,42]. Particle acceleration may also take place within the jet’s outer layer of stratified velocity [43] or inside the jet through internal collisions and magnetic reconnection events [6]. In addition to the jet, powerful sub-relativistic wind launched from the accretion disk and the fast-rotating magnetosphere of the BH offer alternative possibilities for particle acceleration [44–46].

*SS 433.* A pair of jets is launched from the central BH of SS 433. The two jets are nearly perpendicularly to our line of sight and terminated approximately 40 parsecs away from the BH. We identified two point-like sources, LHAASO J1913+0455 (${\rm RA}=288.28^\circ \pm 0.04^\circ$, ${\rm DEC}=4.92^\circ \pm 0.04^\circ$) and LHAASO 1910+0509 (${\rm RA}=287.58^\circ \pm 0.06^\circ$, ${\rm DEC}=5.15^\circ \pm 0.05^\circ$), in the 25–100 TeV range. In the 1–25 TeV range, we also identified two point-like sources at similar positions to those in the 25–100 TeV range. The positions of these sources are associated with the east and west X-ray lobes, respectively, and are consistent with measurements from H.E.S.S. and HAWC [20,22]. Above 100 TeV, we identified an extended source, LHAASO J1911+0510 (${\rm RA}=287.89^\circ \pm 0.07^\circ$, ${\rm DEC}= 5.16^\circ \pm 0.07^\circ$), located $0.20^\circ$ northwest of the BH’s position. The source morphology is best described with a two-dimensional (2D) Gaussian template with $r_{39}=0.32^\circ \pm 0.04^\circ$, covering the central BH. The energy-dependent morphology of SS 433 suggests a different origin for the emission above 100 TeV compared to that below 25 TeV, or at least an additional contribution from particles other than those responsible for two lobes detected at energies below tens of teraelectronvolts. Notably, the source coincides spatially with a giant atomic cloud of $1.0\times 10^5\, M_\odot$ (see the online supplementary material), as shown by the white contours in Fig. 1c. This spatial coincidence may suggest a hadronic origin of the UHE emission from SS 433, via the proton-proton collision between hydrogen nuclei in the cloud and high-energy protons arriving at the cloud. Indeed, we found indications of multiple components in the emission above 100 TeV, consisting of two point-like sources associated with the lobes and one extended source associated with the atomic cloud (see the online supplementary material). We performed a phenomenological fitting to the multiwavelength fluxes of the two point-like sources with a one-zone leptonic model, assuming a constant injection luminosity of relativistic electrons. The influence of radiative cooling on the electron spectrum, which leads to a softening at high energies, is taken into account. As shown in Fig. 1e, our data up to 30 TeV can be well explained by the IC radiation of electrons, consistent with the suggestion of the H.E.S.S. experiment. However, the data around 100 TeV cannot be reproduced even with an extremely high (and probably unrealistic) spectral cutoff energy $E_{\rm e, max}\approx 10$ PeV, which is the best-fit value from the fitting, due to the suppression by the Klein–Nishina (KN) effect. Using the most conservative value of $E_{\rm e, max}=200$ TeV, as suggested by H.E.S.S. measurements, would entail a more significant contribution from an additional spectral component. Although a secondary leptonic component remains possible, we focus here on a hadronic scenario motivated by the proximity of the nearby atomic cloud. In this framework, we assume that an additional population of protons is injected into the surrounding medium by the microquasar, diffuses over larger distances and produces high-energy photons upon interacting with the cloud. We found that the data can be satisfactorily explained within this framework. The model suggests that SS 433 is continuously injecting petaelectronvolt protons at a power of ${\sim } 10^{38}$ erg s$^{-1}$ into the surrounding medium (see the online supplementary material for further details). These energetic protons could be produced either close to the BH at the centre of the system or at the two lobes (i.e. jet terminations). Deeper observations in the UHE gamma-ray band, as well as those in the X-ray and radio bands, are crucial for unraveling the origin of the additional spectral component.

*V4641 Sgr.* An extended source, LHAASO J1819-2541, was discovered in the vicinity of V4641 Sgr. The centre of the source (${\rm RA}=274.82^\circ \pm 0.09^\circ$, ${\rm DEC}=-25.60^\circ \pm 0.11^\circ$) is offset from V4641 Sgr by about $0.19^\circ$. There are no other reasonable astrophysical counterparts with better spatial association to the LHAASO source, making V4641 Sgr the best candidate origin of LHAASO J1819-2541. The measured spectrum can be described by a power-law function with an index of $-2.84\pm 0.17$, which is softer than the spectrum obtained by HAWC within a lower energy range of 10–200 TeV [23] (see Fig. S12). The highest photon energy detected from this source extends up to 0.8 PeV in LHAASO’s observations. IC radiation from electrons cannot reproduce such a hard spectrum extending to almost 1 PeV due to the KN effect, unless a very hard injection spectrum is assumed or the IC radiation dominates the cooling even in the KN regime (which would require a very weak magnetic field of $B < 0.5\, \mu$G for 1-PeV electrons). On the other hand, if the emission is of hadronic origin, a more reasonable spectrum of protons can be obtained. In this case, since the spectrum does not show a clear cutoff feature, V4641 Sgr would be a so-called super-PeVatron, energizing protons to energies of at least ${\sim }10$ PeV.

*GRS 1915+105.* As the first known galactic object ejecting matter with relativistic motion [47], GRS 1915+105 is the famous archetypal microquasar. We discovered an extended source, LHAASO J1915+1053 (${\rm RA}=288.74^\circ \pm 0.05^\circ$, ${\rm DEC}=10.88^\circ \pm 0.05^\circ$), located at $0.1^\circ$ southwest of GRS 1915+105. There are other potential high-energy particle accelerators, such as PSR J1914+1054g and SNR G045.7-00.4, around the LHAASO source, but GRS 1915+105 is most likely the counterpart (see the online supplementary material for detailed discussion). Interestingly, ALMA has discovered two hotspots, both at $0.28^\circ$ (corresponding to about 50 pc) from GRS 1915+105 in opposite directions from the BH [48], which may be interpreted as two lobes driven by jets. Recent observation by MeerKAT supports this picture [49]. Indeed, the angular separation between the two ALMA hotspots is comparable to the extent of the LHAASO source with $r_{39}=0.28^\circ$. This could be due to the large distance of GRS 1915+105 (i.e. 9.4 kpc [33]), such that LHAASO does not resolve their emission. Alternatively, the extended nature of the source and the offset from the BH may be ascribed to the spatial distribution of the surrounding gas, if the emission from GRS 1915+195 is dominated by hadronic processes.

*MAXI J1820+070 and Cygnus X-1*. Two point-like sources, LHAASO J1821+0723 (${\rm RA}=275.22^\circ \pm 0.05^\circ$, ${\rm DEC}=7.39^\circ \pm 0.06^\circ$) and LHAASO J1957+3517 (${\rm RA}=299.47^\circ \pm 0.13^\circ$, ${\rm DEC}=35.37^\circ \pm 0.06^\circ$), were discovered in spatial association to MAXI J1820+070 and Cygnus X-1, respectively. MAXI J1820+070 was discovered in 2018 during its X-ray outburst [50]. A pair of bipolar relativistic radio ejecta was launched from the central black hole during the outburst [51]. Although the LHAASO source is detected at $0.27^\circ$ northeast of the BH (corresponding to a $3.5\sigma$ offset), it is in alignment with the propagation direction of the receding ejecta. There is also a moderate offset of $0.19^\circ$ (corresponding to $1.6\sigma$) between Cygnus X-1 and the associated LHAASO source. The offset follows the same direction as the radio-emitting bow-like structure (shown as the cyan arc in Fig. 2d) that appears to be inflated by the jet launched from the central BH [30]. Extensions of these two LHAASO sources are not evident, and in turn we obtain upper limits of $0.28^\circ$ and $0.22^\circ$ for the sizes of J1821+0723 and J1957+3517 respectively. While we do not expect variability of emission from extended sources, the flux of a point-like source may in principle vary with time. We therefore check their temporal behaviors, but find no evidence of variability. Future continuous observation of LHAASO is crucial for further clarification of the morphological, spectral and temporal properties of these two sources.

## DISCUSSIONS AND CONCLUSIONS

Among the five microquasars with teraelectronvolt–petaelectronvolt emission, SS 433 and Cygnus X-1 exhibit persistent BH activity, whereas V4641 Sgr and GRS 1915+105 have shown frequent radio or X-ray outbursts in recent years [52,53]. On the other hand, most of the seven microquasars without significant LHAASO detection have presented only one historical outburst leading to their discoveries (with the exception of V404 Cyg, which underwent outbursts in 2015 [51]). While the timescales for any of the relevant radiation mechanisms are too long to directly link the activity in the last few decades to the UHE emission, the observed pattern implies that the UHE emission serves as a proxy for the long-term activity status of the BH binaries. In that case, the subset of undetected microquasars with frequent flaring activity would be the most likely candidate for further UHE discoveries. Conversely, if the apparent connection is coincidental, we would expect UHE emission to be associated with many currently quiescent BH binaries that remain unidentified. Future joint observations of LHAASO and X-ray/radio instruments may elucidate this.

Detection of UHE gamma-ray emission from multiple microquasars establishes that BH-jet systems are potent particle accelerators. The energy-dependent morphology of SS 433 and the spatial coincidence between its UHE emission and a nearby atomic cloud suggests a scenario of coexisting leptonic and hadronic emission components, supporting its capacity to accelerate protons to the ‘knee’ of the CR spectrum. Meanwhile, the spectrum of V4641 Sgr has been measured up to 800 TeV, further positioning microquasars as potential super-PeVatrons.

A crucial follow-up question is whether these galactic BH-jet systems are responsible for the origin of CRs above petaelectronvolt energies. Extrapolating from SS 433’s petaelectronvolt proton luminosity inferred from our model ($L_{p, \rm PeV}\lesssim 10^{38}\, \rm erg\, s^{-1}$), we estimate that the Milky Way’s microquasar population could collectively inject petaelectronvolt protons at $f_{\mu \rm Q}L_{p,\rm PeV}=10^{39}\,\rm erg\, s^{-1}$, where $f_{\mu \rm Q}\sim 10$ accounts for the scaling factor in source number and power of the entire population in the Milky Way. This matches the observed petaelectronvolt proton flux on Earth within the leaky-box model framework (see the online supplementary material for further details). The acceleration mechanisms and acceleration sites in these BH-jet systems, on the other hand, have not been clearly identified based on our current observations. In principle, particle acceleration may be processed at different scales and environments, ranging from the vicinity of BHs to the endpoints of jets (such as re-collimation shocks and termination shocks [54,55]). Future observations with higher statistics and dedicated analyses of each microquasar, along with multiwavelength observations, can enable more detailed spectral, morphological and temporal measurements. This will facilitate our understanding of physical processes in these galactic stellar-mass BH-jet systems, and provide insights into UHECR acceleration in their more powerful siblings in distant universe harboring supermassive BHs, such as radio galaxies and blazars.

## LHAASO DATA

In this work, we utilize the data acquired by the LHAASO-KM2A 1/2 array from 26 December 2019 to 30 November 2020 (with a live time of 290 days), by the 3/4 array from 1 December 2020 to 19 July 2021 (with a live time of 216 days) and by the full array from 20 July 2021 to 31 December 2024 (with a live time of 1228 days). The performance of LHAASO-KM2A has been studied in detail using Monte Carlo simulations [56] and calibrated with measurements of the Crab Nebula as a standard candle [57] (see also the online supplementary material). The data quality control system and long-term performance monitoring of the LHAASO-KM2A data are described in [58]. The pipeline for the LHAASO-KM2A data analysis presented in [57] is adopted directly in this analysis. Considering the energy resolution and statistics, one decade of energy is divided into five bins with a bin width of $\log _{10}E=0.2$. The sky in celestial coordinates is divided into grids of size $0.1^{\circ } \times 0.1^{\circ }$ and filled with detected events according to their reconstructed arrival directions for each energy bin. The background map is estimated by the direct integration method [59].

The LHAASO-WCDA data adopted in this analysis cover the period from 8 March 2021 to 31 May 2024, with a total live time of 1092 days. Events with a zenith angle larger than 50$^{\circ }$ or an RMDS larger than 20 m are not included to maintain good event quality, where RMDS is the root mean square distance of the top 10 hottest detector units, representing the compactness of lateral distribution of air showers. The number of fired detector units, $N_{\rm hit}$, is selected as the estimator of primary energy. The events are divided into six segments with $N_{\rm hit}$ values of 60–100, 100–200, 200–300, 300–500, 500–800 and 800–2000. We do not use events of larger $N_{\rm hit}$ because, when $N_{\rm hit}$ is larger than 2000, the instrument is close to saturation and the energy resolution is poor. The direct integration method is adopted to estimate the cosmic ray background.

Assuming a simple power-law spectrum model and a Gaussian spatial model for the LHAASO sources, an iterative process based on multi-dimensional maximum likelihood is performed to derive the spectrum, position and extension. The iteration process adds one source at a time to the fit, as long as the test statistics of $N+1$ sources exceeds that of *N* sources by more than 25. The dust column density measured by PLANCK is introduced as the spatial template of diffuse galactic gamma-ray emission. For further details, see the online supplementary material.

## Supplementary Material

nwaf496_Supplemental_File
