# Supplementary material for: Ultrahigh-energy gamma-ray emission associated with black hole–jet systems
Source: Natl Sci Rev. 2025 Nov 16;12(12):nwaf496. doi: 10.1093/nsr/nwaf496 (PMC12746838; doi:10.1093/nsr/nwaf496)
Supplement: nwaf496_Supplemental_File [file nwaf496_supplemental_file.pdf]

Supplementary Materials

1 Analysis details

1.1 The LHAASO-KM2A data

As one of the three sub-arrays of the Large High Altitude Air Shower Observatory (LHAASO), KM2A is mainly designed for the study of cosmic rays (CRs) and gamma-rays at energies above 10 TeV[1]. The KM2A is comprised of 5,216 electromagnetic particle detectors (EDs) and 1,188 muon detectors (MDs). The EDs were used to record the sequence of arrival times and deposited energy of the secondary particles to reconstruct the direction and energy of a primary Cosmic Ray (CR) or gamma-ray event. MDs were used to record the numbers of muons to distinguish gamma-ray-induced showers from CR-induced ones.

To achieve better performance of the array, several event selection conditions were applied: (1) the zenith angle of the reconstructed directions is less than 50°. (2) The number of triggered EDs ($NtrigE$) and the number of deposited particles used in the shower reconstruction are both larger than ten. (3) The number of particles recorded by EDs within 100 m from the shower core ($NpE1$) is twice larger than that recorded within 40-100 m ($NpE2$). (4) The distance from the shower core to the edge of the array (dr) is greater than 0 m for the 1/2 KM2A array, or greater than 20 m for the 3/4 KM2A and full arrays. (5) The shower age (age) is within 0.6-2.4, where the age of 0 indicates the first interaction time point, 1 represents the peak of the shower development.

The performance of KM2A has been carefully studied with the simulation data. The energy range of the simulation data is from 1 TeV to 10 PeV. In this work, the air showers produced by primary particles were simulated with the $CORSIKA$ code (version 7.6400) [2]. A specific software, G4KM2A [3, 4], was developed in the framework of the Geant4 package[5] to accurately simulate the KM2A detector response. The validity of the simulation has been checked using the observation on the standard candle Crab Nebula [6, 7, 8]. In the normal data analysis pipeline of KM2A, only events with zenith angle less 50° are used. To extend the field of view of KM2A, events with zenith angle ranging from 50° to 60° are also used in this work for the observation of V4641 Sgr.

The effective area of KM2A for detecting gamma-ray showers is energy and zenith angle dependent. Supplementary Figure 1 shows the effective area of KM2A with full configuration at five zenith angles $\theta = 30°$, 50°, 55°, 60° and 65°. The effective area increases with energy and gradually reaches a constant value at energies above 10 TeV for zenith angles less than 30°. However, the energy at which the effective area reaches the constant value increases quickly as the zenith angle increases. The effective area reaches a constant value at energies of $\sim$ 100 TeV, 300 TeV and 1000 TeV for zenith at 50°, 55° and 60°, respectively.

For each event recorded by KM2A, the direction of the air shower are derived from the recorded hit time and charge. The detailed method can be found in the previous studies[6]. The angular resolution of the KM2A for detecting gamma-ray showers is also energy and zenith angle dependent. Supplementary fFigure 1 also shows the angular resolution of KM2A for gamma-rays at five zenith angle bins, i.e., $0° - 30°$, $30° - 50°$, $50° - 55°$, $55° - 60°$, and $60° - 65°$. For showers with a zenith angle less than 30°, the angular resolution ranges from 0.5° at 20 TeV, 0.2° at 100 TeV to 0.1° at 1000 TeV. For showers with a zenith angle ranging from 55° to 60°, the angular resolution ranges from 0.7° at 100 TeV to 0.2° at 1000 TeV.

The energy reconstruction method of the KM2A events has been introduced in detail in the previous ref.[6] using the particle density at 50 m from the shower core and taking into account the zenith angle of the events. Supplementary Figure 1 shows the energy resolution of KM2A for gamma-rays at same five zenith angle bins used in presenting the angular resolutions. For gamma-rays with energy above 100 TeV, the energy resolution ranges from 32% to 7% at zenith angle less than 50° (for more details see ref.[6]), and the resolution will be around 65% to 30% at zenith angle ranging from 50° to 60°.

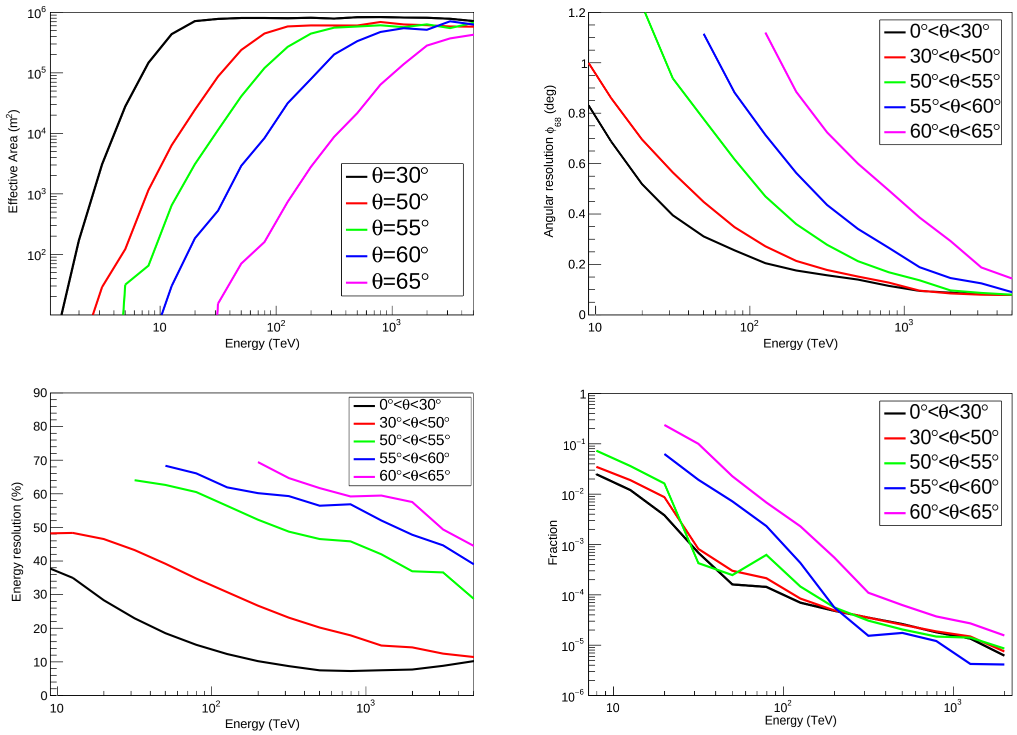

Supplementary Figure 1: The performances of KM2A with full configuration. Top left: The effective area of the KM2A for gamma-ray showers as function of energy at five zenith angles. Top right: The angular resolution of KM2A for gamma-rays as function of energy at five zenith angle ranges. Bottom left: The energy resolution of KM2A for gamma-rays as function of energy at five zenith angle ranges. Bottom right: The residual fraction of cosmic ray background after using the gamma-ray/cosmic ray discrimination cut.

Most of the events recorded by KM2A are cosmic-ray induced showers, which constitute the dominating background for gamma-ray observations. Considering that gamma-ray induced showers are muon-poor and cosmic ray induced showers are muon-rich, the muon content of each shower is used. More details about this particle identification processes can be found in the previous ref.[6]. The rejection power depends on the cutting parameter. A stricter cut will yield a stronger rejection power for cosmic ray background, however, the survival fraction for gamma-ray will also be reduced. The cut parameter for events with zenith angle less than 50° is same as that presented in ref.[6]. The cut parameter for events above 50° is optimized to balance the survival fraction between gamma-ray signals and the CR background. The survival fraction for CRs at different energies and zenith angle ranges are shown in Supplementary Figure 1. The rejection power of CR-induced showers is better than 4000 at energies above 100 TeV with zenith angle less than 50°. For events of zenith angle ranging from 50° to 60°, the rejection power is lower than that at $\theta \leq 50°$ by a factor of $10 - 100$ around 100 TeV. With increasing energy, the difference between the rejection power for large-zenith-angle events and small-zenith-angle events become less (see Supplementary Figure 1).

The high-energy gamma-ray data above 10 TeV used in this work was collected by the KM2A 1/2 array from December 27, 2019, to November 30, 2020 (with a live time of 289.6 days), by the KM2A 3/4 array from December 1, 2020, to July 19, 2021 (with a live time of 215.9 days), by the KM2A full array from July 20, 2021, to December 31, 2024 (with a live time of 1227.8 days). The long-term stability of the data performance has been thoroughly checked and monitored [9].

1.2 The LHAASO-WCDA data

Data used in this analysis were collected by full array of LHAASO-WCDA from March 5, 2021 to May 31, 2024, with a live time of 1092 days. The event selection conditions include:

- The number of triggered detector units $N_{\rm hit}$, which is selected as a shower energy estimator [10], is divided into six segments: $60 <= N_{\rm hit} < 100, 100 <= N_{\rm hit} < 200, 200 <= N_{\rm hit} < 300, 300 <= N_{\rm hit} < 500, 500 <= N_{\rm hit} < 800$ and $800 <= N_{\rm hit} <= 2000$;

- The reconstructed zenith angle is less than 50°;

- The parameter $\mathcal{P}$, in which both $< \zeta_{\rm i} >$ and $\sigma_{\zeta_{\rm i}}$ are extracted from a sample set of gamma-like events, is applied to exclude hadronic showers [11]. $\mathcal{P}$ cut of six $N_{\rm hit}$ segments are 1.02, 0.90, 0.88, 0.88, 0.84 and 0.84.

- RMDS is the shower core location error based on top 10 of hottest detectors. RMDS is required to be smaller than 20.

Events with $N_{\rm hit}$ larger than 2000 are not included because WCDA is close to saturation and the energy resolution becomes quite poor. The total number of gamma-like event after selection is 1.02×10^{10}. Detector simulation based on Geant4 software package is well described elsewhere [10]. The simulation data used in this analysis are generated following the trajectory of Crab nebula, so there is no events with zenith angle less than 7.3°. The primary energy ranges from 1 GeV to 1 PeV and follows a power-law spectrum with index of -2.6.

1.3 Analysis process

In this study, KM2A data sets are divided into five energy bins per decade according to the reconstructed energy with a bin width of $\Delta \log_{10} E = 0.2$. The sky map in equatorial coordinates (right ascension and declination) is divided into spatial grids of 0.1°× 0.1°, and each of such a grid is called a pixel. The "direct integral method" [12] is adopted to estimate the number of CR background events in each pixel. In this work, the integrated time is 10 hr and the events within the regions of the Galactic plane ($|b| < 10°$) and VHE gamma-ray sources (with angular distance less than 5°) are excluded to estimate the background.

A 3D Likelihood method is used in this study. To apply the method to LHAASO data, the first step is to build a source model characterized by the position and spectrum of the source. The source spectrum in the present analysis is assumed to follow a certain function, e.g. a single power-law, a power-law with

exponential cutoff, a log-parabola and et al.. Taking the single power-law as an example:

$$\frac{dN}{dE} = N_0 \times (\frac{E}{E_0})^{-\alpha} \tag{1}$$

where N_0 is the differential flux normalisation, E_0 is the reference energy. The parameters are evaluated using the forward-folding method. The source model is convolved with the detector response, which is established through Monte Carlo simulations mentioned above, to derive the expected gamma-ray signal in each spatial pixel of each energy bin. The diffuse Galactic gamma-ray emission (GDE) resulting from the interaction of CRs with the ISM and background photons is an essential component of the gamma-ray sky, which has already been measured by LHAASO[13, 14]. The GDE is also taken into account in this work. Following the treatment in ref.[15], we used the distribution of the dust optical depth in the sky measured by Planck [16], which can be translated to the spatial distribution of the column density of gas, as the spatial template of GDE in the region of interest (ROI). We assume the spectrum of GDE to be a power-law function in the energy range of KM2A, treating the normalisation factor and spectral index as free parameters (the same for WCDA analysis, but with independent spectral parameters from KM2A), which will be obtained together with those of sources in the fitting. The maximum likelihood fit is performed by comparing the observed signal to the expected signal. Taken into account both the contribution from sources and background, the expected event counts can be expressed as

$$\lambda_{ijn_s} = b_{ij} + \sum_{1}^{k} n_{s,ijk}, \tag{2}$$

where b_{ij} is the number of background events in the jth pixel of ith energy bin, and $n_{s,ijk}$ the expected number of gamma ray counts from the kth source in the jth pixel of ith energy bin. The observed events count in each pixel is distributed according to a Poisson distribution, the probability of observing N number of events given an expected counts λ from the source model is

$$P(\lambda \mid N) = \frac{\lambda^N e^{-\lambda}}{N!} \tag{3}$$

The logarithm of the likelihood value is defined as :

$$\ln \mathcal{L}(\vec{\theta} \mid \vec{n}) = \sum_{i}^{E_{\rm bins}} \sum_{j}^{\rm ROI} (N_{ij} \ln \lambda_{ij} - \lambda_{ij}) \tag{4}$$

The log-likelihood is maximized with respect to the parameter set $\vec{\theta}$ in the source model using the ROOT TMINUIT package.

A likelihood ratio test is performed to decide how many sources are needed to properly model in the ROI. The log-likelihood of the background-only model $ln\mathcal{L}_0$ and the log-likelihood $ln\mathcal{L}_1$ of the one-source model is computed, respectively. The test statistic (TS) defined as

$$TS = -2(\ln \mathcal{L}_0 - \ln \mathcal{L}_1) \tag{5}$$

is used to compare the goodness of one model over the other. The TS-value is converted to a p-value, which is the probability of the data being consistent with the background-only hypothesis. We add the source to the model when TS is larger than 25. The procedure is iterated to determine the number of sources in the ROI. To obtain the flux point of a source in each energy bin, we fix the spectral index in each energy bin to the best-fit index obtained for the entire energy range, while the normalisation factor of the spectrum in each energy bin are still set as a free parameter. The flux point in each energy bin is calculated based on the spectral index and the normalisation factor. If TS value of an energy bin is smaller than 4, a flux upper limit with 95% confidence level will be given by the likelihood profile method.

In this study, we also considered systematic uncertainties of the results. There are three main possible origins of the systematic errors. The first is the impact of GDE. To estimate its influence, we artificially scaled the normalisation factor of GDE by ±20%, based on the best-fit model, while keeping its best-fit spectral index unchanged, and re-fitted the sources in the ROI. The second is the impact of the extensions of the

neighbouring sources. We fix the positions of neighbouring source to the best-fit values, increase (decrease) their spatial extensions to the 90% confidence level (C.L.) upper (lower) bound with respect to the best-fit values, and refit the target source. During this process, we keep the GDE component unchanged as the best-fit values. Another systematic error comes from the atmospheric model used in the Monte Carlo simulations due to seasonal and daily changes. The total systematic uncertainty for KM2A is estimated to be 7% for the flux and 0.02 for the spectral index [6]. For WCDA, the systematic uncertainty is estimated to be 8% for the flux [15]. The uncertainties derived from the above three origins were combined as the root sum square to obtain the final systematic uncertainties. In the following part of the paper, we report only statistical uncertainties of measured quantities unless stated otherwise. Only SS 433 is detected by WCDA, so below we do not report WCDA results on other sources as the focus of this work is about the UHE emission.

2 Results

2.1 SS 433

2.1.1 The LHAASO observation of SS 433

SS 433 (RA=287.96°, DEC=4.98°, or $l = 39.69°$, $b = -2.24°$) hosts a 12.3±3.3$M_\odot$ companion star[17] and a BH with mass >8$M_\odot$[18]. In this study, an ROI with a radius of 6° centered on the right ascension (RA) 287.0° and the declination (DEC) 6.4° is used. The significance map of this ROI is shown in Supplementary Figure 2. Through iterative searches, we identified 15 sources in this region using KM2A data, and the best-fit results are shown in Supplementary Table 1 and Supplementary Table 2. "ps" in the tables means point-like source. Among these sources, LHAASO J1908+0614 (which is likely associated with MGRO J1908+06) and LHAASO J1909+0542 (which is newly identified without apparent counterpart, but could still be related to MGRO 1908+06) are close to SS 433 and their influence on our results will be further studied below. Other 11 sources are at least 2° away from SS 433 and not very bright, so their influence on the results is limited. Supplementary Figure 4 shows the residual significance map of the ROI and Supplementary Figure 5 shows the distribution of residual significance at different energy ranges, which are in general in consistency with the normal distributions[1].

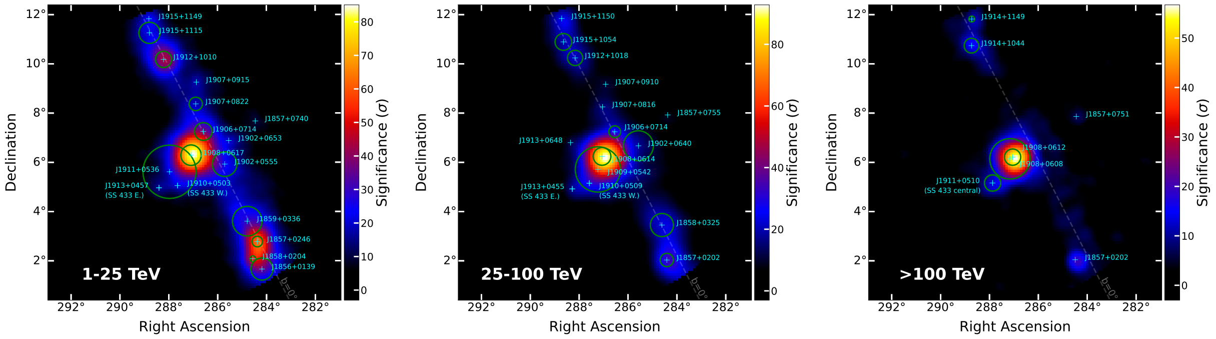

Supplementary Figure 2: The significance map of MGRO J1908+06 region at 1-25 TeV (left)., 25-100 TeV (middle) and >100 TeV (right). The position and 39% containment radius of resolved LHAASO sources are marked by cyan cross and green circle, respectively. The name of LHAASO sources are indicated by cyan text.

[1]In the right panel of Supplementary Figure 5 (for $E > 100$ TeV), there is a slight excess around -2.5σ. This is due to the number of background events (i.e. N_b) is extremely low (may be less than 1), and the minimum number of events in the source region (i.e., N_on) is zero. This leads to a reduction of the minimum negative values, and the distribution accumulates around -2.5σ. Such a distribution presented in the panel is not abnormal and does not affect the accurate estimation of gamma-ray signals.

Supplementary Table 1: Best-fit model parameters of SS 433 region at 25 − 100 TeV.

Name LHAASO	RA (∘)	DEC (∘)	σ_{ext} (∘)	significance (σ)	Possible Association
J1908+0614	287.06±0.01	6.24±0.01	0.36±0.01	54.6	1LHAASO J1908+0615u<br>MGRO J1908+06
J1909+0542	287.23±0.14	5.71±0.14	0.92±0.10	13.0	new source
J1902+0640	285.57±0.20	6.67±0.18	0.61±0.16	5.4	new source
J1906+0714	286.54±0.12	7.24±0.12	0.24±0.09	8.0	new source
J1913+0648	288.35±0.05	6.80±0.08	ps	10.9	new source
J1913+0455	288.28±0.04	4.92±0.04	ps	9.9	1LHAASO J1913+0501<br>SS 433 E.
J1910+0509	287.58±0.06	5.15±0.05	ps	6.3	1LHAASO J1910+0516<br>SS 433 W.
J1857+0202	284.41±0.04	2.03±0.03	0.27±0.02	16.4	1LHAASO J1857+0203u<br>HESS J1858+020
J1858+0325	284.61±0.03	3.45±0.04	0.47±0.03	26.3	1LHAASO J1858+0330
J1912+1018	288.16±0.07	10.24±0.07	0.31±0.05	11.6	1LHAASO J1912+1014u<br>HESS J1912+101
J1915+1054	288.64±0.06	10.89±0.04	0.34±0.04	8.1	GRS 1915+101
J1915+1150	288.71±0.04	11.84±0.04	ps	10.7	1LHAASO J1914+1150u<br>2HWC J1914+117
J1907+0816	287.04±0.04	8.25±0.03	ps	8.2	1LHAASO J1907+0826<br>2HWC J1907+084
J1857+0755	284.37±0.06	7.92±0.06	ps	7.8	new source
J1907+0910	286.91±0.02	9.17±0.02	ps	4.1	new source

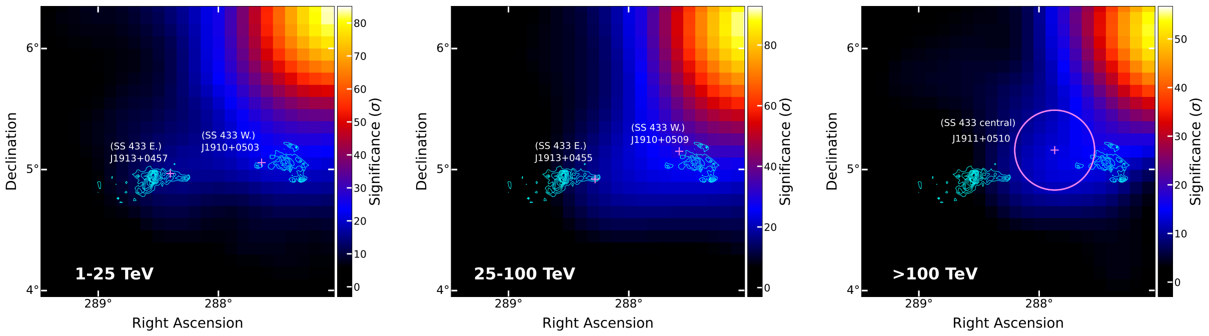

Supplementary Figure 3: Zoom-in version of significance maps of MGRO J1908+06 region at 1-25 TeV (left)., 25-100 TeV (middle) and >100 TeV (right). Cyan contours show the X-ray emission.

Supplementary Table 2: Best-fit model parameters of SS 433 region above 100 TeV.

Name LHAASO	RA (∘)	DEC (∘)	σ_{ext} (∘)	significance (σ)	Possible Association
J1908+0612	287.05±0.02	6.21±0.03	0.33±0.03	31.5	1LHAASO J1908+0615u MGRO J1908+06
J1908+0608	287.16±0.09	6.14±0.09	0.82±0.07	9.6	new source
J1857+0202	284.49±0.03	2.04±0.03	ps	14.1	1LHAASO J1857+0203u HESS J1858+020
J1914+1044	288.73±0.09	10.74±0.07	0.30±0.07	9.6	GRS 1915+101/HESS J1912+101
J1914+1149	288.72±0.07	11.82±0.08	0.12±0.06	6.3	1LHAASO J1914+1150u 2HWC J1914+117
J1913+0751	284.44±0.03	7.86±0.03	ps	4.3	new source
J1911+0510	287.87±0.12	5.16±0.12	0.32±0.04	8.0	SS 433 central

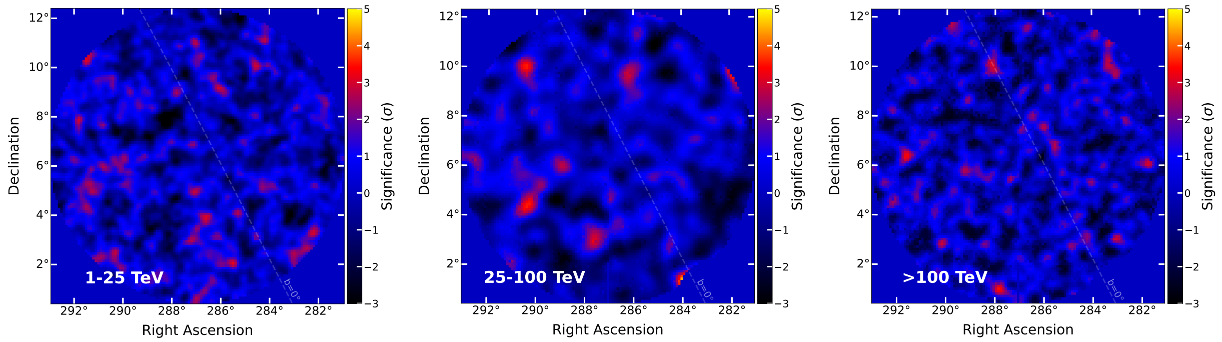

Supplementary Figure 4: The residual significance map of the ROI used in this work near MGRO J1908+06 region at 1-25 TeV (left), 25-100 TeV (middle) and >100 TeV (right).

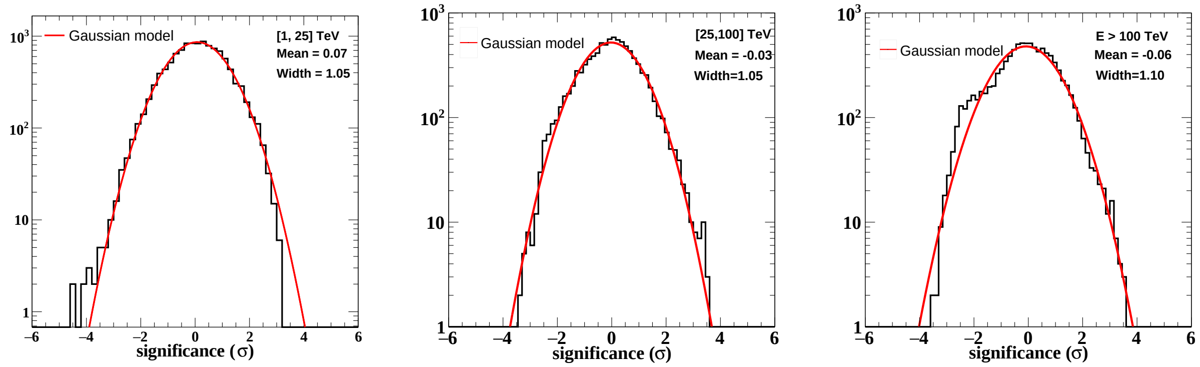

Supplementary Figure 5: The distribution of residual significance within the ROI for analysis of SS 433 at $1-25\,\text{TeV}$ (left), $25-100\,\text{TeV}$ (middle), and $>100\,\text{TeV}$ (right). In each panel, the red line is the fitting to the distribution with a Gaussian function. Its mean and width are labeled in the figure.

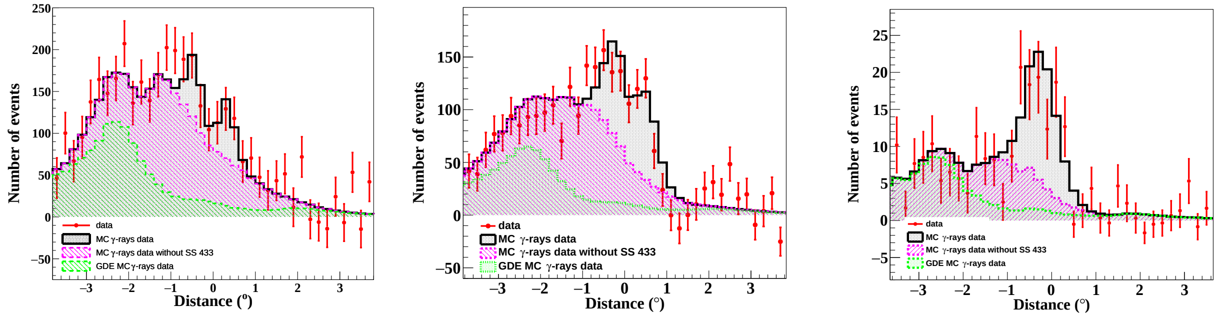

Supplementary Figure 6: 1D profile of event distribution along the jets of SS 433. The x-axis shows the distance to the center of the two jets. The data points represent detected events in spatial bins of 0.2° along the axis joining both jets for energies at $5-25$ TeV (left), $25-100$ TeV (middle) and above 100 TeV (right). The histograms show the expected gamma-ray data from different components, showing the contamination of GDE and other detected sources in the ROI.

The bright extended source MGRO J1908+06 is located less than 2° away from the position of SS 433. In our analysis, the brightest source resolved in this region, LHAASO J1908+0614, is detected at a significance of more than 60σ above 25 TeV. The best-fit position of the source derived from the KM2A data is RA $= 287.06^\circ \pm 0.01^\circ$ and DEC $= 6.23^\circ \pm 0.01^\circ$ with an extension of $0.36^\circ \pm 0.01^\circ$, which is consistent with that in the LHAASO catalog paper[15]. It is likely associated with MGRO J1908+06. In addition, a few other sources are resolved in the surrounding region. They constitute the main background sources for the analyses of SS 433. We evaluate the potential contamination of these sources to SS 433 as well as of GDE as follows. Supplementary Figure 6 shows the distribution of the events as the profiles along the SS 433 jets which shows the contamination of GDE, MGRO J1908+06 and other detected sources in the ROI. To quantify the impact of the MGRO J1908+06 on the SED of SS 433, we first fixed the extension of MGRO J1908+06 to the 90% upper and lower limits, and then re-calculated the energy spectrum of SS 433. The results indicate that the effect of extension of MGRO J1908+06 on the SED of SS 433 ranges from approximately $\pm5\%$ to $\pm10\%$. The Galactic Longitude of SS 433 is 40°, where the gas column density in the line of sight is relatively high, and the contamination introduced by GDE may also have impact on our final results. By re-scaling the GDE flux by $\pm20\%$ with respect to the best-fit value, we found that it would cause an change of approximately $\pm10\%$ to $\pm20\%$ on the SS 433 flux. We attribute all these uncertainties to the systematic errors and have taken them into account in the discussions in the main text. At above 100 TeV, we carried out a conservative test. We fixed the extension of MGRO J1908+06 to the 90% upper limit and scaled the GDE flux by $+20\%$ with respect to the best-fit value, and repeated the analysis of SS 433. All results are consistent with that reported in the paper. The conservative estimation of pointing error is 0.04° for WCDA[15] and 0.008° for KM2A in 25-100 TeV, 0.023° above 100 TeV[9]. Their impact to data analysis are minimum comparing to GDE.

The data analysis procedure for WCDA data is basically the same as that for KM2A data. The power-law function and the 2D Gaussian function are taken as the spectrum model and spatial template of sources. Based on the above mentioned iteration methods, 17 sources are resolved in the ROI using 1092 days of WCDA data. The best-fit results are listed in Supplementary Table 3. The significance map of the ROI before and after subtracting all the 17 sources and GDE are shown in Supplementary Figure 2 and a zoom-in version are shown in Supplementary Figure 3. The residual significance map shown in Supplementary Figure 5 well follows a standard normal distribution which means sum of the 17 sources and GDE can fit the data appropriately. It should be noted that although the significance of J1857+0740 in the Supplementary Table 3 is less than 5σ, it is still included in the WCDA analysis because the significance of the source in KM2A (J1857+0755 in Supplementary Table 1) is high enough. LHAASO J1913+0457 and LHAASO J1910+0503 are associated with SS 433. LHAASO J1908+0617 (1LHAASO J1908+0615u) is associated with MGRO J1908+06. The position and extension of this source obtained here are consistent with that in the LHAASO catalog paper[15]. Based on the same method, the resulting systematic effect of extension of LHAASO J1908+0617 on the SED of SS 433 ranges from approximately $\pm5\%$ to $\pm20\%$. The re-scaling of the GDE flux by $\pm20\%$ with respect to the best-fit value would cause an change of approximately $\pm7\%$ to

±15% on the SED of SS 433. Our data analyses found that the spectrum of the west point-like source (or

Supplementary Table 3: Best-fit parameters of SS 433 region at $1-25\,\text{TeV}$.

Name LHAASO	RA (∘)	DEC (∘)	σ_{ext} (∘)	significance (σ)	Possible Association
J1856+0139	284.19 ± 0.06	1.66 ± 0.08	0.45 ± 0.05	16.5	new source
J1858+0204	284.56 ± 0.03	2.08 ± 0.03	0.12 ± 0.04	15.8	1LHAASO J1857+0203u<br>HESS J1858+020
J1857+0246	284.38 ± 0.01	2.78 ± 0.02	0.21 ± 0.02	34.7	1LHAASO J1857+0245<br>HESS J1857+026
J1859+0336	284.79 ± 0.06	3.61 ± 0.07	0.60 ± 0.05	16.9	1LHAASO J1858+0330
J1913+0457	288.40 ± 0.05	4.97 ± 0.04	ps	5.4	1LHAASO J1913+0501<br>SS 433 E.
J1910+0503	287.64 ± 0.05	5.06 ± 0.03	ps	5.7	1LHAASO J1910+0516<br>SS 433 W.
J1911+0536	287.97 ± 0.20	5.62 ± 0.10	1.08 ± 0.08	13.9	new source
J1902+0555	285.71 ± 0.14	5.93 ± 0.11	0.50 ± 0.12	9.3	new source
J1908+0617	287.09 ± 0.02	6.29 ± 0.02	0.42 ± 0.01	69.1	1LHAASO J1908+0615u<br>MGRO J1908+06
J1902+0653	285.54 ± 0.05	6.88 ± 0.04	ps	5.2	1LHAASO J1902+0648
J1906+0714	286.58 ± 0.06	7.25 ± 0.09	0.36 ± 0.05	14.0	1LHAASO J1906+0712
J1857+0740	284.46 ± 0.05	7.68 ± 0.08	ps	3.9	new source
J1907+0822	286.89 ± 0.08	8.37 ± 0.12	0.27 ± 0.07	5.7	1LHAASO J1907+0826<br>2HWC J1907+084
J1907+0915	286.87 ± 0.03	9.26 ± 0.05	ps	8.0	new source
J1912+1010	288.21 ± 0.02	10.18 ± 0.02	0.33 ± 0.02	32.2	1LHAASO J1912+1014u<br>HESS J1912+101
J1915+1115	288.79 ± 0.07	11.26 ± 0.16	0.43 ± 0.06	7.7	SNR G045.7-00.4
J1915+1149	288.81 ± 0.02	11.83 ± 0.02	ps	11.4	1LHAASO J1914+1150u<br>2HWC J1914+117

LHAASO J1910+0509) can be described by

$$\frac{dN}{dE} = \begin{cases} (3.77 \pm 0.74) \times 10^{-15} \left(\frac{E}{7\,\text{TeV}}\right)^{-2.54\pm0.19} \ \text{TeV}^{-1}\text{cm}^{-2}\text{s}^{-1}, & 1\,\text{TeV} < E \leq 25\,\text{TeV}, \\ (3.58 \pm 0.70) \times 10^{-17} \left(\frac{E}{50\,\text{TeV}}\right)^{-2.94\pm0.38} \ \text{TeV}^{-1}\text{cm}^{-2}\text{s}^{-1}, & 25\,\text{TeV} < E \leq 100\,\text{TeV}. \end{cases} \tag{6}$$

and the spectrum of the east point-like source (or LHAASO J1913+0455) can be described by

$$\frac{dN}{dE} = \begin{cases} (2.91 \times 0.77) \times 10^{-15} \left(\frac{E}{7\,\text{TeV}}\right)^{-2.13\pm0.22} \ \text{TeV}^{-1}\text{cm}^{-2}\text{s}^{-1}, & 1\,\text{TeV} < E \leq 25\,\text{TeV}, \\ (3.96 \pm 0.53) \times 10^{-17} \left(\frac{E}{50\,\text{TeV}}\right)^{-2.82\pm0.16} \ \text{TeV}^{-1}\text{cm}^{-2}\text{s}^{-1}, & 25\,\text{TeV} < E \leq 100\,\text{TeV}. \end{cases} \tag{7}$$

The spectrum of the two point-like sources based on the joint analysis in $1-100\,\text{TeV}$ are

$$\frac{dN}{dE} = \begin{cases} (2.89 \times 0.39) \times 10^{-16} \left(\frac{E}{20\,\text{TeV}}\right)^{-2.36\pm0.11} \ \text{TeV}^{-1}\text{cm}^{-2}\text{s}^{-1}, & \text{west source}, \\ (2.76 \pm 0.29) \times 10^{-16} \left(\frac{E}{20\,\text{TeV}}\right)^{-2.24\pm0.09} \ \text{TeV}^{-1}\text{cm}^{-2}\text{s}^{-1}, & \text{east source}. \end{cases} \tag{8}$$

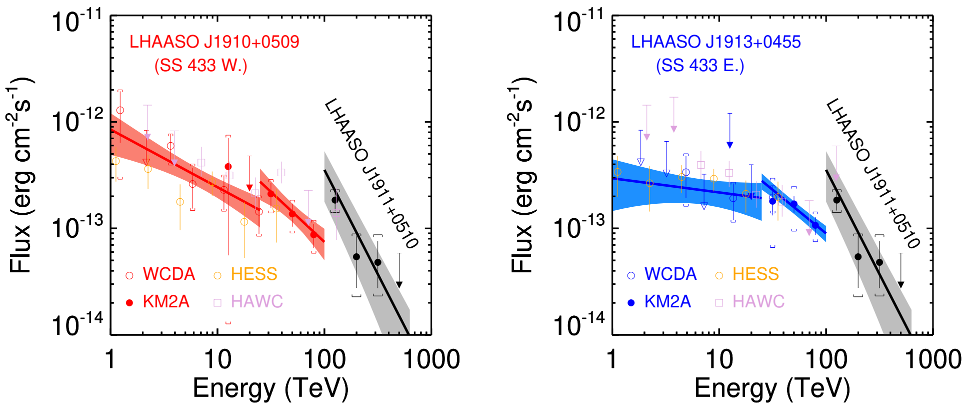

Supplementary Figure 7: Spectra of sources related to SS 433. The left panel shows the spectra of LHAASO J1910+0509 which is associated to the west lobe, while the right panel shows the spectra of LHAASO J1913+0455 which is associated with the east lobe. The spectrum of LHAASO J1911+0510 (the extended source above 100 TeV) is also shown with black points for reference. The red and blue open circles show the data from WCDA while red and blue filled circles show the data from KM2A. Lines represent the best-fit spectrum of the LHAASO data while shaded region shows 1σ uncertainties. Spectra measured by H.E.S.S. and HAWC are shown with orange circles and plum squares.

The spectrum of the the extended source above 100 TeV is

$$\frac{dN}{dE} = (3.45 \pm 1.61) \times 10^{-16} \left(\frac{E}{50\,\mathrm{TeV}}\right)^{-3.96\pm0.25} \mathrm{TeV}^{-1}\mathrm{cm}^{-2}\mathrm{s}^{-1}, \quad E > 100\,\mathrm{TeV} \tag{9}$$

In Supplementary Figure 7 we show the spectra of the two point-like sources associated with SS 433, measured by WCDA and KM2A. We see that in the overlapping energy range of the two instruments, $\sim 10-25$ TeV, the fluxes measured by the two detectors are consistent with each other. The overall spectrum of the GDE may be described by a smoothly broken power-law function, $dN/dE = N_0(E/10\ \mathrm{TeV})^{-\alpha}[1 + (E/E_{\mathrm{br}})^s]^{(\alpha-\beta)/s}$, with the best-fit parameters $N_0 = 3.58 \pm 0.20 \times 10^{-12}\ \mathrm{TeV}^{-1}\mathrm{cm}^{-2}\mathrm{s}^{-1}\mathrm{sr}^{-1}$, $\alpha = 2.62 \pm 0.04$, $\beta = 3.07 \pm 0.04$ and $E_{\mathrm{br}} = 12.28 \pm 3.08$ TeV. Parameter s characterizes the smoothness of the break which is fixed to be 5. This function is derived from the paper on the GDE measurement[14].

2.1.2 Energy dependent morphology analysis of SS 433

The gamma-ray emission of SS 433 region at $1-25$ TeV and $25-100$ TeV could be significantly resolved into two point sources. However, above 100 TeV two-point source model is not significantly preferred over one 2D Gaussian model. The Akaike Information Criterion (AIC) was adopted to compare the fitting of above two models. At above 100 TeV, the two-point source model yields a higher TS value than the 2D Gaussian model with ΔTS = 10. Taking into account the additional degrees of freedom added, the ΔAIC is -4 and the two-point source model is not significantly preferred. However, it suggests that the jet lobes of SS 433 extend their gamma-ray contribution beyond 100 TeV. The fitted 2D Gaussian model above 100 TeV is spatially consistent with a H I atomic clouds, which is at a distance consistent with SS 433 (Supplementary Figure 9), promoting a hadronic origin. Thus, both the hadronic and leptonic components may contribute to the emission above 100 TeV. Considering the limited statistics and particle acceleration sites located by H.E.S.S.[19], we adopted a two-point source model with positions fixed at H.E.S.S. emission above 10 TeV to model the leptonic emission (Supplementary Table 4). The residual gamma-ray emission is shown in Supplementary Figure 8 and it traces the H I cloud distribution (also see green contours in Supplementary Figure 9, panel b). If the gamma-ray emission is potential hadronic, such a spatial correlation would be expected. To test this hypothesis, we use the H I cloud column density distribution as an input spatial template. We produced the H I cloud template using integrated intensity map in the interval of 64-120 km

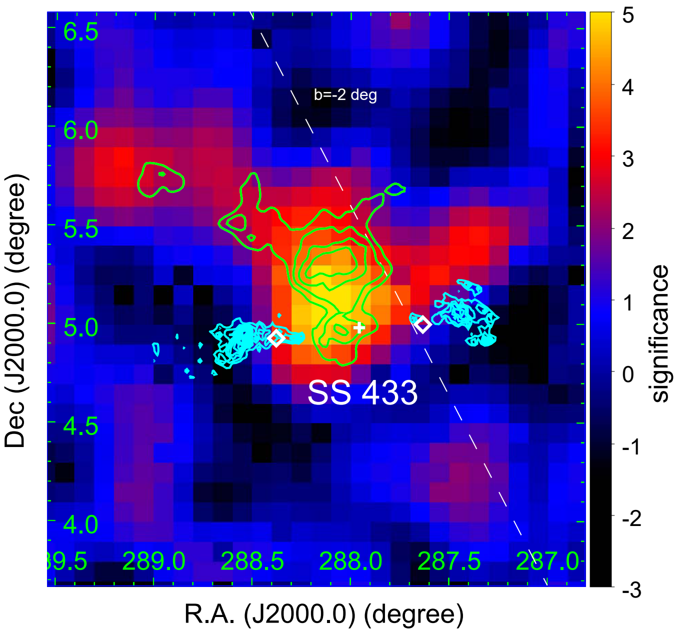

Supplementary Figure 8: Residual significance map of SS 433 region after modeling two-point sources at H.E.S.S. emission above 10 TeV (white diamonds). The green contour shows the distribution of H I gas in Supplementary Figure 9 panel (b), starting from 22 K with a step of 2K. The cyan contour show the X-ray emission of the two lobes. The horizontal and vertical axes are R.A. and decl. in J2000.

s^{-1}. We adopted an arbitrary intensity cut of 350 K km s^{-1} to suppress the noise. Emission region from the Galactic plane was also removed. Adding the H I cloud template to above two-point source model leads to a significant improvement with a ΔAIC of -14.2, supporting the co-existence of hadronic and leptonic components above 100 TeV. Additionally, above two-point source model plus H I cloud template yields a better fit than a 2D Gaussian model with ΔTS = 10.1 and ΔAIC = -8.1. However, it does not reach the criteria of ΔAIC = -10[20] and we report the results of 2D Gaussian model in the main text. We explored the same hadronic and leptonic scenario in the 25-100 TeV data. Comparing to the two-point source model with positions fixed at H.E.S.S. emission above 10 TeV, adding the H I cloud template yields a ΔTS = 4 and ΔAIC = 0. Thus, in 25-100 TeV, the emission component associated to the cloud is not significantly detected. The residual gamma-ray emission also coincide with the central BH, which could also have contributions. The relativistic outflow from the inner part of accretion disk[21] and particles accelerated at the central BH and escaping via diffusion would light up the atomic cloud and produce the observed gamma-ray emission.

2.1.3 Neutral atomic gas analysis around SS 433

The Galactic ALFA H I (GALFA[22, 23]) survey data from the Arecibo Observatory 305 m-telescope was investigated for the neutral atomic gas. These data were first used in the report by ref[24, 25]. The GALFA H I cube data has a grid spacing of 1' and a velocity channel separation of 0.184 km s^{-1}. Typical noise levels are 0.1 K rms of brightness temperature in an integrated velocity of 1 km s^{-1}. Using the Gaussian position and 68% contamination radius (29.0') of SS 433 observed above 100 TeV (main text figure 1), we extracted the average H I spectrum which is shown in Supplementary Figure 9 panel (a). We find that the atomic gas excess in this region peaks at velocities of $V_{\rm LSR} \sim$ 64–73 km s^{-1}, which corresponds to a near kinematic distance of $\sim 3.9 - 4.6$ kpc. For the distance of SS 433, there are several different estimations in the literature (e.g. 4.6±1.3 kpc[26]; 4.9±0.4 kpc[24]; 4.61±0.35 kpc[27]; 5.5±0.2 kpc[28]; $7.29^{+1.19}_{-0.86}$ kpc[29]). Parallax distance estimations can be affected by binary motions and several of the difference above may be attributed to the kinematics of the different regions of the jets (extended jets and inner jets[30]). Thus, We adopted the 4.9±0.4 kpc[24] in this paper, which is derived from the surrounding molecular gas interacting with SS 433 and is similar to the method with which we derived the distance of the atomic gas excess.

Supplementary Table 4: Different models of SS 433 above 100 TeV. The ΔTS and ΔAIC are calculated regarding the 2D Gaussian model.

Model of SS 433 above 100 TeV	Degree of Freedom	ΔTS	ΔAIC
2D Gaussian	5	0	0
two-point sources at H.E.S.S. emission position above 10 TeV	4	-8.1	6.1
two-point sources at H.E.S.S. emission position above 10 TeV + H I gas template	6	10.1	-8.1

Considering the various distance estimations, the distance of the atomic gas excess is roughly consistent with that of SS 433. Towards the direction, there is no prominent H I absorption feature near the tangent velocity of $V_{\rm LSR} \sim 83\,\rm km\ s^{-1}$, excluding SS 433 is located at the far side of the tangent point [31, 24]. Indeed, the recent study [24] has shown that SS 433 is interacting with the surrounding molecular gas at $V_{\rm LSR} \sim 72$–$83\,\rm km\ s^{-1}$, leading to a near kinematic distance of $4.9 \pm 0.4\,\rm kpc$. Therefore, we adopt a lower distance of 4.5 kpc for the center of the atomic cloud, which is located near the tangent point but in front of it. We integrate the H I emission from the peak of $V_{\rm LSR} \sim 64\,\rm km\ s^{-1}$ to $V_{\rm LSR} \sim 120\,\rm km\ s^{-1}$, to avoid contamination from other unrelated gas components, and show the map in Supplementary Figure 9 panel (b). The atomic gas excess is consistent with the gamma-ray emission above 100 TeV. We calculate the H I intensity by integrating the spectrum above the peak of $64\,\rm km\ s^{-1}$ and multiply it by 2, leading to the mean intensity of ~ 1100 K km $\rm s^{-1}$, indicating a total mass of $\geq 1.0 \times 10^5 \rm M_\odot$ by adopting the distance of $\geq 4.5\,\rm kpc$. The volume-averaged density of the H I gas enhancement is estimated to be roughly $n_{\rm H} \leq 13\,\rm cm^{-3}$, assuming a spherical cloud with radius $R_c \geq 38\,\rm pc$. Additionally, the distribution of atomic gas excess traces the residual gamma-ray emission after fitting the two point sources above 100 TeV, suggesting a strong link. We have carried out a similar H I data analysis with HI4PI[32] data and the results are consistent but with lower spectral and angular resolution. We note that a point-like GeV gamma-ray source with periodicity same with the jet's precession period was reported in Ref.[25]. The emission was suggested to originate from interaction between outflow launched from the accretion disk of SS 433 and an ambient compact cloud. The compact cloud might be part of the giant cloud we analysed here, supporting the existence of cloud in proximity of SS 433.

2.1.4 Phenomenological fitting to the spectrum of SS 433

According to the energy-dependent morphology of the TeV emission associated with SS433, we envisage the following scenario: the emission below a few times 10 TeV is dominated by electrons accelerated at jet terminations, while the emission above a few times 10 TeV mainly arises from hadronic interactions between protons and the atomic cloud in proximity of the microquasar[33, 34]. The predicted fluxes from two components are summed up and compared with the total flux of the emission associated with SS 433, as shown in the main text (Figure 1e). Note that below 100 TeV, the total measured flux is obtained by adding the measured fluxes in each energy bin of the two point-like sources (i.e., LHAASO J1913+0455 and LHAASO J1910+0509) together, and uncertainties of the total flux are calculated as the root sum squares of the uncertainties of the two sources. The flux above 100 TeV is just that of the extended source or LHAASO J1911+0510.

To model the IC radiation of electrons, we consider that electrons are injected with a power-law spectrum

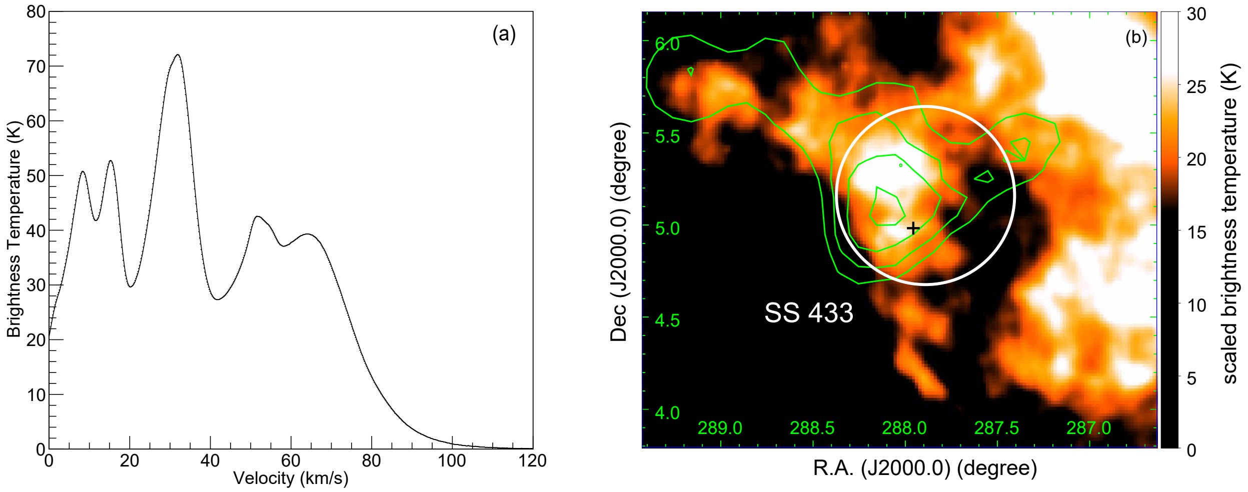

Supplementary Figure 9: Panel (a): H I extracted from the Gaussian position and 68% contamination region of SS 433 above 100 TeV. Panel (b): Map of the H I emission integrated in the interval 64–120 km s^{-1}. The white circle shows the Gaussian profile of SS 433 above 100 TeV with the 68% contamination radius (main text figure 1). The green contours show the residual gamma-ray emission after fitting the two-point sources above 100 TeV, from 2σ with a step of 1σ (Supplementary Figure 8). The image has been scaled by $\sin|b|$ (b is Galactic latitude) to enhance the features far from the Galactic plane[24].

followed by a high-energy cutoff at $E_{\rm e,max}$, i.e.,

$$Q_e(E_{\rm e}) \equiv \frac{dN}{dE_{\rm e}dt} = Q_{0,\rm e}E_{\rm e}^{-s_{\rm e}}\exp(-E_{\rm e}^2/E_{\rm e,max}^2) \tag{10}$$

where $Q_{0,\rm e}$ is the normalisation factor. The E^2 dependence in the cutoff term assumes that the electron acceleration is hindered by radiative energy loss[35]. Measurement of H.E.S.S. suggested $E_{\rm e,max} \geq 200\,$TeV for electrons accelerated in the two lobes. So we test $E_{\rm e,max} = 200\,$TeV and an unrealistically high value of $E_{\rm e,max} = 10\,$PeV. We consider cooling of injected electrons at the magnetic field of the lobes, which we fix at $20\,\mu$G according to previous studies [36, 19], and the interstellar radiation field[37] including the CMB. As a result, the quasi-steady state of the electron spectrum, based on which we calculate the IC radiation, can be given by $N_{\rm e}(E_{\rm e}) \approx Q_{\rm e}(E_{\rm e})\cdot\min[t_{\rm cool}, t_{\rm age}]$. In this equation, $t_{\rm cool}$ is the radiative cooling timescale and $t_{\rm age}$ is the age of the system, which is generally estimated to be between 10 kyr and 100 kyr[38, 39, 40]. In fact, for radiation in the energy range of interest (i.e, above 1 TeV), whether $t_{\rm age} = 10\,$kyr or 100 kyr does not make any difference. We also fit the multiwavelength SED of the west lobe and the east lobe with a simple one-zone leptonic radiation model using the Python package Naima [41]. The electron spectra are modeled following Equation 10. The fitting results and best-fit parameters for the two lobes are presented in Figures 10. The injection spectral index in the west lobe is 2.26 ± 0.13, which requires a magnetic field of $17.6^{+1.8}_{-1.5}\ \mu$G. In the east lobe, the injection spectral index is $2.4^{+0.1}_{-0.2}$, corresponding to a required magnetic field of $22.0^{+2.9}_{-1.9}\ \mu$G. These results are consistent with those reported by H.E.S.S [19]. The cutoff energies are about 9.9 PeV for both regions but neither of them can be well constrained.

To model the hadronic emission, we assume protons are constantly injected at the position of the micro-quasar with a spectrum

$$Q_{\rm p}(E_{\rm p}) = Q_{0,\rm p}E_{\rm p}^{-s_{\rm p}}\exp(-E_{\rm p}/E_{\rm p,max}), \tag{11}$$

where $Q_{0,\rm p}$ is a normalisation factor and its relation to the total proton luminosity can be given by $L_{\rm p} = \int_{1\,\rm GeV} E_{\rm p}Q_{\rm p}(E_{\rm p})dE_{\rm p}$. We then can calculate the spatial distribution of protons assuming they diffuse isotropically in the surrounding medium. The diffusion of protons is dealt with the same method shown in ref.[42]. To model the gamma-ray production, we locate a spherical atomic cloud of $10^5\,M_\odot$ with a radius $R_c = 40\,$pc, centred at a distance of r_c from the microquasar, according to the analysis shown above. For simplicity, we assume a homogeneous gas density within the cloud. Note that the distance r_c can be larger than the projected distance in the sky, so it is considered as a free parameter with a lower limit of 20 pc (roughly

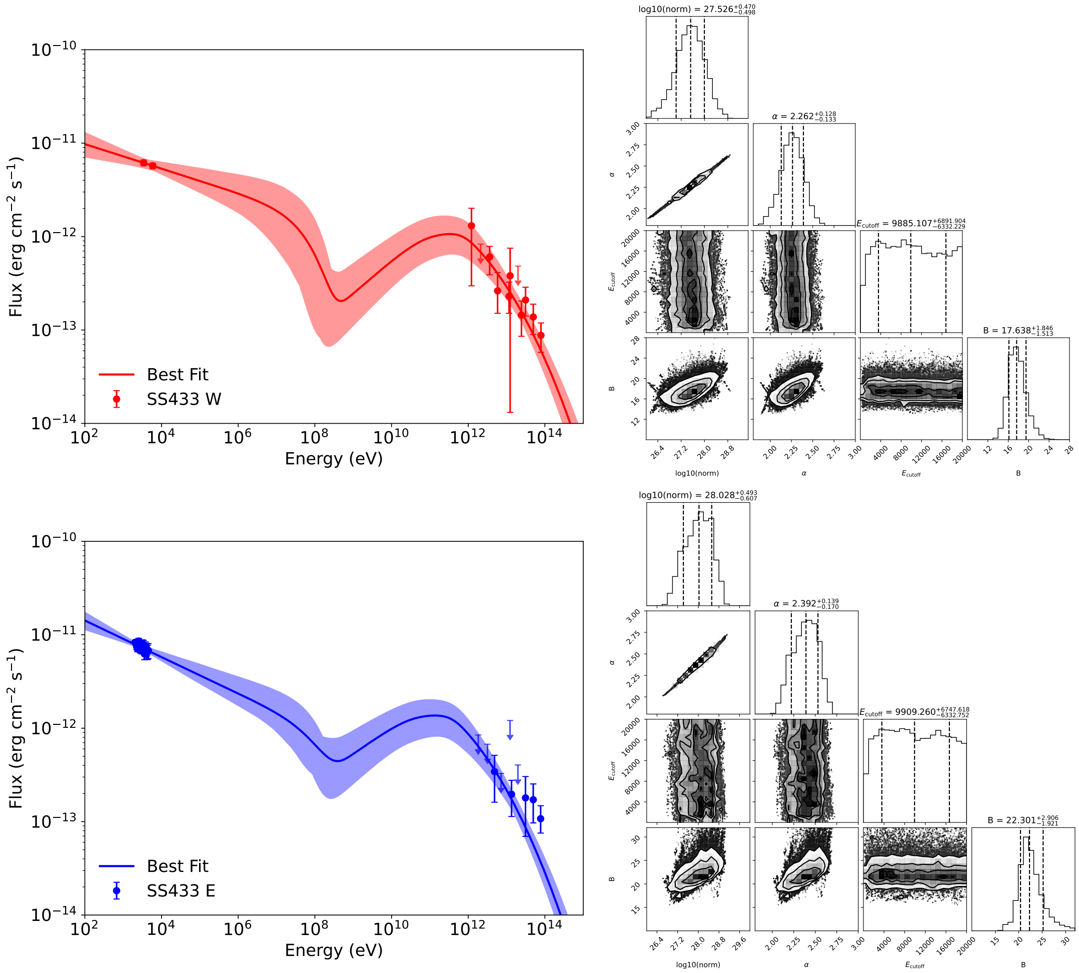

Supplementary Figure 10: Fitting to the multiwavelength spectra of the PWN of SS 433 W and E via synchrotron and IC radiations of electrons under the one-zone model. The solid curves represent the best-fit results while the bands show the 1σ uncertainties. Right columns show the corner plots from the MCMC method using the package *emcee*.

the projection distance) in the modeling. With this setup, we calculate the gamma-ray flux generated in the proton-proton collision between propagated protons and the cloud following a semi-analytical method developed in the literature[43]. The spatial distribution of protons is calculated . Since the emission of SS 433 below a few tens of TeV is probably dominated by the leptonic radiation, the hadronic flux must be reduced at low energies. It implies that protons of energy $\lesssim 100\,\mathrm{TeV}$ probably have not diffused to the cloud. More technically, the typical diffusion length (i.e., $\sqrt{4Dt_{\rm age}}$ of $100\,\mathrm{TeV}$ protons need be smaller than r_c, leading to a constraint $D(100\mathrm{TeV}) \lesssim 4\times10^{28}(r_c/100\,\mathrm{pc})^2(t_{\rm age}/20\,\mathrm{kyr})^{-1}\,\mathrm{cm^2/s}$. It should be noted that, as long as the distance of the accelerator from the cloud is the same, we would obtain similar proton distribution inside the cloud (and hence similar expected hadronic gamma-ray flux), no matter whether protons are injected at the BH vicinity or at the lobes. Note that if protons are accelerated at BH vicinity, relatively low-energy protons may suffer severe adiabatic loss as they are likely confined by expanding accretion winds and are therefore not injected into ambient medium. In this scenario, the predicted flux of the central extended at $< 10\,\mathrm{TeV}$ is quite low ($\lesssim 10^{-13}\,\mathrm{erg/cm^2\,s}$) and is consistent with the non-detection of such a central extended emission by H.E.S.S..

As shown in main text Figure 1e, a pure leptonic component cannot well explain the observed flux above $30\,\mathrm{TeV}$. For the lepto-hadronic scenario considered here, we employ the best-fit result but with $E_{\rm e,max} = 200\,\mathrm{TeV}$ (red dashed curve shown in the main text Figure 1e) as the leptonic componet, and we obtained a satisfactory fitting to the total emission of SS 433 by introducing a second hadronic component with $s_{\rm p} = 2$, $E_{\rm p,max} = 1\,\mathrm{PeV}$. Assuming $r_c = 100\,\mathrm{pc}$ and $t_{\rm age} = 20\,\mathrm{kyr}$, the diffusion coefficient is found to be $D(E_{\rm p}) \simeq 10^{28}(E_{\rm p}/100\,\mathrm{TeV})^{1/2}\,\mathrm{cm^2/s}$. This value is smaller than the average diffusion coefficient of the ISM by 2 orders of magnitude, and is about 10 times higher than the Bohm diffusion coefficient at $100\,\mathrm{TeV}$ (i.e., $D_{\rm Bohm} = 10^{27}(E_{\rm p}/100\,\mathrm{TeV}))(\mathrm{B}/3\mu\mathrm{G})\,\mathrm{cm^2/s}$. Such a condition is also found around other powerful CR sources such as the Cygnus Bubble [42]. It may be due to CR self-triggered instabilities or strongly perturbed interstellar magnetic field by the outflow launched from microquasar. The total proton luminosity above $1\,\mathrm{GeV}$ is $L_p \simeq 10^{39}\,\mathrm{erg/s}$. The kinetic powers of both two jets and the outflow of SS 433[44, 45, 46] exceed $\gtrsim 10^{39}\,\mathrm{erg/s}$, and hence the envisaged scenario is feasible from the perspective of the energy budget. Note that employed model parameters are not exclusive in this envisage picture. For example, we may also fit the SED data by locating the cloud closer to the accelerator (a smaller r_c), assuming a slower diffusion coefficient of particles (a smaller D), and correspondingly reducing the proton luminosity (a lower $L_{\rm p}$), or vice versa. Besides, the parameters of the hadronic component is also related to the parameters of the leptonic component. For example, if $E_{\rm e,max}$ is taken to be higher, or there exists a second leptonic component with a hard electron spectrum, the leptonic radiation may contribute a higher flux around $100\,\mathrm{TeV}$, and we then need to adjust the parameters of the hadronic component accordingly , such as a smaller proton injection luminosity.

2.1.5 PeV CRs from the entire Population of Microquasars harboring BHs

We use the PeV proton luminosity obtained from the lepto-hadronic scenario for SS 433 as a representative value. Given the total proton luminosity to be $10^{39}\,\mathrm{erg/s}$ and a spectral slope of -2, we may estimate the PeV proton luminosity of SS 433 to be $\sim 10^{38}\,\mathrm{erg/s}$. Theoretically, the total number of BH X-ray binaries in Milky Way is expected to be about 1000, with a dozen of them probably boasting an X-ray luminosity exceeding $10^{39}\mathrm{erg\ s^{-1}}$[47, 48], which is comparable to that of SS 433. If the relativistic proton luminosity scales with the X-ray luminosity of microquasars to certain extent, it is reasonable to expect that the total proton injection luminosity from all Galactic microquasars may exceed ~ 10 times that of SS 433 (i.e., $f_{\mu\rm Q} \sim 10$).

Following the leaky box model[49], we may derive the PeV CR proton flux at Earth as

$$
\begin{aligned}
F(E_{\rm p} = 1\,\mathrm{PeV}) &= \frac{c}{4\pi}\frac{L_{\rm p} f_{\mu\rm Q} t_{\rm res}}{2\pi R_{\rm Gal}^2 H_{\rm CR}} \\
&\approx 3\times10^4 \left(\frac{f_{\mu\rm Q} L_{\rm p,PeV}}{10^{39}\,\mathrm{erg\ s^{-1}}}\right)\left(\frac{D_{\rm ISM}}{10^{31}\,\mathrm{cm^2 s^{-1}}}\right)^{-1}\left(\frac{H_{\rm CR}}{4\,\mathrm{kpc}}\right)\left(\frac{R_{\rm Gal}}{15\,\mathrm{kpc}}\right)^{-2} \mathrm{eV\ cm^{-2}s^{-1}sr^{-1}},
\end{aligned}
$$

where $t_{\rm res} = H_{\rm CR}^2/4D_{\rm ISM}$ determines the PeV CR residence time within the Galaxy with $H_{\rm CR}$ being the scale height of the CR halo and $D_{\rm ISM}$ being the average diffusion coefficient of the ISM, and $R_{\rm Gal}$ is the typical radius of the Galaxy. The inferred PeV proton flux is consistent with the measurement[50]. A recent study[51] argues that the distribution of PeV CRs in the ISM may be clumpy and variable because of the

rarity of the sources. According to the simulation shown in the study, the observed PeV CR flux at Earth may be explained with a lower total injection luminosity of $\sim 10^{38}$ erg/s around PeV energy. With these simple estimations, we see that Galactic BH-jet systems can be potentially important factories of CRs around and above the knee.

2.2 V4641 Sgr

2.2.1 LHAASO Observation of V4641 Sgr

V4641 Sgr (RA=274.84°, DEC=-25.41° or $l = 6.77°$, $b = -4.79°$) hosts a 2.9±0.4$M_\odot$ companion star and a BH with mass of 6.4±0.6$M_\odot$[52]. V4641 Sgr can be observed by KM2A for about 3.3 hr per day with a zenith angle less than 60°, culminating at 55°. The ROI of KM2A used is a 8° × 8° square. Supplementary Figure 11 shows the significance maps around the V4641 Sgr at 25−250 TeV and >250 TeV. The 3D maximum likelihood method is used to produce these maps. The significance is about 6.9σ at 25−250 TeV and 8.1σ at >250 TeV. Assuming a 2D Gaussian template, the position and extension of the source obtained from the fitting is RA= 274.88° ± 0.09°, DEC= −25.69° ± 0.11°, $\sigma_{\rm ext}$=0.33° ± 0.08°. The position is very close to the position of V4641 Sgr (RA=274.840°, DEC=-25.407°) with an angular distance of 0.27°. If we correct the systematic shift in the DEC direction yielded by the Moon shadow result (see section 2.2.2), the measured position would be RA= 274.82° ± 0.09°, DEC= −25.60° ± 0.11° and the corresponding angular distance to the position of V4641 Sgr will be reduced to 0.19°. We have found that the pointing uncertainty will not affect the obtained extension of the source.

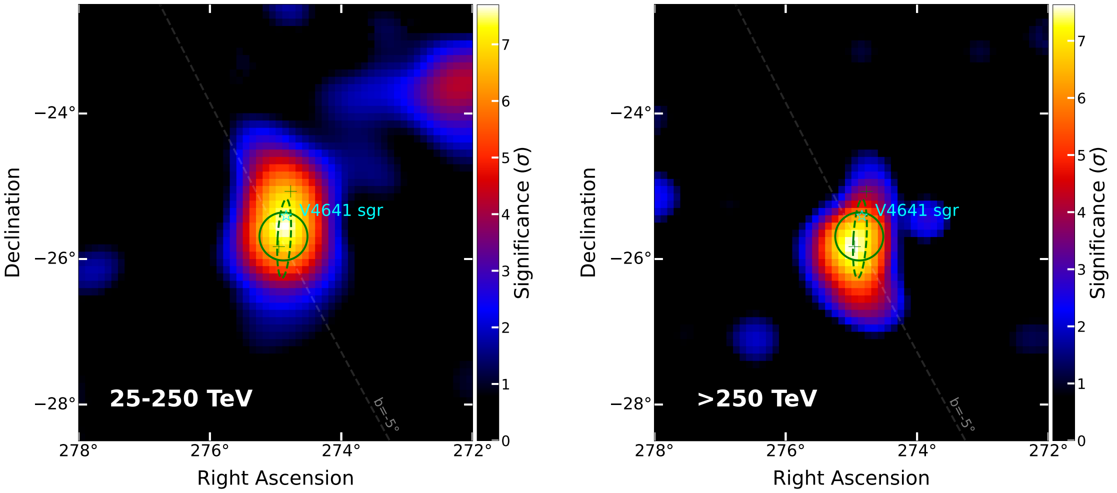

Supplementary Figure 11: Left: The significance map using events with energy 25−250 TeV. Right: The significance map using events with energy above 250 TeV. The 3D maximum likelihood method is used to produce the two maps. The cyan star marks the position of V4641 Sgr in other wavelengths. The green circle marks the position and extension of the source obtained from the KM2A data assuming a symmetric 2D Gaussian template. The results were obtained by assuming an asymmetric 2D Gaussian template and two point-source template, marked as dashed ellipse and two green crosses, respectively.

According to the significance map shown in Supplementary Figure 11, the morphology of V4641 Sgr deviates from the symmetric 2D Gaussian shape. Therefore, a asymmetric 2D Gaussian template is adopted to test the morphology. The parameters resulted from the fitting are $\sigma_{\rm ext,1} = 0.49° \pm 0.08°$ and $\sigma_{\rm ext,2} = 0.1° \pm 0.18°$. The improvement of the TS is 13.9, corresponding to 3.3σ. We also check two sources scenario, considering two point sources. The improvement of the TS is 8.0, corresponding to a significance of 2.0σ. Detailed information is listed in Supplementary Table 5 and also marked in Supplementary Figure 11. We also analyzed the extension of V4641 Sgr at two energy ranges, that is 25 − 250 TeV and >250 TeV. Using

a asymmetric 2D Gaussian template, the extension is $0.56° \pm 0.15°/< 0.20°$(95% C.L.) at 25−250 TeV, and $0.44° \pm 0.09°/0.12° \pm 0.09°$ at >250 TeV. The corresponding results are also shown in Supplementary Table 5.

**Model**	**RA**	**DEC**	**Extension ($\sigma_{\rm ext}$)**	**TS**
		One source scenario		
Gaussian	$274.88° \pm 0.09°$	$-25.69° \pm 0.11°$	$0.33° \pm 0.08°$	126.8
Ellipse Gaussian	$274.87° \pm 0.06°$	$-25.72° \pm 0.09°$	$0.49° \pm 0.08°/< 0.17°$(95% C.L.)	140.7
		Two sources scenario		
Point source	$274.95° \pm 0.08°$	$-25.83° \pm 0.09°$	-	134.8
Point Source	$274.77° \pm 0.09°$	$-25.07° \pm 0.11°$	-	
**Model(Energy range)**	**RA**	**DEC**	**Extension ($\sigma_{\rm ext}$)**	**Rotation angle**
Ellipse Gaussian (25-250TeV)	$274.89° \pm 0.09°$	$-25.44° \pm 0.13°$	$0.56° \pm 0.15°/< 0.20°$(95% C.L.)	$94.1° \pm 10.6°$
Ellipse Gaussian (>250TeV)	$274.91° \pm 0.07°$	$-25.89° \pm 0.12°$	$0.44° \pm 0.09°/0.12° \pm 0.09°$	$87.7° \pm 9.8°$

Supplementary Table 5: Morphology test above 25 TeV emission near the V4641 Sgr

Supplementary Figure 12 shows the spectral energy distribution. The spectrum is obtained with a symmetric 2D Gaussian template. This differential flux shown in Supplementary Figure 12 can be well fitted using a power-law function:

$$\frac{dN}{dE} = (2.23 \pm 0.30) \times 10^{-17} \left(\frac{E}{200\,\mathrm{TeV}}\right)^{-2.84\pm0.17} \mathrm{TeV}^{-1}\mathrm{cm}^{-2}\mathrm{s}^{-1}. \tag{12}$$

For comparison, the spectrum measured by HAWC [53] is shown in Supplementary Figure 12. The spectrum derived by LHAASO is consistent with the measurement of HAWC within the overlapping energy range.

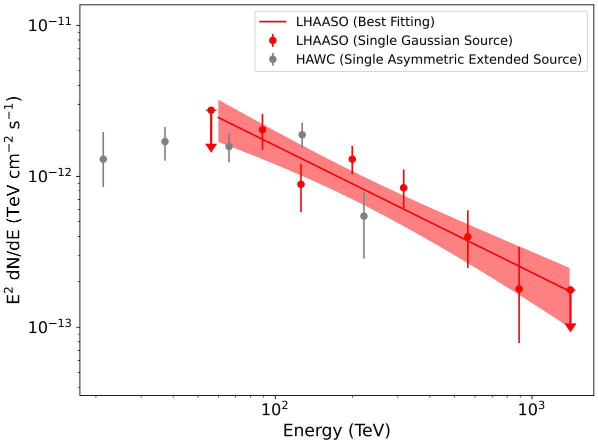

Supplementary Figure 12: The spectrum of V4641 Sgr obtained with a 2D Gaussian template, as shown with red circles. The spectrum measured by HAWC[53] (shown as grey circles) is shown for reference.

2.2.2 Systematic errors

In the standard data analysis pipeline of KM2A, only events with zenith angle less than 50° are used. Hence, only the sky region with DEC from -20° to 80° is observed. To extend the field of view of KM2A, events with zenith angle ranging from 50° to 60° are also used in this work for V4641 Sgr. Therefore, the sky region with declination from -30° to -20° including V4641 Sgr is covered. The performances of KM2A for these events with large zenith angles are checked using the standard sources, i.e., the Moon shadow and Crab Nebula.

Cosmic rays arrive at Earth in an almost isotropic manner. The Moon with a radius about 0.26° will block any cosmic ray arriving from that direction in the sky, and a deficit in the flux denoted as Moon shadow can be observed. The Moon shadow has been adopted to monitor on the performances of KM2A, including pointing and angular resolution[9]. The DEC of the Moon can vary from -28° to 28° [54]. To check the pointing error of KM2A for source with declination below -20°, the Moon shadow is analyzed when the declination of Moon ranges from -20° to -28°. Using the events with energies above 100 TeV, the position of the shadow yielded by the fitting is ΔRA =-0.052°±0.023°, ΔDEC =-0.078°± 0.026°. The deviation in the RA direction is effected by the Geomagnetic field and is roughly consistent with expectation (i.e., $1.59° Z(E/100\mathrm{TeV})^{-1}$, see ref.[10]), while the deviation in the DEC direction is at 3.0σ level. Supplementary Figure 13 shows the significance map of the Moon shadow. The significance is -18.1σ.

To check the possible systematic errors of KM2A for gamma-ray events with zenith angle ranging from 50° to 60°, the Crab Nebula is analyzed. Supplementary Figure 13 shows the significance map of Crab Nebula with energy above 25 TeV. The significance is 6.0σ. A 2D Gaussian is used to fit the excess event map, the position obtained by the fitting is RA=83.641°±0.092°, DEC =21.899°±0.092°. This position deviated from the expected position (83.633°, 22.015°) by ΔRA=-0.008°±0.092°, ΔDEC=-0.116°±0.092°. Due to large statistic errors, the deviation is not significant, and it is also consistent with the observation of the Moon Shadow within statistic errors. It is worth noting that the positions of the Crab Nebula and the Moon shadow are shifted in the DEC direction by a similar value with respect to their nominal positions. Hence, this pointing error derived from Moon shadow, with 0.08° is adopted as the systematic error of the position of V4641 Sgr.

To investigate the systematic bias for source extension measurement, we analyze the gamma-ray emission of LHAASO J1825-1326 using the events with zenith angle $\theta < 50°$ and the events with $50° < \theta < 60°$. The extension between these two data set is consistent, with the difference of $0.07° \pm 0.05°$. Thus, we adopt the potential PSF bias for data with zenith angle $\theta < 50°$ set as our extension systematic error, at the order of 0.08°, which is reported in Ref.[15].

Supplementary Figure 13 shows the SED of Crab Nebula using events with zenith angle above 50°, which is well consistent with that achieved using events with zenith angle below 50° in the overlapping energy range from 40 TeV to 200 TeV. Furthermore, systematic errors affecting the SED are considered as similar to that reported ref.[6], which is estimated to be 7% for the flux and 0.02 for the spectral index. In addition, an asymmetric 2D Gaussian shape is utilized to assess potential systematic errors, which leads to less than 2% difference. On the other hand, the influence of neighbouring sources can be ignored because we did not identify any other sources in the ROI. GDE in this region is not significantly detected. Adding a GDE template into the fitting results in a minor improvement of the overall TS value by only 2. The impact of GDE on the source flux is found to be less than 2%.

2.3 GRS 1915+105

GRS 1915+105 is located at RA= 288.80°, DEC= 10.94° ($l = 45.4°, b = -0.2°$), composed of a $10M_\odot$ BH and a K-type companion star of $0.5M_\odot$[55]. The ROI of used for the LHAASO analysis is a $7° \times 7°$ square, with RA ranging from 285.0° to 292.0° and DEC ranging from 8.0° to 15.0°. The significance map is shown as left panel in Supplementary Figure 14. The cyan star marks the position of GRS 1915+105. The GDE can also have significant contribution to the gamma-ray emissions in this region. In this work, the spectrum of GDE is assumed to follow a power-law function, characterized by the best-fit parameters $N_0 = 2.64 \pm 0.34 \times 10^{-14}$ TeV^{-1}cm^{2}s^{-1}sr^{-1} at $E_0 = 50$ TeV and $\alpha = 3.08 \pm 0.05$.

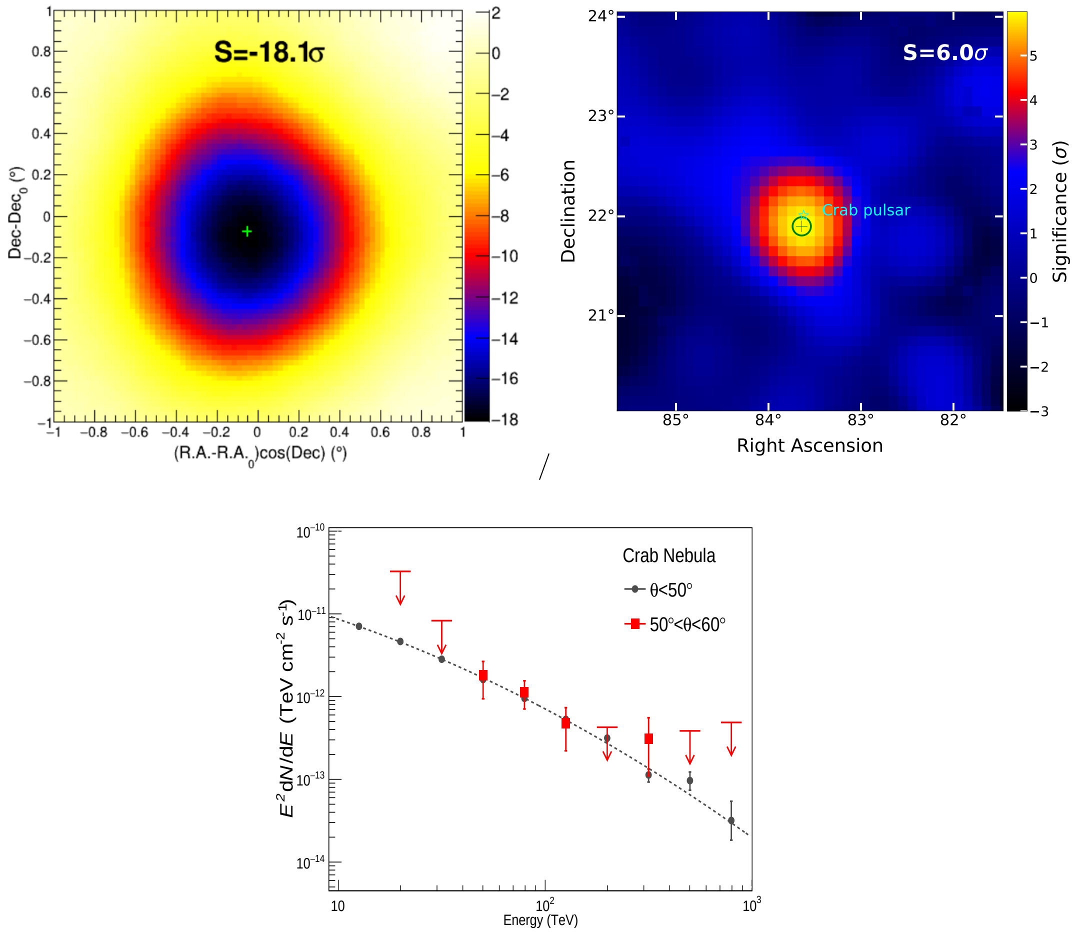

Supplementary Figure 13: Top left: The significance map of Moon Shadow using events with energy above 100 TeV when the declination of Moon ranges from -20° to -28°. The cross and the circle mark the position and corresponding error yielded by the fitting. Top right: The significance map of Crab Nebula using events with energy above 25 TeV and zenith angle ranging from 50° to 60°. The green cross and circle mark the position and corresponding error yielded by the fitting. The cyan star marks the position of Crab Pulsar. Bottom: The spectral energy distribution of the gamma-ray emission from Crab Nebula using events with different zenith angles.

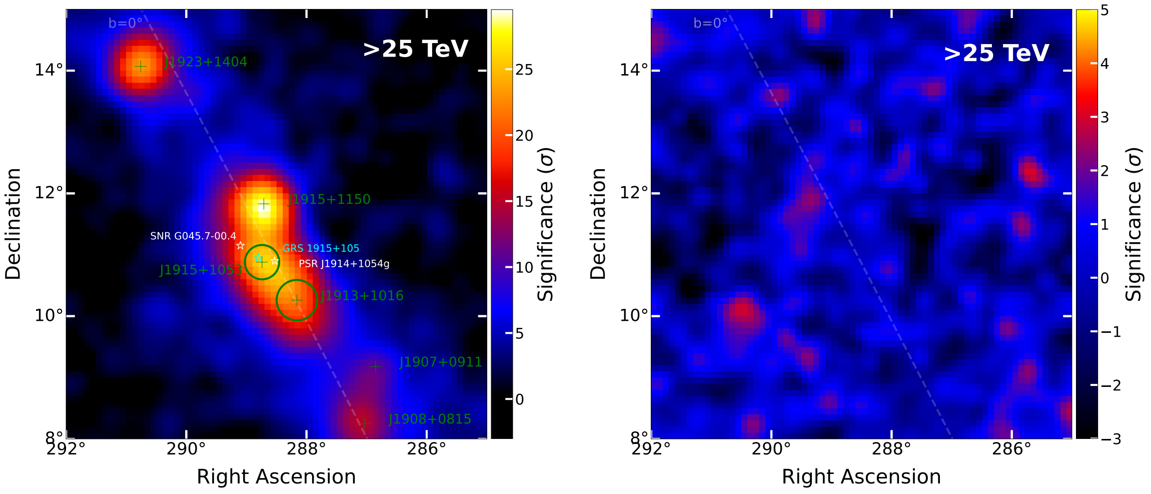

Supplementary Figure 14: Left panel: Significance map (> 25 TeV) of the ROI region for GRS 1915+105 (LHAASO J1915+1053). The green cross and circle indicate the best-fit position and 39% containment radius of the LHAASO sources, respectively. The cyan and white stars indicate the position of the counterparts. Right panel: Residual map of the ROI after subtracting all sources.

To test the number of sources within the ROI, we used an iterative method and identified 6 sources in the region. The spatial model of all sources are described using 2D Gaussian templates. For sources with small sizes ($\sigma < 0.2^\circ$), we perform point-like source testing. If $TS_{\rm ext} < 16$, the source's extension is considered insignificant, and the source is described using a point-like source model. These test results are summarized in Supplementary Table 6. The position of a new source is $(\mathrm{RA}, \mathrm{DEC} = 288.74^\circ \pm 0.05^\circ, 10.88^\circ \pm 0.05^\circ)$ with a 39% containment radius r_{39} of $0.28^\circ \pm 0.05^\circ$. We mark this new source as LHAASO J1915+1053 following the best-fit position. The best-fit position is shown in Supplementary Figure 14 as the green cross, while the green circle shows the best-fit extension . The right panel in Supplementary Figure 14 is the 2D residual significance map.

Supplementary Table 6: Best-fit model parameters of GRS 1915+101 region above 25 TeV.

Name LHAASO	RA (○)	DEC (○)	$\sigma_{\rm ext}$ (○)	significance (σ)	Possible Association
J1908+0811	287.03±0.05	8.18±0.06	ps	8.4	1LHAASO J1907+0826<br>2HWC J1907+084
J1907+0910	286.86±0.06	9.17±0.06	ps	5.8	new source
J1913+1016	288.16±0.06	10.26±0.07	0.33±0.04	14.2	1LHAASO J1912+1014u<br>HESS J1912+101
J1915+1053	288.74±0.05	10.88±0.05	0.28 ± 0.05	15.1	GRS 1915+105
J1915+1150	288.71±0.02	11.83±0.02	ps	19.7	1LHAASO J1914+1150u<br>2HWC J1914+117
J1923+1404	290.76±0.02	14.07±0.02	ps	17.3	1LHAASO J1922+1403<br>W 51

We assume the spectrum in the entire energy range to be a single power-law function as:

$$\frac{dN}{dE} = (0.48 \pm 0.11) \times 10^{-16} \left(\frac{E}{50\mathrm{TeV}}\right)^{-2.68\pm0.13} \mathrm{TeV^{-1}cm^{-2}s^{-1}} \tag{13}$$

The energy spectra of two other sources in the ROI also adopt the power-law functions. Based on the same method, the resulting systematic effect of extension of south source on the SED of GRS 1915+105 ranges in approximately $\pm(1\% - 15\%)$. The re-scaling of the GDE flux by $\pm20\%$ with respect to the best-fit value would cause an change of approximately $\pm(15\% - 35\%)$ on the SED of GRS 1915+105.

There are also other astrophysical candidates which are potentially associated with LHAASO J1915+1053. There are several pulsars in this region, but they either have low luminosities below 10^{33} erg/s or lack clear luminosity information, except for PSR J1914+1054g. PSR J1914+1054g is a radio pulsar discovered by FAST with a rotational period of about 140 ms, located at a distance of 7.8 kpc according to the dispersion measure[56]. MeerKAT measured the period's first derivative of the period and derived a spindown luminosity of 4×10^{35} erg/s and a characteristic age of 82 kyr of pulsar[57]. In principle, the pulsar may be responsible for the LHAASO source from the perspective of energy budget. However, there is an angular separation of 0.26° between the location of the pulsar and the centroid of the LHAASO source. The large spatial offset does not seem to support a pulsar origin for the TeV emission. Besides, spectra of pulsar wind nebulae or pulsar halos powered by middle-aged pulsars usually show clear cutoffs at $\lesssim 100$ TeV [58, 59, 60], while this source presents a power-law spectrum with an index of 2.68 up to 400 TeV without a cutoff feature. In Supplementary Figure 15, we compare the spectral shape of GRS 1915+105 with those of several typical middle-aged pulsars. Although the possible contribution from the pulsar-related source cannot be fully ruled out, GRS 1915+105 is likely the main contributor of the LHAASO source, in particularly at the UHE band.

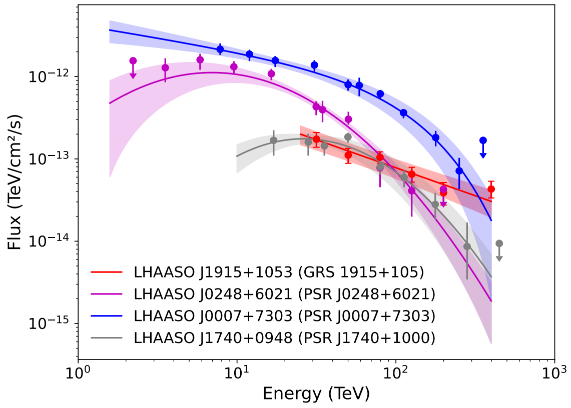

Supplementary Figure 15: Comparison of LHAASO J1915+1053 (GRS 1915+105), with spectra of middle-aged PWNe LHAASO J0248+6021 (PSR J0248+6021)[60], LHAASO J1740+0948 (PSR J1740+1000) [58] and LHAASO J0007+7303 (PSR J0007+7303, or CTA1 [59]). Unlike spectra of PWNe, LHAASO J1915+1053 does not exhibit a significant cutoff.

Supernova remnant (SNR) G045.7-00.4, located at northeast of GRS 1915+105, is another potential source of LHAASO J1915+1053. GeV gamma-ray emissions have been found from this middle-aged SNR and its vicinity[61]. Given the spatial association of GeV gamma rays to the molecular clouds at the same distance of the SNR, the GeV gamma rays were ascribed to pionic emissions of CR protons escaping from the SNR, as revealed by the gas distribution at the SNR distance using the data of the Milky Way Imaging Scroll Painting (MWISP) project[61]. Supplementary Figure 16 shows integrated CO emission map concentrated toward SNR G45.7-00.4 in the velocity interval of 52.0–62.0 km s^{-1}, which is suggested to be at the comparable distance of the SNR. However, the gas distribution shows a deficiency at the location of LHAASO J1915+1053. Thus, we conclude that LHAASO J1915+1053 does not originate from particles accelerated by the SNR.

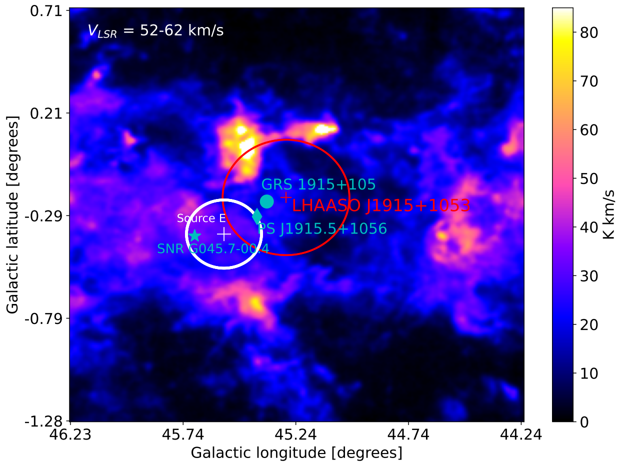

Supplementary Figure 16: Integrated ^{12}CO map toward SNR G045.7-00.4 in the interval of 52-62 km s^{-1}[61]. The blue star and dot indicate the positions of SNR G045.7-00.4 and GRS 1915+105, respectively. The red cross and circle represent the position and size of LHAASO J1915+1053. The cyan diamond represents the GeV source PS J1915.5+1056 [62]. The white cross and circle represent Source E, as identified in Zhang et al.([63]).

In the recent analysis of Fermi-LAT data by Martí-Devesa and Olivera-Nieto (2025) [62], the extended GeV source associated with SNR G045.7-00.4, as previously identified by Zhang et al. [63], was decomposed into an extended component and a new point-like source, PS J1915.5+1056. The latter was proposed as the GeV counterpart of GRS 1915+105. However, the evidence for this point-like source is not strong, as the model including both an extended source and a point-like source is only marginally better than the model with just an extended source, with $\Delta\text{AIC} \simeq -2$. It is possible that the emission attributed to PS J1915.5+1056 actually originates from the supernova remnant itself. Moreover, the position and extension of PS J1915.5+1056 are inconsistent with those of the LHAASO source. Therefore, we refrain from identifying it as the GeV counterpart of LHAASO J1915+1053 for the moment. In Figure 17, we compare the spectrum of PS J1915.5+1056 with those of the supernova remnant and the LHAASO source.

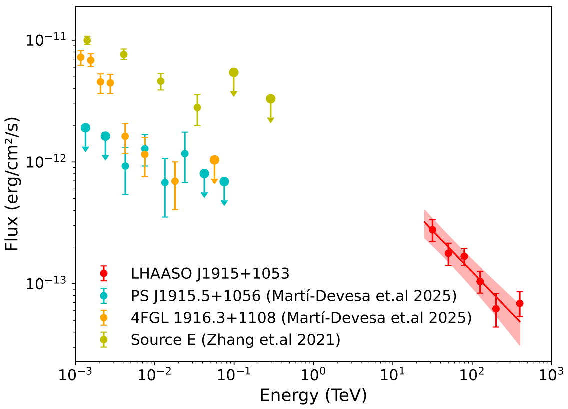

Supplementary Figure 17: The spectra of PS J1915.5+1056, 4FGL J1916.3+1108, Source E from Zhang et al. (2021) ([63]) and LHAASO J1915+1053.

2.4 Cygnus X-1

Cygnus X-1 is located in the bright and crowded Cygnus region, or more specifically, RA= 299.59°, DEC= 35.20° ($l = 71.33°, b = 3.07°$). It consists of a BH of $21M_\odot$[64] and an O-type companion star of $19M_\odot$[65]. Thus we used a large ROI for Cygnus X-1 to take into account the influence of the GDE and nearby bright gamma-ray sources. The ROI is defined with a longitude range of $l \in (60°, 85°)$ and a latitude range of $b \in (-5°, 5°)$ within the Galactic plane. The crowded region within a radius of 2.5° around LHAASO J2018+3651 is masked. Cygnus X-1 overlaps with two bright extended gamma-ray sources, Cygnus Bubble and LHAASO J2005+3204. The ROI is selected to encompass the main parts of these two sources. According to the Cygnus Bubble paper[66], its morphology is characterized by a combination of a 2D Gaussian template and the gas template which follows the column density distribution of atomic gas and molecular gas in the Cygnus region. Besides the Cygnus Bubble, LHAASO J2005+3204, sources listed in the 1st LHAASO catalog[15] within ROI and GDE are all included in the background model. A likelihood analysis is conducted to determine the significance of the source.

The TS value for a new point-like source around the position of Cygnus X-1 is 30. It corresponds to a significance of $4.4\,\sigma$, given four free parameters. An upper limit of the extension is found to be 0.22° at 95% C.L.. The upper limit of the extension is derived by calculating the TS values for different extension assumptions, with all the other parameters left free in the fitting. The 95% C.L. is obtained when the assumed extension leads to a decrease of the TS value by 2.7 with respect to the best-fit value. The best-fit position of the source is determined as RA = $299.47° \pm 0.13°$, DEC = $35.37° \pm 0.06°$. The spectrum is modeled by a power-law function as follows

$$\frac{dN}{dE} = (7.98 \pm 1.77) \times 10^{-18} \left(\frac{E}{50\,\mathrm{TeV}}\right)^{-3.98\pm0.40} \mathrm{TeV^{-1}cm^{-2}s^{-1}}, \tag{14}$$

The extension of the source is not significant. The spectrum of GDE is also modeled using a power-law function, with the fitting parameters being $N_0 = 1.05 \pm 0.06 \times 10^{-14}$ $\mathrm{TeV^{-1}cm^2s^{-1}sr^{-1}}$ and $\alpha = 2.95 \pm 0.04$ Furthermore, we analyzed the data for Cygnus X-1 above 40 TeV, dividing it into bins for each month, but found no signs of variability. The result is shown in Supplementary Figure 18.

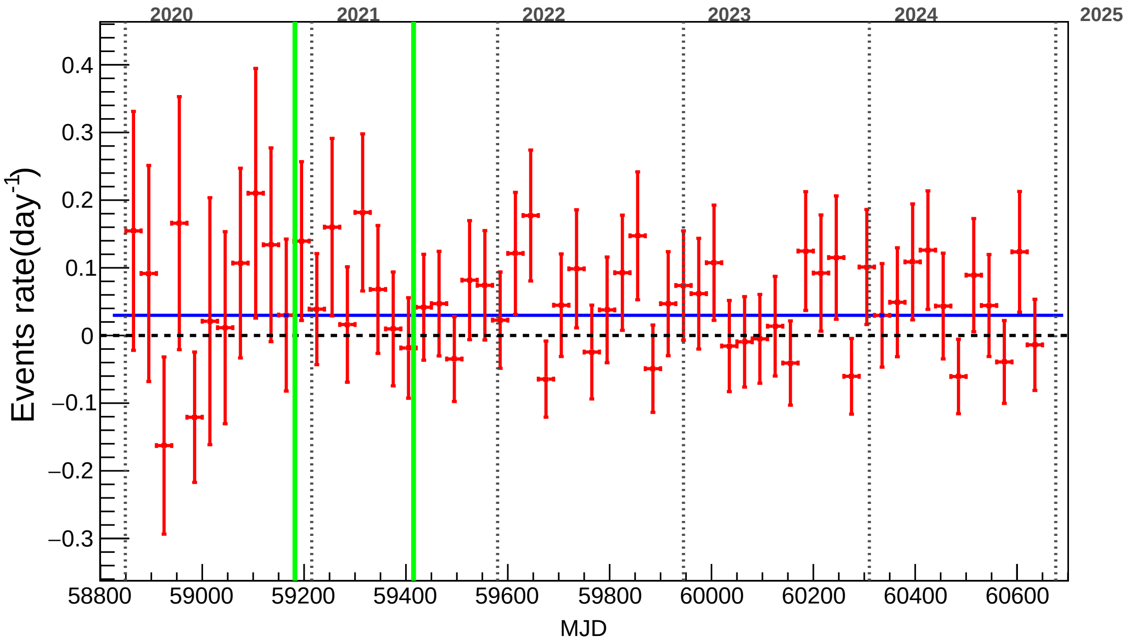

Supplementary Figure 18: Examination of variability of Cygnus X-1's emission above 40 TeV, with a bin width of one month. The vertical green lines show the time when the KM2A array was upgraded from 1/2 array to 3/4 array, and from 3/4 array to full array, respectively. The event rate during the 1/2 and 3/4 array have been normalized to the full array according to the effective area. The blue line indicates the fitting using a constant value which yields χ^2/ndf=42.5/59.

2.5 MAXI J1820+070

MAXI J1820+070 is located at RA= (275.09°, DEC= 7.18° ($l = 35.85°, b = 10.86°$), consisting of a BH of $8M_\odot$ and a K-type companion star of $0.6M_\odot$[67]. The ROI of our analysis is centered on MAXI J1820+070, encompassing a $4° \times 4°$ area. The ROI is in the high Galactic latitude area with $b = 10°$, making the background relatively clean. There are no other identified TeV sources in the ROI. The GDE component in the ROI of this source is also weak. If we fix spectral parameters of the GDE component to those measured from the entire inner Galactic disk [68], the TS value of this component is only 1.5, having little impact on the source fitting. If setting the spectral parameters of GDE free in the fitting, the obtained parameters would exhibit large uncertainties and cannot be constrained. So we do not perform a refit here. We employed a point source model to evaluate the significance of the source, treating its position as a free parameter. The resulting TS value for the source is 41.0, indicating a significance of approximately 6.0σ. The best-fit position is located at (RA, DEC) = $(275.22° \pm 0.05°, 7.39° \pm 0.06°)$, with a deviation of about 0.24° from MAXI J1820+070. The spectral model is assumed to be a power-law function, with best-fit parameters

$$\frac{dN}{dE} = (1.28 \pm 0.20) \times 10^{-17} \left(\frac{E}{50\,\mathrm{TeV}}\right)^{-3.24\pm0.26} \mathrm{TeV}^{-1}\mathrm{cm}^{-2}\mathrm{s}^{-1}. \quad (15)$$

Additionally, we examined the potential for the source to be extended by utilizing a 2D Gaussian model, which resulted in an increase of the TS value by only 1.2. Given one additional free parameter, the improvement of the fitting was not significant. Therefore, we consider the source to be point-like source and estimate the upper limit of its extension to be 0.28°.

Given the point-like nature of the source, in Supplementary Figure 19 we present the variability analysis for data above 25 TeV, providing the monthly binned event rate. No significant variability was detected.

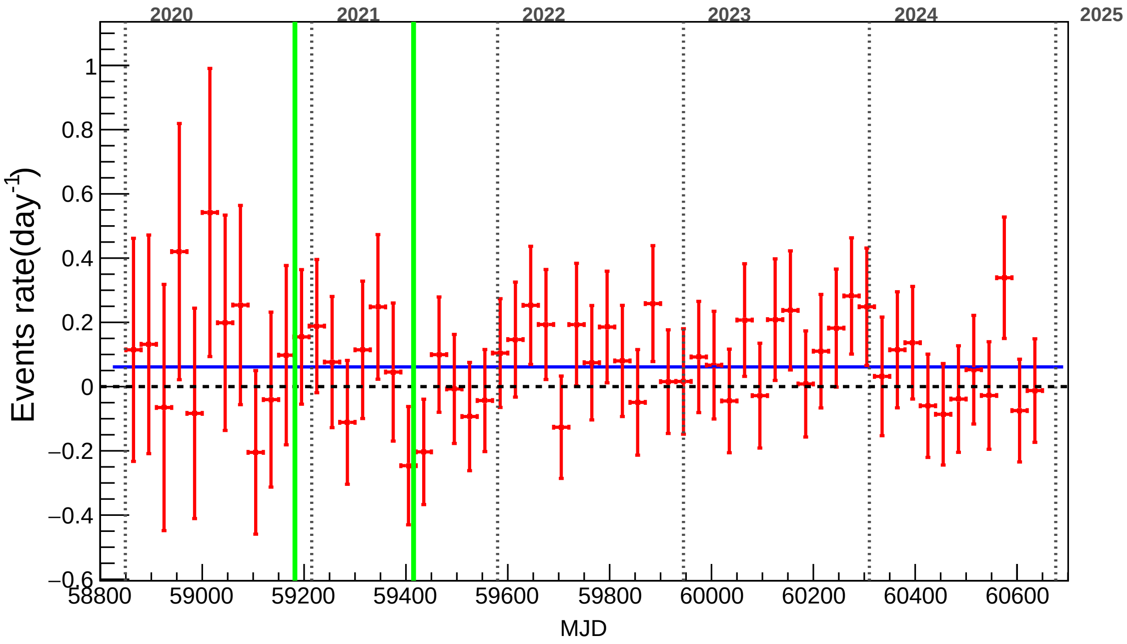

Supplementary Figure 19: Examination of variability of MAXI J1820+070's emission above 25 TeV. The width of each bin is 30 days. The vertical green lines show the time when the KM2A array was upgraded from 1/2 array to 3/4 array, and from 3/4 array to full array, respectively. The event rate during the 1/2 and 3/4 array have been normalized to the full array according to the effective area. The blue line indicates the fitting using a constant value which yields χ^2/ndf=30.9/59.

2.6 XTE J1859+226, XTE J1118+480, Swift J1727.8-1613, V404 Cygni, GRO J0422+32, GS 2000+251 and V616 Mon

For the other microquasars XTE J1859+226, XTE J1118+480, Swift J1727.8-1613, V404 Cygni, GRO J0422+32, GS 2000+251 and V616 Mon, a $4^\circ \times 4^\circ$ ROI was selected for each of them. We didn't find any significant signal around the position of these microquasars except V404 Cygni. The significance maps ($>$ 25 TeV) for these ROIs are shown in Supplementary Figure 20 and Supplementary Figure 21. For V404 Cygni, we found an excess in the significance map in the position of the microquasar. However, according to the first LHAASO catalog, there is an extended source LHAASO J2028+3352 located approximately 1.2° away from V404 Cygni, which leads to strong contamination to the gamma-ray emissions in the position of V404 Cygni. Using the standard 3D likelihood analysis, we treated both the spectral and spatial parameters of LHAASO J2028+3352, as well as spectral parameter of GDE, as free parameters. We found no significant excess at the position of V404 Cygni after subtracting the emission from LHAASO J2028+3352. The spectra of both LHAASO J2028+3352 and GDE were assumed to follow power-law functions. The morphology of LHAASO J2028+3352 was modeled using a 2D Gaussian template, with the best-fit position and size given by (RA, DEC) = $(306.99^\circ, 33.35^\circ)$ and $\sigma_{\rm ext} = 0.25^\circ$. We note that the astrophysical counterpart of LHAASO J2028+3352 is not clear yet, so V404 Cygni might in principle be its counterpart. However, given the large offset, we do not consider this possibility in this study. More dedicated study on the possible relation between V404 Cygni and LHAASO J2028+3352 is encouraged.

To obtain the flux upper limits of these microquasars, we locate a point-like source at the position of each of these binaries. The significance are all less than 3 σ even without considering the GDE component. The spectra of the sources are modeled using a power-law function with a fixed spectral index. Here, we considered three different spectral index ($\Gamma = -2.5, -3, -4$), and calculated the TS values for each, as shown in Supplementary Table 7. The set with the highest TS value, i.e., the case of $\Gamma = -4$, was used to compute the 95% flux upper limit and the resulting energy spectra upper limits are shown in Supplementary Figure

22.

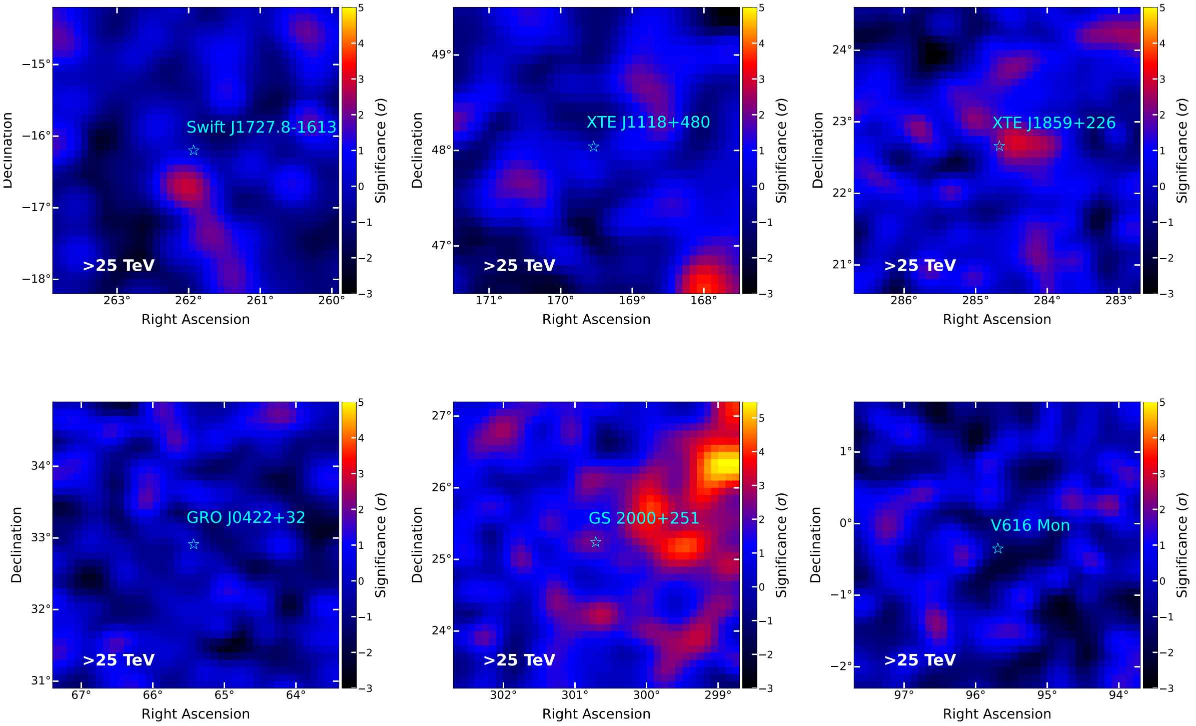

Supplementary Figure 20: The >25 TeV significance maps of Swift J1727.8-1613, XTE J1118+480, XTE J1859+226, GRO J0422+32, GS 2000+251 and V616 Mon. Cyan stars indicate the position of micro-quasars.

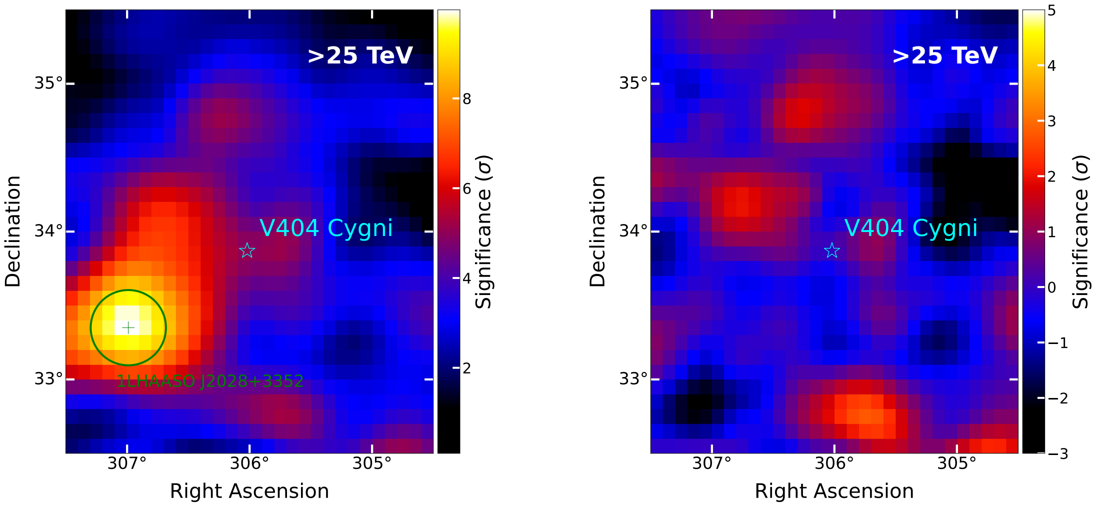

Supplementary Figure 21: The >25 TeV significance map (left) and residual map (right) in the ROI of V404 Cygni.

Supplementary Table 7: Spectral tests under different index values.

Swift J1727.8-1613		XTE J1118+408		XTE J1859+226	
Index	TS	Index	TS	Index	TS
2.5	0.00	2.5	0.00	2.5	1.71
3.0	0.00	3.0	0.00	3.0	4.65
4.0	0.53	4.0	0.18	4.0	7.27
**V404 Cygni**		**GRO J0422+32**		**GS 2000+251**	
Index	TS	Index	TS	Index	TS
2.5	0.41	2.5	0.00	2.5	3.94
3.0	1.37	3.0	0.00	3.0	5.12
4.0	2.12	4.0	0.43	4.0	3.54
		**V616 Mon**			
		Index	TS		
		2.5	0.00		
		3.0	0.00		
		4.0	0.15		

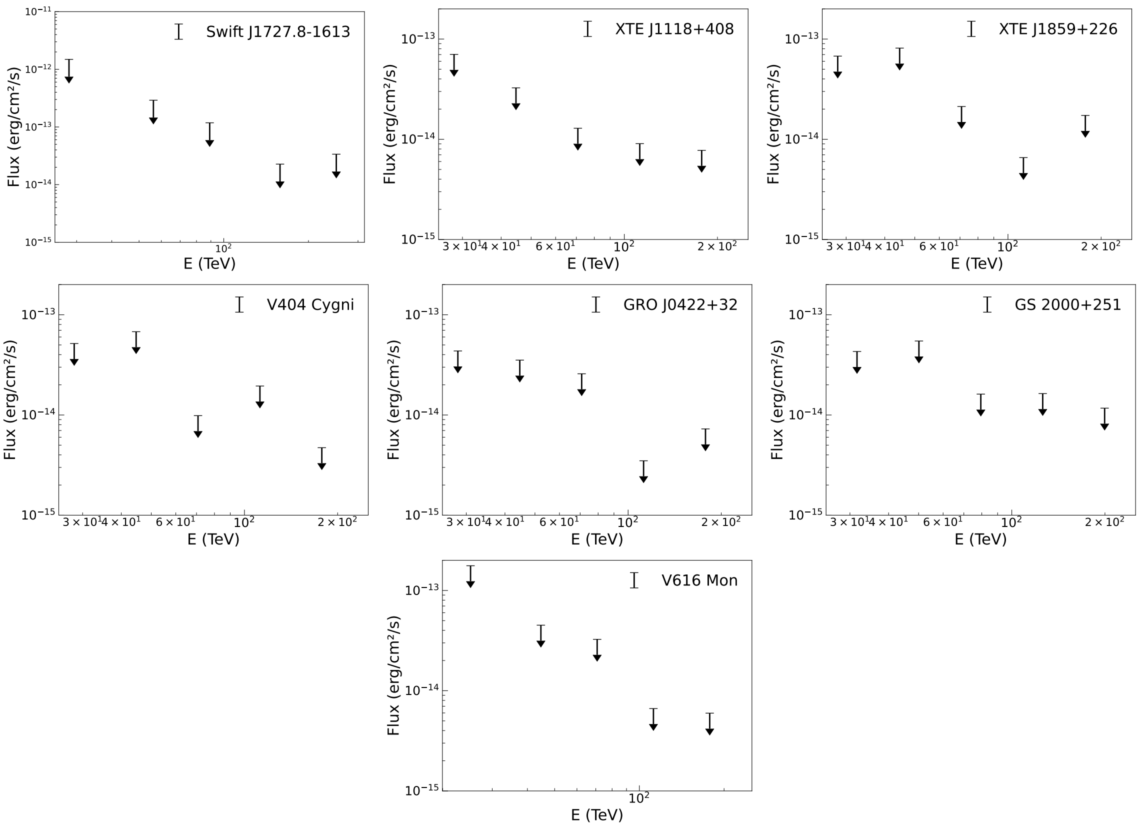

Supplementary Figure 22: 95% C.L. flux upper limits of Swift J1727.8-1613, XTE J1118+480, XTE J1859+226, V404 Cygni, GRO J0422+32, GS 2000+251 and V616 Mon, assuming a spectral index of -4.

2.7 Discussion on GDE

Supplementary Figure 23 shows the GDE spectra obtained in the ROI of the microquasars SS 433, GRS 1915+105 and Cygnus X-1, and compares them with the GDE spectra in the inner Galactic plane region ($15^\circ < l < 125^\circ$, $|b| < 5^\circ$) obtained in our previous work[13, 14]. The results indicate that the GDE intensities in the ROIs of SS 433 and GRS 1915+105 are significantly higher than that of the inner Galactic plane region. Note that GDE intensity is supposedly variable over different regions in the Galactic plane, depending on the

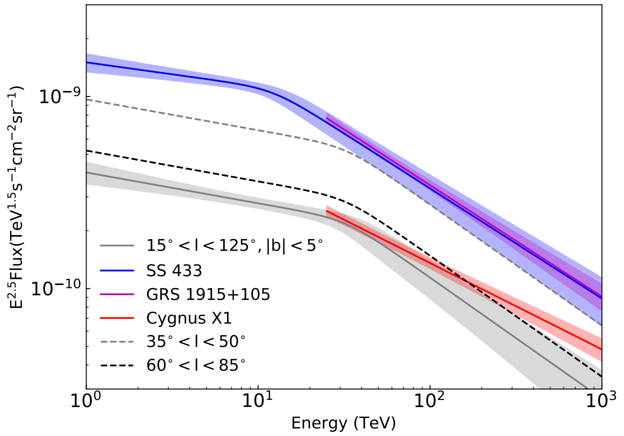

Supplementary Figure 23: GDE spectra measured from ROIs of SS 433, GRS 1915+105, and Cyngus X-1 compared with the GDE in the inner Galactic disk region. The gray error band represents the average GDE flux in the inner Galactic plane region ($15^\circ < l < 125^\circ$ and $|b| < 5^\circ$) given by ref. [14]. The dashed lines represent the GDE estimated for the ranges $35^\circ - 50^\circ$ and $60^\circ - 85^\circ$ based on Figure 4 in ref. [14].

average CR density and the gas column density in the region. For SS 433, the ROI spans approximately over $l \in [32^\circ,\ 48^\circ]$ when converted to the Galactic coordinate. For GRS 1915+105, ROI covers a range of approximately $l \in [40^\circ, 50^\circ]$. These regions coincide with the high-intensity region of GDE flux as can be seen in the 1D longitude profile of GDE reported in Figure 4 of ref.[14]. The variation in GDE shown here is therefore consistent with our previous measurement. We roughly estimated the GDE intensity in the regions of Galactic longitude $35^\circ - 50^\circ$ (approximately corresponding to region of SS 433/GRS 1915+101) and $60^\circ - 85^\circ$ (corresponding to region of Cygnus X-1) based on the ratio of diffuse fluxes (data points) to gas distributions (solid lines) shown in Figure 4 of ref.[14]. In the region of Galactic longitude $35^\circ - 50^\circ$, the GDE intensity is about 2.4 times higher than the Galactic average (gray error band), while in the region of $60^\circ - 85^\circ$, it is only about 1.3 times higher. The results are shown as dashed lines in Figure 23. Additionally, the distribution along the Galactic latitude also has some influence on the GDE intensity within the ROI. We did not significantly detect the GDE components in ROIs of V4641 Sgr and MAXI J1820+070 as mentioned in sections 2.2 and 2.5. The zenith angle of V4641 Sgr is large for LHAASO, so our effective area is suppressed as can be seen in Supplementary Figure 1. Therefore, we do not have sufficient statistics to identify the GDE component in the ROI and the GDE intensity in the ROI is not necessarily very weak. Nevertheless, the GDE is not expected to affect our measurement of V4641 Sgr given the latter's flux is very high. For MAXI J1820+070, the non-detection of GDE is mainly due to the weak emission of GDE in the ROI. MAXI J1820+070 is located at $b = 10^\circ$, so that the gas column density in the ROI is low.

3 Expectation in the X-ray band under the Leptonic Scenario

If UHE gamma-ray radiations of detected sources are dominated by the inverse Compton (IC) radiation of electrons accelerated by those microquasars, we would expect X-ray emission arising from synchrotron radiation of these electrons. Future X-ray observations may serve as a diagnostic tool of the magnetic fields in the UHE sources and facilitate our understanding their natures. Here we perform a phenomenological fitting to the spectrum of the sources measured by LHAASO under the leptonic scenario, and predict the corresponding synchrotron flux at the X-ray band for different magnetic field strength. Note that solely with LHAASO data, the spectral parameters cannot be well constrained. Therefore, instead of searching for best-fit parameters, we focus on the order-of-magnitude estimation of the X-ray flux, and hence simply employ an "eye-ball" fitting to the gamma-ray spectrum. In the model, we assume a (quasi-)steady-state electron spectrum in the form of

$$\frac{dN}{dE_{\rm e}} = N_0 E^{-p} e^{-(E/E_{\rm cut})^2} \tag{16}$$

Microquasars	p	$E_{\rm cut}$ (TeV)	$W_{\rm e}$ (ergs)	$L_e(B=20\mu{\rm G})$ (erg/s)	$L_e(B=5\mu{\rm G})$ (erg/s)	$L_e(B=1\mu{\rm G})$ (erg/s)
SS 433	1.5	300	2×10^{45}	2.7×10^{35}	1.9×10^{34}	2.6×10^{33}
V4641 Sgr	2.0	1000	8×10^{46}	1.3×10^{37}	8.7×10^{35}	8.3×10^{34}
GRS 1915+105	2.5	1000	6×10^{46}	1.2×10^{36}	8.3×10^{34}	1.1×10^{34}
MAXI J1820+070	2.8	500	4×10^{45}	2.6×10^{34}	1.9×10^{33}	3.1×10^{32}
Cygnus X-1	3.0	200	6×10^{45}	1.2×10^{34}	8.7×10^{32}	1.8×10^{32}

Supplementary Table 8: Electron spectral parameters employed in the fitting shown in Supplementary Figure 24, noting that parameters of SS 433 refers only to its hard component at the highest energies. Total energy ($W_{\rm e}$) and luminosity ($L_{\rm e}$) are for $E_{\rm e} > 25\,$TeV.

where N_0 is the normalisation factor, p is the spectral index, and $E_{\rm cut}$ is the cutoff energy in the spectrum. The normalisation factor N_0 can be obtained by $W_{\rm e} = \int E_{\rm e}(dN/dE_e)dE_e$, where $W_{\rm e}$ is a free parameter representing the total energy of electrons. The target radiation field of IC radiation includes the cosmic microwave background and the interstellar infrared radiation field. For the latter we follow the model given by ref. [37]. The $\gamma\gamma$ annihilation during the propagation of UHE photons is also taken into account and the opacity is calculated following the same target radiation field. We adjust the parameters of p, $E_{\rm cut}$ and $W_{\rm e}$ in order to fit the LHAASO data. Then we calculate the synchrotron radiation of electrons assuming three different magnetic field strength, i.e., $B = 20\,\mu$G, $5\,\mu$G and $1\,\mu$G, because the magnetic fields in the radiation zones of these sources are unclear. In addition, we may estimate the required electron injection luminosity by dividing the steady-state spectrum by the cooling timescales of electrons (including both synchrotron loss and IC loss), if a (quasi-)steady-state spectrum can be achieved (the cooling timescale is shorter than the dynamical timescale of the system but longer than the interval between two outbursts of the BH or two particle injection events). Since we focus on LHAASO-KM2A observations (which are carried out above 25 TeV) and the synchrotron emission of the same electrons responsible for LHAASO-KM2A observations, only electrons of energy above 25 TeV are taken into account. At 25 TeV, the cooling timescale of electrons is about 50 kyr even for the weakest magnetic field strength of $B = 1\,\mu$G considered here, so our assumption of a (quasi-)steady-state spectrum is reasonable. Note that, the obtained luminosity should be considered as the least value to maintain the observed gamma-ray flux since there should be electrons at lower energies. Here we do not extrapolate the spectrum to lower energies because the longer cooling timescales of lower-energy electrons may call into question the assumption of a quasi-steady state.

The fitting results of gamma-ray spectra and the predicted X-ray spectra are shown in Supplementary Figure 24. The electron spectral parameters are summarized in Supplementary Table 8. Note that, as discussed in the main text, the spectrum of SS 433 cannot be interpreted with a single leptonic component. Here, the first leptonic component is kept the same as considered in the main text (dashed red curve in the top-right panel of Supplementary Figure 24, supposedly from the two lobes), and we introduce a second leptonic component to fit the emission around 100 TeV. Note that the spectral index p represent the spectral slope in the (quasi-)steady state so the injection spectrum need be harder if we account for processes such as cooling and diffusion. For V4641 Sgr, we need a steady-state electron spectrum with a slope of $p = 2.0$ to explain the data under the leptonic scenario. Note that the radiative cooling timescale of ∼PeV electrons is only ∼10 kyr even in a magnetic field of $1\,\mu$G which is likely shorter than the dynamical timescale of the system. The steady-state spectrum has been modified by cooling, so $p = 2.0$ implies an extremely hard injection spectrum, unless the cooling of high-energy electrons is dominated by the IC loss in the Klein-Nishina regime[69, 70] (which may be achieved with $B \lesssim 0.5\mu$G for PeV electrons). The hard spectrum might be achieved in specific situations, such as the stochastic acceleration[71] (which requires a highly turbulent environment in the acceleration zone) or shear acceleration[72] (which requires a significant bulk velocity shear of the background flow in the acceleration zone).

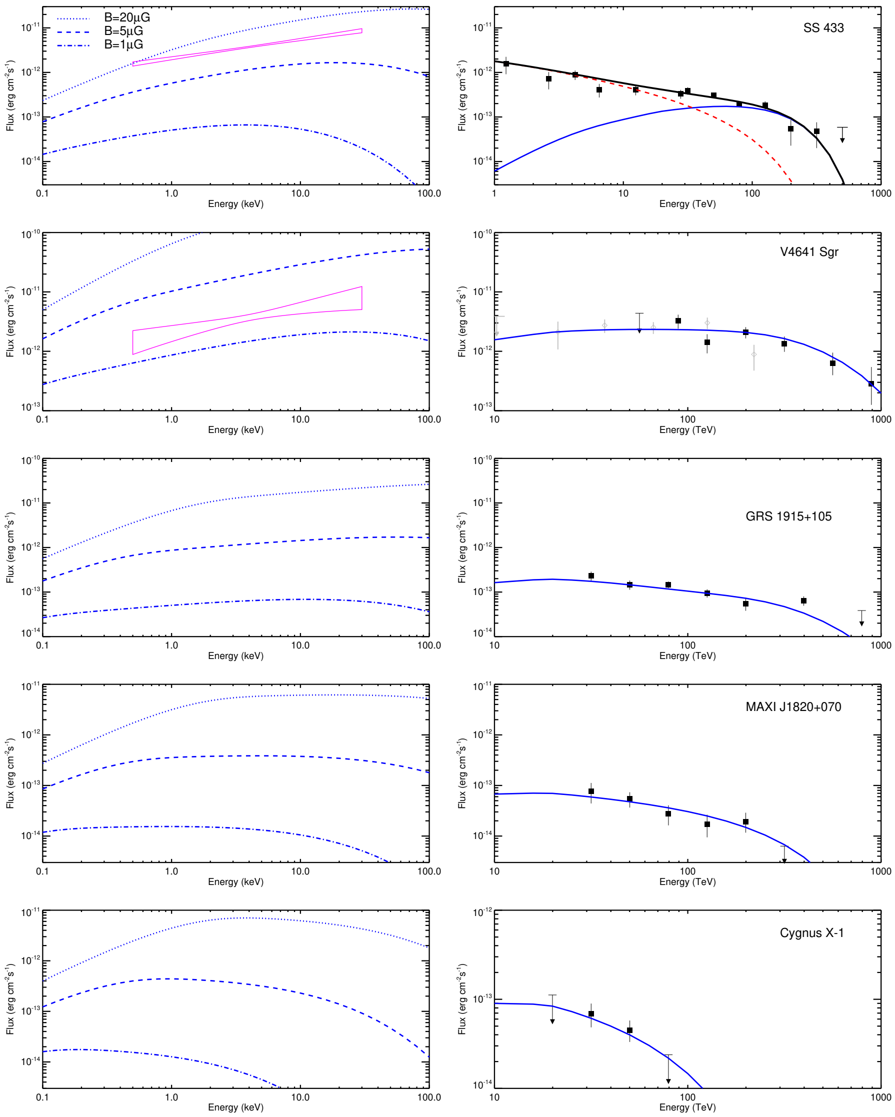

Supplementary Figure 24: Expected synchrotron X-ray fluxes of five detected sources (the left column) when interpreting the gamma-ray emission with the inverse Compton radiation of electrons (the right column, shown as solid blue curve). From top to bottom, each row shows the results for SS 433, V4641 Sgr, GRS 1915+105, MAXI J1820+070 and Cygnus X-1. In each panel of the left column, the dotted, dashed, dot-dashed curve represent the cases of magnetic field $B = 20\,\mu$G, $5\,\mu$G and $1\,\mu$G, respectively. In the top right panel (in the case of SS 433), the dashed red curve is the same as the one shown in the main text Figure 1e, and we introduce a second leptonic component to explain the spectrum at the high-energy end. The solid black curve is the sum of the dashed red curve and the solid blue curve. In the top left panel, only the synchrotron flux of the second leptonic component is shown. The magenta bowties in left-hand panel of the first row and the second row showcase the X-ray flux measured from the "head" of the east lobe of SS 433[73] and from an extended source around V4641 Sgr[74], respectively. We caveat that these fluxes are shown here just for reference, and should not be compared with the model prediction directly, because the fluxes are from regions much smaller than corresponding LHAASO sources.

References

[1] Cao, Z. et al. The Large High Altitude Air Shower Observatory (LHAASO) Science Book (2021 Edition). arXiv e-prints arXiv:1905.02773 (2019). 1905.02773.

[2] Heck, D., Knapp, J., Capdevielle, J. N., Schatz, G. & Thouw, T. CORSIKA: A Monte Carlo code to simulate extensive air showers (1998).

[3] Chen, S. et al. Design of the detector simulation of lhaaso-km2a. Hedianzixue Yu Tance Jishu/Nuclear Electronics and Detection Technology **37**, 1101–1105 (2017).

[4] Cao, Z. et al. Lhaaso-km2a detector simulation using geant4. Radiation Detection Technology and Methods **8**, 1437–1447 (2024). URL http://dx.doi.org/10.1007/s41605-024-00467-8.

[5] Agostinelli, S. et al. Geant4—a simulation toolkit. Nuclear Instruments and Methods in Physics Research Section A **506**, 250–303 (2003). URL https://www.sciencedirect.com/science/article/pii/S0168900203013688.

[6] Aharonian, F. et al. Observation of the Crab Nebula with LHAASO-KM2A - a performance study. Chinese Physics C **45**, 025002 (2021). 2010.06205.

[7] Cao, Z. et al. Optimization of performance of the KM2A full array using the Crab Nebula. Chinese Physics C **48**, 065001 (2024). 2401.01038.

[8] Lhaaso Collaboration et al. Peta-electron volt gamma-ray emission from the Crab Nebula. Science **373**, 425–430 (2021). 2111.06545.

[9] Cao, Z. et al. Data quality control system and long-term performance monitor of LHAASO-KM2A. Astroparticle Physics **164**, 103029 (2025). 2405.11826.

[10] Aharonian, F. et al. Performance of lhaaso-wcda and observation of the crab nebula as a standard candle *. Chinese Physics C **45**, 085002 (2021). URL https://dx.doi.org/10.1088/1674-1137/ac041b.

[11] Abeysekara, A. U. et al. Observation of the crab nebula with the hawc gamma-ray observatory. The Astrophysical Journal **843**, 39 (2017). URL https://dx.doi.org/10.3847/1538-4357/aa7555.

[12] Fleysher, R., Fleysher, L., Nemethy, P., Mincer, A. I. & Haines, T. J. Tests of statistical significance and background estimation in gamma-ray air shower experiments. The Astrophysical Journal **603**, 355 (2004). URL https://dx.doi.org/10.1086/381384.

[13] Cao, Z. et al. Measurement of Ultra-High-Energy Diffuse Gamma-Ray Emission of the Galactic Plane from 10 TeV to 1 PeV with LHAASO-KM2A. Phys. Rev. Lett. **131**, 151001 (2023). 2305.05372.

[14] Cao, Z. et al. Measurement of Very-High-Energy Diffuse Gamma-Ray Emissions from the Galactic Plane with LHAASO-WCDA. Phys. Rev. Lett. **134**, 081002 (2025). 2411.16021.

[15] Cao, Z. et al. The First LHAASO Catalog of Gamma-Ray Sources. Astrophys. J. Supp. **271**, 25 (2024). 2305.17030.

[16] Planck Collaboration et al. Planck 2013 results. XI. All-sky model of thermal dust emission. Astron. Astrophys. **571**, A11 (2014). 1312.1300.

[17] Hillwig, T. C. & Gies, D. R. Spectroscopic Observations of the Mass Donor Star in SS 433. Astrophys. J. Lett. **676**, L37 (2008). 0711.4348.

[18] Cherepashchuk, A. M., Belinski, A. A., Dodin, A. V. & Postnov, K. A. Discovery of orbital eccentricity and evidence for orbital period increase of SS433. Mon. Not. R. Astron. Soc. **507**, L19–L23 (2021). 2107.09005.

[19] H. E. S. S. Collaboration, Olivera-Nieto, L., Reville, B., Hinton, J. & Tsirou, M. Acceleration and transport of relativistic electrons in the jets of the microquasar SS 433. Science **383**, 402–406 (2024).

[20] Cao, Z. et al. Ultrahigh-energy photons up to 1.4 petaelectronvolts from 12 γ-ray Galactic sources. Nature **594**, 33–36 (2021).

[21] Middleton, M. J. et al. NuSTAR reveals the hidden nature of SS433. Mon. Not. R. Astron. Soc. **506**, 1045–1058 (2021). 1810.10518.

[22] Peek, J. E. G. et al. The GALFA-HI Survey: Data Release 1. Astrophys. J. Supp. **194**, 20 (2011). 1101.1879.

[23] Peek, J. E. G. et al. The GALFA-H I Survey Data Release 2. Astrophys. J. Supp. **234**, 2 (2018).

[24] Su, Y. et al. The Large-scale Interstellar Medium of SS 433/W50 Revisited. Astrophys. J. **863**, 103 (2018). 1807.03737.

[25] Li, J. et al. Gamma-ray heartbeat powered by the microquasar SS 433. Nature Astronomy **4**, 1177–1184 (2020).

[26] Luri, X. et al. Gaia Data Release 2. Using Gaia parallaxes. Astron. Astrophys. **616**, A9 (2018).

[27] Stirling, A. M. et al. Radio-emitting component kinematics in SS433. Mon. Not. R. Astron. Soc. **337**, 657–665 (2002).

[28] Blundell, K. M. & Bowler, M. G. Symmetry in the Changing Jets of SS 433 and Its True Distance from Us. Astrophys. J. Lett. **616**, L159–L162 (2004). astro-ph/0410456.

[29] Picchi, P., Shore, S. N., Harvey, E. J. & Berdyugin, A. An optical spectroscopic and polarimetric study of the microquasar binary system SS 433. Astron. Astrophys. **640**, A96 (2020). 2007.09615.

[30] Panferov, A. Simulation of kinematics of SS 433 radio jets that interact with the ambient medium. Astron. Astrophys. **562**, A130 (2014). 1306.3599.

[31] Lockman, F. J., Blundell, K. M. & Goss, W. M. The distance to SS433/W50 and its interaction with the interstellar medium. Mon. Not. R. Astron. Soc. **381**, 881–893 (2007). 0707.0506.

[32] HI4PI Collaboration et al. HI4PI: A full-sky H I survey based on EBHIS and GASS. Astron. Astrophys. **594**, A116 (2016). 1610.06175.

[33] Bosch-Ramon, V., Aharonian, F. A. & Paredes, J. M. Electromagnetic radiation initiated by hadronic jets from microquasars in the ISM. Astron. Astrophys. **432**, 609–618 (2005). astro-ph/0411508.

[34] Aharonian, F. A. & Atoyan, A. M. On the emissivity of $\pi\hat{0}\tilde{}$-decay gamma radiation in the vicinity of accelerators of galactic cosmic rays. Astron. Astrophys. **309**, 917–928 (1996).

[35] Zirakashvili, V. N. & Aharonian, F. Analytical solutions for energy spectra of electrons accelerated by nonrelativistic shock-waves in shell type supernova remnants. Astron. Astrophys. **465**, 695–702 (2007). astro-ph/0612717.

[36] Abeysekara, A. U. et al. Very-high-energy particle acceleration powered by the jets of the microquasar SS 433. Nature **562**, 82–85 (2018).

[37] Popescu, C. C. et al. A radiation transfer model for the Milky Way: I. Radiation fields and application to high-energy astrophysics. Mon. Not. R. Astron. Soc. **470**, 2539–2558 (2017). 1705.06652.

[38] Zealey, W. J., Dopita, M. A. & Malin, D. F. The interaction between the relativistic jets of SS433 and the interstellar medium. Mon. Not. R. Astron. Soc. **192**, 731–743 (1980).

[39] Goodall, P. T., Alouani-Bibi, F. & Blundell, K. M. When microquasar jets and supernova collide: hydrodynamically simulating the SS 433-W 50 interaction. Mon. Not. R. Astron. Soc. **414**, 2838–2859 (2011). 1101.3486.

[40] Han, Q. & Li, X.-D. On the Formation of SS433. Astrophys. J. **896**, 34 (2020). 2004.12547.

[41] Zabalza, V. Naima: a Python package for inference of particle distribution properties from nonthermal spectra. In 34th International Cosmic Ray Conference (ICRC2015), vol. 34 of International Cosmic Ray Conference, 922 (2015). 1509.03319.

[42] LHAASO Collaboration. An ultrahigh-energy γ-ray bubble powered by a super pevatron. Science Bulletin **69**, 449–457 (2024).

[43] Kafexhiu, E., Aharonian, F., Taylor, A. M. & Vila, G. S. Parametrization of gamma-ray production cross sections for p p interactions in a broad proton energy range from the kinematic threshold to PeV energies. Phys. Rev. D **90**, 123014 (2014). 1406.7369.

[44] Marshall, H. L., Canizares, C. R. & Schulz, N. S. The High-Resolution X-Ray Spectrum of SS 433 Using the Chandra HETGS. Astrophys. J. **564**, 941–952 (2002). astro-ph/0108206.

[45] Middleton, M. J. et al. NuSTAR reveals the hidden nature of SS433. Mon. Not. R. Astron. Soc. **506**, 1045–1058 (2021). 1810.10518.

[46] Fogantini, F. A. et al. A NuSTAR view of SS433. Precessional evolution of the jet-disk system. Astron. Astrophys. **669**, A149 (2023). 2210.09390.

[47] Shao, Y. & Li, X.-D. Population Synthesis of Black Hole X-Ray Binaries. Astrophys. J. **898**, 143 (2020).

[48] Kantzas, D. et al. Possible contribution of X-ray binary jets to the Galactic cosmic ray and neutrino flux. Mon. Not. R. Astron. Soc. **524**, 1326–1342 (2023). 2306.12715.

[49] Ginzburg, V. L. & Syrovatskii, S. I. The Origin of Cosmic Rays (1964).

[50] Apel, W. D. et al. KASCADE-Grande measurements of energy spectra for elemental groups of cosmic rays. Astroparticle Physics **47**, 54–66 (2013).

[51] Giacinti, G. & Semikoz, D. Model of Cosmic Ray Propagation in the Milky Way at the Knee. arXiv e-prints arXiv:2305.10251 (2023). 2305.10251.

[52] MacDonald, R. K. D. et al. The Black Hole Binary V4641 Sagitarii: Activity in Quiescence and Improved Mass Determinations. Astrophys. J. **784**, 2 (2014). 1401.4190.

[53] Alfaro, R. et al. Ultra-high-energy gamma-ray bubble around microquasar V4641 Sgr. Nature **634**, 557–560 (2024). 2410.16117.

[54] Wen, T., Chen, S. & Dai, B. The Influence of the Sun and Moon on the Observation of Very High Energy Gamma-ray Sources Using EAS Arrays. Research in Astronomy and Astrophysics **24**, 065020 (2024). 2405.12492.

[55] Steeghs, D. et al. The Not-so-massive Black Hole in the Microquasar GRS1915+105. Astrophys. J. **768**, 185 (2013). 1304.1808.

[56] Han, J. L. et al. The FAST Galactic Plane Pulsar Snapshot survey: I. Project design and pulsar discoveries. Research in Astronomy and Astrophysics **21**, 107 (2021). 2105.08460.

[57] Motta, S. E. et al. MeerKAT caught a Mini Mouse: serendipitous detection of a young radio pulsar escaping its birth site. Mon. Not. R. Astron. Soc. **523**, 2850–2857 (2023). 2305.06130.

[58] Lhaaso Collaboration et al. Ultra-high-energy γ-ray emission associated with the tail of a bow-shock pulsar wind nebula. The Innovation **6**, 100802 (2025).

[59] Lhaaso Collaboration et al. Deep view of composite SNR CTA1 with LHAASO in γ-rays up to 300 TeV. Science China Physics, Mechanics, and Astronomy **68**, 279503 (2025).

[60] Cao, Z. et al. LHAASO detection of very-high-energy γ-ray emission surrounding PSR J0248+6021. Science China Physics, Mechanics, and Astronomy **68**, 279504 (2025).

[61] Zhang, H.-M. et al. Fermi-LAT Detection of Extended Gamma-Ray Emission in the Vicinity of SNR G045.7-00.4: Evidence of Escaping Cosmic Rays Interacting with the Surrounding Molecular Clouds. Astrophys. J. **923**, 106 (2021). 2111.03774.

[62] Martí-Devesa, G. & Olivera-Nieto, L. Persistent GeV Counterpart to the Microquasar GRS 1915+105. Astrophys. J. Lett. **979**, L40 (2025). 2410.10396.

[63] Zhang, H.-M. et al. Fermi-LAT Detection of Extended Gamma-Ray Emission in the Vicinity of SNR G045.7-00.4: Evidence of Escaping Cosmic Rays Interacting with the Surrounding Molecular Clouds. Astrophys. J. **923**, 106 (2021). 2111.03774.

[64] Miller-Jones, J. C. A. et al. Cygnus X-1 contains a 21-solar mass black hole—Implications for massive star winds. Science **371**, 1046–1049 (2021). 2102.09091.

[65] Orosz, J. A. et al. The Mass of the Black Hole in Cygnus X-1. Astrophys. J. **742**, 84 (2011). 1106.3689.

[66] Lhaaso Collaboration. An ultrahigh-energy γ -ray bubble powered by a super PeVatron. Science Bulletin **69**, 449–457 (2024).

[67] Torres, M. A. P. et al. The Binary Mass Ratio in the Black Hole Transient MAXI J1820+070. Astrophys. J. Lett. **893**, L37 (2020). 2003.02360.

[68] Cao, Z. et al. The First LHAASO Catalog of Gamma-Ray Sources. Astrophys. J. Supp. **271**, 25 (2024). 2305.17030.

[69] Aharonian, F. A. & Ambartsumyan, A. S. Formation of the spectrum of superhigh energy electrons in the galaxy. Astrophysics **23**, 650–654 (1985).

[70] Breuhaus, M. et al. Ultra-high Energy Inverse Compton Emission from Galactic Electron Accelerators. Astrophys. J. Lett. **908**, L49 (2021). 2010.13960.

[71] Schlickeiser, R. An explanation of abrupt cutoffs in the optical-infrared spectra of non-thermal sources. A new pile-up mechanism for relativistic electronspectra. Astron. Astrophys. **136**, 227–236 (1984).

[72] Berezhko, E. G. & Krymskii, G. F. a Kinetic Analysis of the Charged Particle Acceleration Process in Collisionless Plasma Shear Flows. Soviet Astronomy Letters **7**, 352 (1981).

[73] Safi-Harb, S. et al. Hard X-Ray Emission from the Eastern Jet of SS 433 Powering the W50 "Manatee" Nebula: Evidence for Particle Reacceleration. Astrophys. J. **935**, 163 (2022). 2207.00573.

[74] Suzuki, H. et al. Detection of Extended X-Ray Emission around the PeVatron Microquasar V4641 Sgr with XRISM. Astrophys. J. Lett. **978**, L20 (2025). 2412.08089.
